# Discrimination and distress among Afghan refugees in northern California: The moderating role of pre- and post-migration factors

**DOI:** 10.1371/journal.pone.0196822

**Published:** 2018-05-21

**Authors:** Qais Alemi, Carl Stempel

**Affiliations:** 1 Loma Linda University, Department of Social Work and Social Ecology, School of Behavioral Health, Loma Linda, CA, United States of America; 2 California State University, East Bay, Department of Sociology and Social Services, Hayward, CA, United States of America; University of New South Wales, AUSTRALIA

## Abstract

This study investigates the effect of perceived discrimination on the mental health of Afghan refugees, and secondly, tests the distress moderating effects of pre-migration traumatic experiences and post-resettlement adjustment factors. In a cross-sectional design, 259 Afghans completed surveys assessing perceived discrimination and a number of other factors using scales developed through inductive techniques. Multivariable analyses consisted of a series of hierarchical regressions testing the effect of perceived discrimination on distress, followed by a sequential analysis of moderator variables. Perceived discrimination was significantly associated with higher distress, and this relationship was stronger among those with a strong intra-ethnic identity and high pre-resettlement traumatic experiences. The expected buffering effects of civic engagement, ethnic orientation (e.g. integration), and social support were not significant. Discrimination is a significant source of stress for Afghan refugees, which may exacerbate stresses associated with other pre- and post-migration stressors. Future research is needed to tailor interventions that can help mitigate the stress associated with discrimination among this highly vulnerable group.

## Introduction

Afghanistan remains a major source country for refugees with over 2.6 million Afghans currently residing in Iran and Pakistan, and thousands spread over 70 countries including the United States (U.S.) [1]. Based on the 2005–09 American Community Survey (ACS) it is estimated that almost 90,000 people of Afghan ancestry currently reside in the U.S. with the largest population residing in northern California, concentrated in Alameda County [2]. Alameda County is the site of ground-breaking studies on Afghan refugees where various health problems and insights into social and cultural stressors associated with being uprooted were uncovered for this population [3–4].

Research on the mental health of Afghans [5], and of many other refugee nationalities has shown that these populations are highly distressed [6] as a result of pre-migration traumatic experiences and post-resettlement factors such as social support loss, socio-economic and cultural adjustment difficulties, and difficulty with immigration and asylum processes [7–9]. While not a central focus of this line of research, in recent years several studies have investigated the impact of perceived discrimination on refugee health and mental health.

Perceived discrimination, defined as a type of stressor resulting from perceived assaults, fear, and exclusion experienced by racial and ethnic outgroups [10], has been shown to impact health through multiple pathways including reduced access to employment, housing and education leading to adverse emotional processes and associated psychopathology [11]. Additionally, stress models indicate that negative emotional states brought on by discriminatory experiences lead to structural and functional changes in multiple physiological systems [12]. Furthermore, it has been found that the perception of unfair treatment can harm an individual's self-esteem and self-efficacy or hinder opportunities to be active in social and economic spheres [13].

As far as we know, only three studies of Afghan refugees relate to this topic. In a qualitative study of recently resettled Afghans in Australia, discriminatory experiences exacerbated emotional responses to trauma (e.g. anger, fear, hopelessness, dispossession) [14]. And a study of primarily young adult 1^st^ and 2^nd^ generation Afghan-Americans [15] found perceived discrimination associated with depressive symptoms. More recently, in a cohort study of 2,399 humanitarian migrants in Australia, of which over one-quarter were from Afghanistan, discrimination was positively correlated with post-traumatic stress disorder (PTSD) and severe mental illness [16]. Similar patterns between the adverse effects of discrimination and mental disorders were observed among Somali youth in the U.S. [17] and young adult (predominantly) Iraqi refugees in Sweden [18]. Among North Korean refugees resettled in South Korea, perceived discrimination moderated the association between better socio-cultural adaptation and fewer depressive symptoms [19]. Similarly, based on their sample of young Middle Eastern refugees resettled in Denmark, Montgomery and Foldspang [20] found that discrimination, mental health problems, and social adaptation were mutually associated. The authors infer that acts of discrimination may influence refugees’ host country perceptions, resulting in the victim turning away from the assaulting group or host population, in turn, negatively affecting social adaptation.

These studies clearly indicate that the adverse effects of discrimination may deter more positive forms of acculturation, such as a preference for integration or a bi-cultural identity. Despite the many discrepancies in the conceptualization and measurement of acculturation [21], a preference for integration has been found to be most adaptive and associated with more positive mental health outcomes among Afghans [22] and other refugee groups [23]. In his review of immigration and social exclusion, Renzaho [24] draws on acculturation theory to suggest that a preference for integration may allow migrants to understand the strengths and weaknesses of their new environment whereas as separation and marginalization perpetuate perceptions of discrimination, ultimately having deleterious effects on health. This is further exemplified among immigrants in Europe and the Middle East where acculturation preferences that conflict with the host population were associated with either higher perceived discrimination or higher distress [25].

When considering coping responses to discrimination among refugees, important considerations include strength of ethnic identities and ties with families, coethnics, and members and organizations of the host society, all of which might buffer the negative health and mental health effects of discrimination. Yet little work on these topics has been done on refugees, who often have unique vulnerabilities (e.g. exposure to multiple traumas and life-threatening conditions) and modes of incorporation. Among Southeast Asian refugees in Canada, Noh et al. [26] as well as Beiser and Hou’s [27] discrimination studies found that stronger ethnic identity amplifies the risk of depressive symptoms, perhaps because discrimination is perceived as assaulting a core part of the self. Hence group identification may only be protective when it satisfies basic psychological needs of belonging and continuity for refugees, according to Çelebi et al. [28]. They test this hypothesis among Syrian refugees in Turkey and found that higher perceived ethnic discrimination was associated with lower mental and physical health, but not for those who derived a sense of control, distinctiveness and meaningfulness from their Syrian identity.

We did not find other research on the moderating effects of ethnic identities or ties on the influence of discrimination on mental health among refugees, but Mossakowski [29] shows that for Filipino Americans a strong ethnic identity reduced the influence of discrimination on depressive symptoms. However, a recent review of studies focusing on health effects of discrimination among ethnic minorities and immigrant groups found inconsistencies with relation to the buffering effects of ethnic identity and social support [30]. The authors suggest that these inconsistencies may be linked to methodological factors such as small samples, diverse measures, little control over extraneous variables, and scales that rely only on perceptions/recall of past (discriminatory) events.

Much remains to be learned about the distress-buffering effects of these coping resources and acculturation factors, especially for understudied Muslim refugee groups like those from Afghanistan. The present paper aims to extend knowledge in this area based on data collected between 2007–2008 from current and former Afghan refugees resettled in northern California. It is worth mentioning that in a previous publication, we used this dataset to examine gendered sources of distress [31]. Though here we examine the extent to which Afghans perceive experiencing discriminatory acts, how this affects their mental health, and whether the stress associated with perceived discrimination is moderated by the effects of pre-migration traumas and a number of post-resettlement factors. Informed by previous studies, we hypothesized that 1) higher reported perceived discrimination will be significantly associated with higher levels of psychological distress, after controlling for salient demographic characteristics; 2) post-resettlement adjustment factors (higher intra-ethnic identity, ethnic orientation, social support, and civic engagement) will be negatively associated with levels of distress and exert a buffering effect on the discrimination-distress pathway; whereas, 3) pre-resettlement trauma will be positively related with distress and amplify the relationship between discrimination and distress.

Aside from the importance of learning more about the effects of discrimination on refugee’s mental health, this study is significant because several factors may make Afghans more vulnerable to discrimination stress: 1) the rise in anti-foreigner prejudice and *xenophobic* discrimination toward immigrants and refugees in general [32]; relatedly, 2) their Muslim faith, made more visible through increased negative media attention after 9/11; and, 3) recent sociopolitical events, for example, the fact that the U.S. is militarily involved in Afghanistan, which likely influences negative views toward Afghans. Our tests for moderating effects could provide mental health professionals insight into developing interventions that build on protective/resiliency assets that mitigate the stress associated with discriminatory experiences.

## Materials & methods

### Sampling

Inclusion criteria for this study consisted of being of Afghan ancestry, an adult over the age of 18 years, and having resettled in the U.S. as a refugee. Participants were recruited in Alameda County, California between November 2007 and May 2008. Recruitment took place through various organizations or programs serving the local Afghan community, including mosques with large Afghan congregations, and health and social services organizations providing a range of services to Afghans (e.g. cultural preservation, and assistance navigating welfare, immigration, and medical bureaucracies). We purposively sampled organizations targeting gender, ethnic (e.g. Tajik, Pashtun), and year of arrival (1980s, 1990s, 2000s) figures from the American Community Survey. We initially selected Afghan-serving mosques because combined they are broadly representative of established Afghans, as even many less religious Afghans maintain memberships for burial purposes. In addition to working with Afghan-serving mosques, we selected an organization that served primarily people who had arrived in the 2000s, and when one mosque decided not to participate we added an organization whose members were primarily Pashtun to increase the representation of this major ethnic group. If the selected organization had a basis for developing a sampling frame we created the list and selected participants at random. Slightly over half of the sample was created this way. In organizations where a sampling frame was unavailable, we sampled purposively to achieve a representative gender and age balance. Because our sample was nonrandom, we monitored, but did not record precise response rates. However, because potential respondents were contacted first by trusted organizational leaders and ads featuring community leaders encouraging participation ran on a local Afghan television station, coordinators reported very high response rates. A comparison with Afghans in the 2005–09 American Community Survey suggests that our sample modestly over-represents unemployed women, and less educated and older (ages 60–69), first generation Afghans.

### Procedures

The survey of primarily fixed-choice questions was constructed by the second author aided by a team of Afghan informants and community leaders. After piloting an English version of the survey with Afghans fluent in English, two Dari translations and a Dari-in-English transliteration were produced independently by two bilingual Afghan educators and a graduate student. The second author, who was not fluent in Dari, then went through the questionnaire, item by item, with a team of three different bilingual informants (one 2nd generation with research training and two 1st generation, one with research training). For all items where there was not unanimity on all three translations, the second author led a discussion of possible wordings until there was mutual agreement on the wording that best reflected the intent of the original item and the semantic context of the broad range of first generation Afghans in Alameda County. In some instances, this led to novel wording not used in any of the three translations.

In addition, informants were asked to identify items that Afghan respondents would be reluctant to answer or would likely answer falsely. Where possible, the team altered wording or added explanation or assurances of confidentiality to increase the item’s validity. Several items were dropped from the survey when informants concluded edited versions would still not elicit valid responses. The Dari translation that resulted from this process was then use to revise the Dari-in-English version to exactly match the Dari version. The Dari-in-English version was used by those interviewers who were fluent Dari speakers, but not readers. This questionnaire was then pretested by ten interviewers, each doing two interviews of Dari speaking Afghans. Interviewers took notes on any problems with understanding and reluctance to answer sensitive questions. This led to minor wording edits and dropping several sensitive questions. We then pre-tested the survey, made final changes, and computerized they survey using CATI software to improve coding accuracy and reliability of skip sequences. The research, survey design, and signed consent process were approved by the Institutional Review Board at the second author’s university.

### Measures

#### Dependent variable

**Psychological Distress.** The dependent variable in our analyses is the *Talbieh Brief Distress Inventory* (TBDI) which was designed as a general measure of distress among immigrants [33]. The TBDI draws 11 items from the Psychiatric Epidemiology Research Interview Demoralization Scale (PERI-D) and 13 items from the Brief Symptom Inventory (BSI) that include items related to obsessiveness, hostility, sensitiveness, depression, anxiety, and paranoid ideation. TBDI was selected for brevity, breadth, and validity for this population in consultation with an Afghan American psychologist (PhD) familiar with the psychological problems faced by first generation Afghans in Alameda County. Items are based on a one-month recall period where respondents are asked to indicate the degree of discomfort caused by each item with response choices ranging from 0 = “not at all” to 4 = “extremely”. Scores range from 0–4 with higher scores indicative of higher severity of distress. For our sample, the TBDI demonstrated excellent internal reliability (Cronbach’s *α* = .960).

#### Primary independent variable

**Perceived Discrimination.** The primary independent variable is *perceived discrimination*, assessed using an equally weighted 4-item scale developed through preliminary qualitative interviews with members of the Afghan community residing in Alameda County area to identify and develop items with strong face validity that covered a broad range of experiences with discrimination. Two items are standard questions about personal experiences of discrimination and the other two items were developed inductively to capture perceived threats and unfair treatment after the events of 9/11. For example, we asked participants whether they were concerned that non-Afghans might harm them or their family members after 9/11; and, how often they were pulled out of line when going through security checks at airports for further questioning or having their luggage searched more thoroughly. The two standard items focused on perceptions unfair treatment when seeking employment and housing, and other public situations; and, whether respondents thought equally qualified Afghans have equal chances of getting jobs compared to other Americans. As illustrated in the results section, we coded item responses into three categories, ranging from 0 to 2, and scores are an equally weighted sum of these items ranging from 0 to 8, with higher scores indicative of higher perceived discrimination. Perceived discrimination has a Cronbach’s α of .588. While somewhat low, it is acceptable and expected because different dimensions of discrimination were measured. Our goal was to develop a measure with high face validity that reflected the breadth of Afghan’s experiences with discrimination. All four items were positively and significantly correlated with each other; the item-total scale correlations ranged from .330 to .423, and the Cronbach’s α if each of the items was deleted were all substantially lower than that of the full index. A Principal Component’s Analysis using a varimax rotation found one component with an Eigenvalue of 1.0 or higher, explaining 45.1% of the variance and the four items each loaded at .631 or higher.

#### Moderating variables: Pre- and Post-Resettlement factors

**Ethnic Orientation.** *Ethnic orientation* consists of four dummy variables constructed by comparing the scores on questions on strength of Afghan and American identities that had four response choices ranging from “not important” to “very important.” Adapting from Berry’s [34] acculturation typology, we operationalized ethnic orientation using the following categories: *assimilation* = American identity is stronger than Afghan identity (American identity minus Afghan identity > 0); *separation* = Afghan identity is stronger than American identity; *integration* = very important Afghan *and* very important American identities; *attenuated integration* (attenuation) = Afghan and American identities are equally strong, but neither are “very strong.” Berry’s fourth category is *marginalization*, but informants suggested that some Afghans who were best adapted to life in the U.S. *de-emphasized* both Afghan and American identities (thus ‘attenuated’).

**Intra-ethnic Identity.** *Intra-ethnic identity* is a binary variable based on questions on strength of identification with major ethnic groups in Afghanistan (Pashtun, Tajik, Hazara, Uzbek, Turkman). Respondents were coded as 1 (‘yes’) if they said *any* of their intra-ethnic identities were “very important” (the strongest rating of 4 choices). This creates binary variable of people who most strongly identify with ethnic identities and divisions which are highly salient in Afghanistan.

**Civic Engagement.** We used a summated index of four ‘yes/no’ items for measuring *civic engagement*. Items gauge 1) Is the respondent a U.S. citizen? 2) Did he/she vote in the 2006 election? 3) Did she/he volunteer in an organization serving Afghans in the past year? 4) Did she/he volunteer in another civic organization in the past year (not serving Afghans). A categorical Principal Component’s Analysis found two dimensions explaining 71.1% of the variance and produced a Cronbach’s α of .865. Volunteering in Afghan and non-Afghan organizations loaded strongly on the first component (.832 and .875 respectively), and citizenship and voting loaded heavily on the second component (.846 and .790 respectively). Despite its two-dimensional structure, combining these items is justified because each dimension captures an important aspect of civic engagement.

**Social Support.** *Social support* is a summated index of four yes/no items: Is the respondent married and living with his/her spouse? Is one or more other family members living in his/her household? Does he/she see at least 10 other family members (not in household) on at least a monthly basis (regular contact with extended family)? Does she/he have one or more close friends she/he can rely on if she/he needs help? A categorical Principal Component’s Analysis found two components with Eigenvalues over 1.0 explaining 56.9% of the variance and produced a Cronbach’s α of .747. Married, living with spouse and another family member living in the household loaded on the first component (.723 and .750 respectively), and having a close friend and extended family loaded on the second component (.776 and .723 respectively). Despite having two dimensions, combining these items is justified because both dimensions capture intimate ties that are likely to provide substantial emotional or practical support.

**Pre-resettlement Traumas.** We assessed *pre-resettlement traumas* using a summated index of 11 ‘yes/no’ items on war-related traumatic events that participants either experienced or witnessed while in Afghanistan and when fleeing. Items related to witnessing atrocities and experiencing displacement such as seeing someone being killed or maimed, the death or maiming of close friends and family members, threats to one’s own life, being imprisoned or taken hostage, being internally displaced, being displaced in a surrounding country, experiencing greatly impaired functioning, having resided in a refugee camp. A categorical Principal Components Analysis found two components with Eigenvalues over 1.0 that explain 41.9% of the variance and produced a Cronbach’s α of .861.

#### Control variables

We controlled for a number of salient variables shown to influence psychological distress in prior studies of Afghans. Control variables included age in years, gender, English language ability, education, year of arrival in the U.S., and employment status. *Education* is a four-category variable measuring the highest level of education (achieved in Afghanistan, the U.S., or some other country). The categories are less than high school; high school graduate or GED; some college, two-year Associate’s degree, or technical degree; and 4-year college degree or higher. *English Ability* is the mean score of 3 items on self-rated ability in speaking, writing, and reading English (0 = “Not at all” to 4 = “Very fluent”). Cronbach’s *α* for the English ability scale is .973.

### Data analysis

We used SPSS, version 24.0 [35] for all data analysis. We replaced missing items with mean scores for respondents who answered at least 21 of 24 TBDI items and 3 of 4 perceived discrimination items, dropping those without 21 of 24 or 3 or 4 respectively from the analysis. For other variables cases, were dropped for missing items; however, we imputed values for two of the 11 items for 48 cases in pre-resettlement traumas (missing due to interviewer or computer error) using predicted values from models that regressed the item scores on the remaining trauma items, age, gender, employment status, and education variables. We believe this solution for the missing pre-resettlement items only modestly affected the results because there was comparatively little variation in these two items. Almost seven in eight (87.1%) had experienced “life threatening situations,” while less than one in seven (13.9%) were “captured, imprisoned, or held hostage.” Pearson’s *r* between the 9-item index and the 11-item index is 0.987. When it made analytical sense, we treated “don’t know” responses as “no”, for example, two respondents who said they “don’t know” if they have a close friend they can rely on were treated as not having a close friend. The sample size for regressions ranged from 241 to 250 because of missing values.

**Fig 1:**
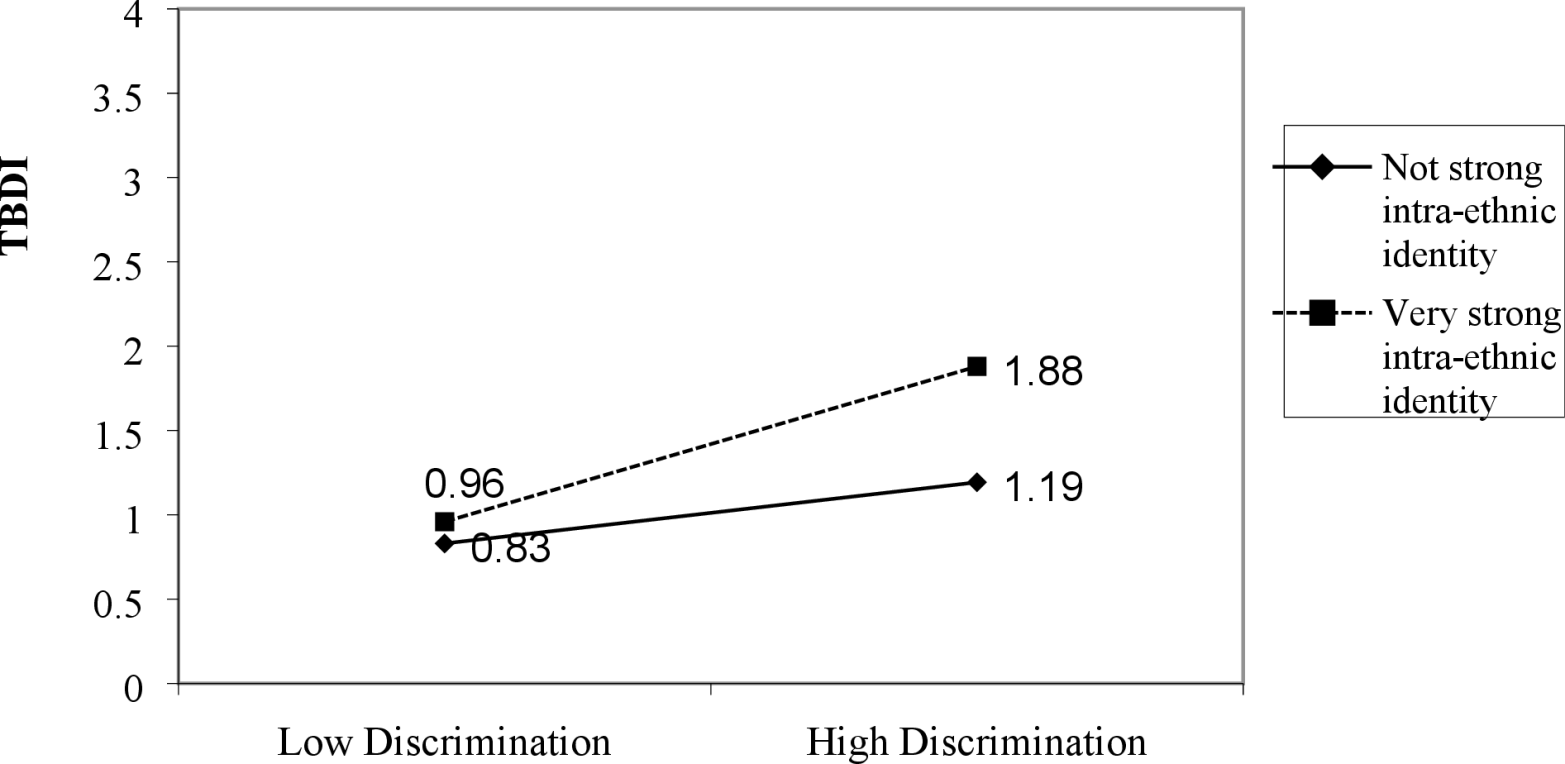
Discrimination by intra-ethnic identity explaining TBDI.

**Fig 2:**
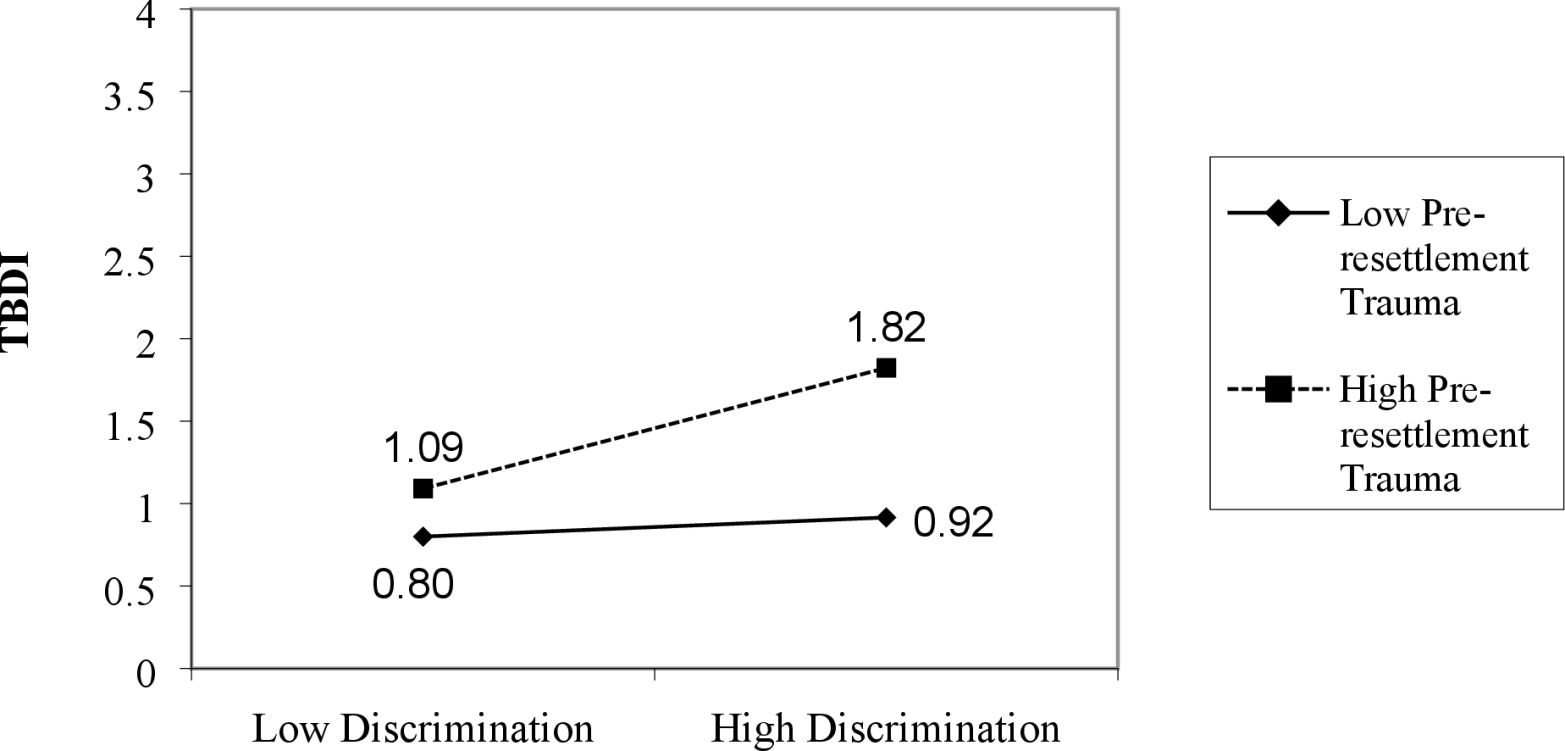
Discrimination by pre-resettlement trauma explaining TBDI.

Pearson, point-biserial, and Spearman’s correlations were used to examine relationships between continuous and categorical independent variables and the dependent variable (TBDI). To test our hypotheses, hierarchical regression analyses were used, consisting of six ordinary least squares (OLS) regression models explaining TBDI. In Model 1 we entered five socio-demographic variables as core controls; Model 2 adds discrimination to assess its independent influence; and Model 3 added our four key explanatory variables related to resettlement conditions. To Model 3, we then separately added interaction terms for each of the four resettlement factors by discrimination to test for moderators of discrimination. Non-significant moderators are reported in the text only. Model 4 reports results for the one significant (p < .05) resettlement moderator (intra-ethnic identity x discrimination). Model 5 adds pre-resettlement trauma to Model 3 to test for the effects of pre-resettlement trauma controlling for the controls and explanatory resettlement variables. Model 6 adds pre-resettlement trauma x discrimination to Model 5 to tests pre-resettlement trauma as a moderator of discrimination’s effect on distress. Significant interactions are plotted or graphed in Figs 1 and 2.

## Results

### Socio-demographic characteristics

Table 1 reports percentages for categorical variables and means, standard deviations and ranges for continuous variables. The far-right column reports Pearson, Spearman, or point-biserial correlations and significance levels with the dependent variable (TBDI). A sample of 259 Afghans participated in this study. Our sample was on average 48.75 (*SD* = 15.68) years of age and more likely to be female with a majority possessing a high school diploma or lower. A majority were unemployed, with moderate levels of English ability, and year arrived in the U.S. ranging from 1980 to 2008 (*M* = 1993, *SD* = 8.06).

**Table 1:** Descriptive statistics: Dependent, control, independent variables, and bivariate relationships with dependent variable (*N* = 259).

Variables		Statistics				
		*n* (%)	*M*	*SD*	*Range*	*Pearson ‘r’ or Point-Biserial ‘r’ with TBDI*
Talbieh Distress Inventory (TBDI)		251	1.208	0.993	0–4	—
Age in years		259	48.751	15.683	18–84	.131*
Gender						-.250***
	Male	126 (48.6)				
	Female ^a^	133 (51.4)				
Education			1.321	1.169	0–3	-.206**
	Less than high school	83 (32.0)				
	High school diploma	75 (29.0)				
	AA, some college, technical degree in U.S.	36 (13.9)				
	Bachelor’s Degree or Higher	65 (25.1)				
Employment status						
	Employed (or student)	107 (41.3)				-.273***
	Not Employed ^a^	152 (58.7)				
English ability		259	2.034	1.294	0–4	-.299***
Year Arrived in U.S.		257	1993.51	8.064	1980–2008	.101
Perceived Discrimination		247	.6808	.5554	0–2	.266***
Intra-ethnic identity						.300***
	Very strong	103 (39.8)				
	Not very strong^a^	156 (60.2)				
Ethnic orientation						
	Assimilation ^b^	20 (7.7)				.008
	Separation ^b^	117 (45.2)				.176**
	Integration ^b^	60 (23.2)				.028
	Attenuated integration ^b^	62 (23.9)				-.235***
Civic engagement		259	1.270	1.112	0–4	-.087
Social support		259	2.371	.957	0–4	-.161*
Pre-resettlement traumas		254	5.572	2.435	0–11	.493***

Notes.

^a^ = reference category.

^b^ = reference is the other 3 ethnic orientations.

* p < .05.

** p < .01.

*** p < .001.

### Descriptive analysis

As shown in Table 1, bivariate analyses indicate that with the exception of the ‘year of arrival’ variable, the other five controls (age, gender, education, employment, and English ability) have bivariate relationships with TBDI that are significant at *p* < .05 (two-tailed). Being male, more highly educated, employed, and possessing greater English competence are associated with lower levels of distress, while being older is associated with higher TBDI scores.

Average TBDI scores suggest that distress symptoms occur at moderately low levels. Apart from the control variables, we found that with the exception of ‘civic engagement,’ bivariate correlations between the moderator variables and TBDI are all significant at *p* < .05. An ‘attenuated integration’ ethnic orientation and greater social support were associated with lower distress levels, fitting our expectations. Contrary to our expectations, a strong intra-ethnic identity was positively associated with levels of distress. Confirming our expectations, a ‘separation’ ethnic orientation and pre-resettlement traumas were positively associated with levels of distress. Our primary independent variable, perceived discrimination was positively associated with TBDI scores. Table 2 shows that participants most often reported concerns about harm toward them or their family members from non-Afghans after 9/11 and least often when reporting incidents of direct discrimination at work, seeking or jobs or housing, in school, and in other public situations.

**Table 2:** Perceived discrimination items and percent responses.

Discrimination Items	%
After the tragic events of September 11, 2001, were you more concerned that non-Afghans might harm you or someone in your family?	
0 = No, not more concerned/Don’t know	41.3
1 = Yes, somewhat more concerned	18.9
2 = Yes, much more concerned	39.8
After September 11, 2001, when going through security checks at an airport, were you ever pulled out of the line to ask you extra questions or check you and your luggage more thoroughly?	
0 = No/Don’t know	51.5
1 = One or two times	33.2
2 = Three or more times	15.3
Discrimination is when someone harms another person simply because of the group he or she is part of. For example, if an employer does not hire an Afghan or a Muslim because the applicant is Afghan or Muslim, this is discrimination. Since living in the Bay Area, were you ever discriminated against because you are Afghan, when applying for a job or a promotion in your present job, seeking housing, in school, or some other way?	
0 = No/Don’t know	82.1
1 = Yes, pretty sure	3.2
2 = Yes, definitely	14.7
Do you think that equally qualified Afghans have equal chances of getting jobs compared to other Americans?	
0 = Yes	57.5
1 = Not sure	9.1
2 = No	32.8

### Multivariable analyses

#### Hypothesis 1: Effect of perceived discrimination on distress

Table 3 reports six OLS regressions that test our three hypotheses. Model 1 includes the five controls, which together explain 11.5% (adjusted R^2^) of the variance in distress levels (TBDI). Greater English ability and being male both lower distress levels and are significant at *p* < .05. No other controls are significantly associated with TBDI scores. Perceived discrimination is added in Model 2, and is positively associated with TBDI, explaining an additional 9.5% of the variance (*β* = .54, *p* < .001), and confirming our first hypothesis.

**Table 3:** Discrimination and other resettlement factors explaining psychological distress (TBDI).

	Model 1		Model 2		Model 3		Model 4		Model 5		Model 6	
	B (SE)	*β*	B (SE)	*β*	B (SE)	*β*	B (SE)	*β*	B (SE)	*β*	B (SE)	*β*
(Constant)	1.68 (.28)		1.28 (.27)		1.58 (.32)		1.84 (.33)		.625 (.349)		1.057 (.388)	
Age	.00 (.01)	.03	.00 (.00)	.03	.00 (.00)	.02	.00 (.00)	.01	.002 (.004)	.03	.002 (.004)	.03
Education	-.02 (.06)	-.03	-.01 (.05)	-.02	.05 (.06)	.06	.04 (.06)	.05	.054 (.054)	.07	.052 (.053)	.06
Employed	-.21 (.15)	-.11	-.18 (.15)	-.09	-.21 (.14)	-.11	-.23 (.14)	-.12	-.201 (.134)	-.10	-.176 (.133)	-.09
Gender (Male)^a^	-.32 (.14)	-.16*	-.35 (.13)	-.18**	-.39 (.12)	-.20**	-.41 (.12)	-.21***	-.320 (.116)	-.17**	-.345 (.115)	-.18**
English Ability	-.13 (.06)	-.18*	-.14 (.05)	-.20**	-.11 (.05)	-.15*	-.11 (.05)	-.15*	-.043 (.051)	-.06	-.040 (.051)	-.06
Discrimination			.54 (.10)	.31***	.56 (.10)	.32***	.33 (.13)	.19**	.445 (.099)	.26***	-.092 (.240)	-.05
Strong Intra-Ethnic Identity ^b^					.44 (.13)	.22***	.07 (.19)	.03	.329 (.121)	.17**	.300 (.120)	.15*
Integration ^c^					-.22 (.14)	-.10	-.21 (.14)	-.09	-.203 (.132)	-.09	-.229 (.131)	-.10
Attenuation ^c^					-.29 (.14)	-.13*	-.31 (.13)	-.14*	-.251 (.128)	-.11	-.278 (.127)	-.12*
Civic Engagement					-.03 (.06)	-.04	-.05 (.06)	-.06	-.033 (.053)	-.04	-.047 (.053)	-.05
Social Support					-.20 (.06)	-.19***	-.21 (.06)	-.21***	-.174 (.056)	-.17**	-.192 (.056)	-.19***
Intra-ethnic Identity x Discrimination							.51 (.19)	.28**				
Pre-resettlement Trauma									.119 (.023)	.30	.053 (.035)	.135
Pre-resettlement Trauma x Discrimination											.093 (.038)	.403*
*R*^*2*^	.133***		.212***		.315***		.334***		.384***		.400***	
Adjusted *R*^*2*^	.115***		.193***		.282***		.300***		.352***		.366***	
Δ*R*^*2*^			.095***		.102***		.020** ^d^		.069***		.016*	
*F-statistic* Δ *R*^*2*^			28.666		6.957		6.818^d^		27.561		8.421	

Notes

*p < .05.

** p < .01.

***p < .001.

References

^a^ = female.

^b^ = not strong intra-ethnic identity.

^c^ = assimilation and separation. Explained variance.

^d^ = Δ*R*^*2*^ from Model 3.

#### Hypothesis 2: Moderating effects of post-resettlement factors

Model 3 adds the four explanatory variables that reflect resettlement conditions, three of which are associated with distress at *p* < .05, explaining an additional 10.1% of the variance in TBDI. We addressed resettlement factors first in order to get a clear picture of current conditions associated with distress, prior to introducing the effects of our retrospective measure of pre-resettlement trauma. Based on standardized betas in Model 3, perceived discrimination is the most influential resettlement factor for explaining distress. Perceived discrimination’s beta increases slightly (.54 to .56) indicating no mediation of the discrimination-distress relationship by the other explanatory variables.

Moreover, Model 3 shows that, with controls, civic engagement has no significant effect on distress levels, contrary to our expectation. Also, having an ‘integration’ ethnic orientation, which we expected to reduce distress levels, is negatively, but not significantly associated with TBDI, whereas the ‘attenuated integration’ ethnic orientation significantly reduces distress levels at *p* < .05, confirming expectations based on informants’ observations. Also, as observed at the bivariate level, having a strong intra-ethnic identity is positively and significantly associated with distress (p < .001) and social support is associated with lower levels of distress at *p* < .001.

Model 3 was our base model for adding interaction terms to test the moderating influence of resettlement factors, and to test the influence of pre-resettlement trauma and pre-resettlement trauma x discrimination on levels of distress.

We tested for the moderating effects of these resettlement factors (civic engagement, ethnic orientation, intra-ethnic identity, and social support) by adding interaction terms (e.g. civic engagement x discrimination) one at a time to Model 3. Models testing civic engagement x discrimination, social support x discrimination, and all four of the ethnic orientation interactions were not significant at p < .05 and therefore these models are not shown. Regarding the ethnic orientation interactions, because ethnic orientation is a four-category dummy variable, we tried entering the interactions both one at a time and all at once, using ‘separation’ and then again using both ‘separation’ and ‘assimilation’ as the reference. None of the interactions were significant in any of these tests. While not significant at p < .05, the civic engagement x discrimination interaction approached significance (p = .079). Contrary to our expectations, discrimination increased predicted distress levels more for those with *high* levels of civic engagement than those with low levels of civic engagement. Thus, the observed pattern was that respondents with high civic engagement were *more* vulnerable to the effects of discrimination on levels of distress.

Model 4 in Table 3 shows that the intra-ethnic identity x discrimination interaction is significant at p < .01 (Δ R^2^ = 2.0%) and Fig 1 shows that possessing a strong intra-ethnic identity *increases* the influence of discrimination on levels of distress. Among those with “very strong” identifications with one of several ethnic groups within Afghanistan, going from low to high discrimination increases predicted TBDI by over 9/10 of a point, compared to under 4/10 of a point for those who do not have a very strong intra-ethnic identity. Thus, we found both higher distress levels and greater vulnerability to the negative influence of discrimination for those with strong intra-ethnic identities (e.g. Pashtun, Tajik).

#### Hypothesis 3: Moderating effect of pre-resettlement traumatic experiences

Model 5 (Table 3) adds pre-resettlement trauma to Model 3. It shows that pre-resettlement trauma is strongly and positively associated with distress, explaining an additional 6.9% of the variance in TBDI. This strongly confirms hypothesis 3a. Based on standardized betas pre-resettlement trauma is the most influential factor in Model 5 and perceived discrimination is second most influential. Model 6 adds the ‘pre-resettlement trauma x discrimination’ interaction term, showing that this interaction is statistically significant (p < .05; Δ R^2^ = 1.4%). This raises the total variance in TBDI explained by Model 6 to 36.6%.

Fig 2 plots the ‘pre-resettlement trauma x discrimination’ interaction. The influence of discrimination on distress levels is greater for those who have experienced more pre-resettlement traumas, confirming our third hypothesis. Moving from low to high discrimination raises predicted distress levels more for respondents who experienced high levels of pre-resettlement trauma (over 7/10 of a point on the TBDI scale) than those who experienced low pre-resettlement trauma (just over 1/10 of a point).

In Model 6, we see that gender is the only control variable significantly related to TBDI at p < .05. In addition, this model underlines the robust relationships social support, strong intra-ethnic identity, and ‘attenuation’ ethnic orientation have with TBDI. Note that perceived discrimination continues to be quite influential, but the main discrimination term is not significant. Thus, perceived discrimination’s association with TBDI is carried substantially through the discrimination x pre-resettlement trauma interaction term. This is reflected in the minimal influence discrimination has on TBDI for those with low pre-resettlement trauma.

We then ran a test that combined the two significant interactions (not shown) and found that while combined they explained an additional 2.0% of variance in TBDI (p < .05), neither interaction term was significant at p < .05. The pre-resettlement trauma x discrimination term was very close to significant (p = .05). The standardized beta for intra-ethnic identity x discrimination declined by 50% (.28 to .14) when adding pre-resettlement trauma and pre-resettlement trauma x discrimination, suggesting that intra-ethnic identity’s amplifying effect on the association between discrimination and distress may be intertwined with pre-resettlement trauma.

We ran the seven regressions in Table 3 not imputing missing data for the two items in pre-resettlement trauma, and not replacing with means for missing data for respondents answering 3 of 4 discrimination items and 21 to 23 of 24 TBDI items. This substantially reduced the sample size, dropping it to 179 in the full model. In addition, we reran model 6 another time, substituting a 9-item measure of pre-settlement trauma that drops the two items with high missing values the for the 11-item measure. The patterns of significant relationships remained the same with a few exceptions. Overall, our core findings and interpretations remain virtually the same. The standardized beta for perceived discrimination increased modestly for Model 2 (from .31 to .35), as did the r^2^_change_ (from 9.5% to 12.0%). Thus, by replacing missing values we may be modestly underestimating the positive influence of perceived discrimination on levels of distress. In addition, in Model 3 the influence of an “integration” ethnic orientation went from non-significant to marginally significant (p < .10) and the influence of an “attenuation” ethnic orientation went from significant at p < .05 to non-significant. This pattern continued in subsequent models and in the final Model 6, integration was significant at p < .05 while attenuation was non-significant. While this difference is noteworthy, it does not change our main interpretation about ethnic orientation, that strong intra-ethnic identity has substantially more influence than traditional bi-dimensional ethnic orientations for this population. Strong intra-ethnic identity remains positively and significantly associated with distress in the no replacement models. (When we removed strong intra-ethnic identity from the analysis, attenuation, but not integration, was robustly significant throughout.) The most important differences come in Models 5 and 6. Using the non-imputed 11-item pre-resettlement trauma measure, r^2^_change_ in Model 6 is 9.5%, compared to 6.9% reported in Model 5. This indicates that we may be underestimating the positive influence of pre-resettlement trauma on current levels of distress. For Model 4, the influence of the strong intra-ethnic identity x discrimination term grew in both non-replacement models. For the model that included both strong intra-ethnic identity x discrimination and pre-resettlement trauma x discrimination (civic engagement x discrimination continued to drop out), using the 11-item measure of pre-resettlement trauma, R^2^_change_ (3.0%) was significant at p < .01, but neither interaction term was significant at p < .05. Adding the interaction terms continued to find both significant at p < .05. Substituting in the 9-item pre-resettlement trauma measure found that when entering both interaction terms the r^2^_change_ was 3.2% (p < .01) and both interaction terms were significant at p < .05. In summary, our reported Model 4 may be underestimating the influence of intra-ethnic identity x discrimination and our additional tests suggest the influence of strong intra-ethnic identity x discrimination may be as great as pre-resettlement trauma x discrimination. Overall, our interpretations based on the no replacement models remain the same except for modestly upgrading the influence of the strong intra-ethnic identity x discrimination interaction.

## Discussion

This cross-sectional study examined the effect of perceived discrimination on the mental health of current and former Afghan refugees residing in northern California. Overall, we found that both psychological distress and perceived discriminatory experiences occurred at low to moderate rates. However, discrimination did show a significant positive association with psychological distress, which confirms our first hypothesis. This finding fits with our recent study of 1^st^ and 2^nd^-generation Afghan-Americans examining the effect of discrimination on mental health[15] and Chen et al.’s [16] study of a diverse group of humanitarian migrants in Australia, some originating from Afghanistan. Our findings are also comparable to several studies of other refugee groups resettled in industrialized nations using different measures of discrimination [17–20].

Our measure of discrimination captured the degree of unfair treatment and marginalization that Afghans perceive, which is concerning given that such acts may further hinder opportunities in social and economic spheres, already known to play a significant role in determining the mental health of Afghan refugees [5]. The predicament that Afghans find themselves in may indeed be exacerbated by host society resentment over their presence, as exemplified by their perceived fears of hate crimes targeting Muslim communities after 9/11, the most prevalent form of perceived discrimination we measured. The occurrence of this type of discrimination has been documented among refugees of Arab-Muslim descent in the U.S. by Kira et al. [36] who suggests that such discriminatory acts, and the fears they foster, hinder adjustment and integration in a new host society.

Turning to our second set of hypotheses, we found that factors such as civic engagement moderated the effects of discrimination on TBDI; however, this relationship occurred in the opposite direction of our prediction. Rather, the negative mental health effects of discrimination are *greater* among those who are more civically engaged. It is possible that civic engagement may create opportunities to be the target of *unmeasured* discrimination. Although, this pattern fell short of statistical significance (p = .079) and cross-sectional data cannot confirm causal direction, future research should test this more carefully. It may be that discrimination targeting refugees is part of a mechanism that limits civic involvement by increasing distress levels among the civically engaged. In effect, avoiding civic and political engagement becomes a coping strategy in the context of widespread Islamophobia. If future research confirms this pathway, it may be that Afghans face a dilemma, where seemingly positive acculturation-related behaviors, result in increased levels of distress.

Similarly, we unexpectedly found higher distress levels and greater vulnerability to the negative influence of discrimination for those with strong intra-ethnic identities. Previous research has shown that strong in-group identification is protective when it provides psychological benefits, as in the case of Syrian refugees [28] and Filipino immigrants [29]. Others have argued that strong ethnic identity (likely found in strongly bonded communities) sometimes increases the negative influence of discrimination [27], because discrimination is perceived as assaulting a core part of the self. This latter interpretation fits our findings. We found that strong intra-ethnic identity amplifies the distress-discrimination pathway. It is possible that strong intra-ethnic identities reflect strong ties with Afghans in Afghanistan and involvement with the ongoing strife in Afghanistan. Such identifications might both reflect social networks, which, as mentioned above, may emphasize loss and vulnerability, given conditions in Afghanistan, and contribute to a strategy of forbearance that minimizes the impact of discrimination faced by Afghans in the U.S. Therefore, it is questionable whether strong ethnic ties in general are helpful in combating the stress associated with discrimination, when they may be a marker for increased marginalization and/or ties to co-ethnics whose life conditions make struggles in the host society pale by comparison. This finding demonstrates the importance of research considering, when relevant, the influence of salient ethnic identities based on divisions within refugees’ country of origin.

Moreover, while our study confirmed the expectation that social support is negatively associated with distress, social support did not buffer the effects of discrimination as we expected. While not significant (p = .12) a plot of this relationship showed that the effects were the opposite of what we expected. Discrimination had a greater effect on distress levels for those with high social support (TBDI increased .9 points from low to high discrimination) than those with low social support (TBDI increased .4 points from low to high discrimination). This pattern aligns with results from several tests of other ethnic minority groups in the U.S. [30], and our recent study of Afghan-Americans [17] where we infer from our data that social networks may emphasize loss and vulnerability because they likely promote *individual* coping and striving to succeed in socio-economic spheres more than *collective* challenges to discriminatory experiences that might better validate and support those experiencing discrimination. A relevant example of an attitude of diminishing concerns about discrimination was a male in his early 40s who, in a focus group of mostly young adult Afghan males, disparaged Bay Area Afghans who complain about discrimination or difficulties getting established, emphasizing their privileged position in the Afghan diaspora and comparing them with a tone of ridicule to Afghans living precariously in Afghanistan.

It may be that Afghans likely have relatively undeveloped or marginalized cultural repertoires of challenging discrimination in comparison to other stigmatized groups. For example, Lamont shows that African-Americans have widely available scripts of response that buffer the effects of discrimination by promoting narratives of “we-ness,” a shared tradition of resilience in the context of continued discrimination, which helps individuals make sense of their experience, and an identity defined in opposition to that of discriminators [37]. Cultural repertoires which emphasize the privileged position of U.S. Afghans, minimize experiences with discrimination, and counsel individual effort [38], may limit the development of similar buffering scripts for Afghans. Future research is needed to explore the coping styles and cultural scripts Afghans use, to better understand existing and potential sources of resilience against discriminatory experiences for this, and other Muslim refugee groups.

In sum, our data does not support our second hypothesis asserting that intra-ethnic identity, integration, civic engagement, and social support would buffer the effects of discrimination on mental health. This warrants further empirical attention to identify helpful social resources within Afghan refugee communities needed to better confront, reduce and protect against discrimination [39–41]. However, our data does indeed support our third and final hypothesis confirming that perceived discrimination has a stronger mental health impact on individuals with higher pre-resettlement traumatic experiences in our sample. This interplay of variables is understudied in previous research, but speaks directly to an important vulnerability of this population. It may be that the vulnerability caused by previous traumas and stressors intensifies the negative emotional effects of experiencing discrimination or feeling targeted, or previous trauma decreases one’s ability to cope with such assaults. An implication of this, suggested by Kim and Noh [41], is that mental health providers serving migrants who have experienced traumas should be attuned to potential adverse effects of discrimination and marginalization, as they may be an important cause for ongoing distress among refugees who have experienced significant trauma, especially those of Muslim backgrounds. Future research is underway by the authors of this study to examine a wider range of types of discrimination, stigmatization, and ‘assaults on the self’ experienced by Afghans; the strength and meanings of various ethnic and religious identifications; and the range of culturally supported responses to discrimination and stigma they engage in, how these influence well-being and perceptions of being marginalized, and influence patterns of political and civic engagement and pathways to radicalization among young Afghans in particular. Our findings here are suggestive, but we believe that complex social ecological models and measures of discrimination, ethnic and religious identity, cultural resources, acculturation processes, and mode of incorporation are needed to more fully register the influence of discrimination and stigmatization on Muslim refugees, and better understand individual and social sources of resilience that foster integration and buffer against discrimination and stigmatization.

There are limitations to this study. First, the cross-sectional design does not allow for causal inferences; therefore, findings reported here should be interpreted with caution. Secondly, when compared to 2005–2009 ACS sample, our sample underrepresented those with higher education levels and Afghans who arrived in the U.S. before age 13 (1.5-generation), while it overrepresented individuals 60–69 years of age and those relying on government assistance for basic needs. A major strength of this study is our inductive approach to selecting standardized measures (e.g. TBDI) and constructing other measures via qualitative analysis (e.g. perceived discrimination) by drawing on insights from affected Afghans and community leaders, possibly providing a higher degree of accuracy than measures developed and validated for other (non-refugee) populations. Future work must replicate the reliability of TBDI on this and other Afghan American populations and compare TBDI to other measures of distress utilized on diasporic Afghans [42]. Future work on perceived discrimination in this population should utilize and modify richer measures of perceived discrimination, and that of ethnic orientation by adapting well-validated multi-dimensional acculturation (Vancouver Index of Acculturation) and social integration scales [43]. Finally, regarding the amplifying effect of pre-resettlement trauma on the influence of discrimination on distress, future work should focus on whether the vulnerability caused by previous traumas and stressors intensifies the negative emotional effects of experiencing discrimination or feeling targeted, or previous trauma decreases one’s ability to use effective coping strategies in response to such assaults.

### Conclusions

Our approach has led us to conclude that perceived discrimination does indeed pose a significant threat to the mental health of current and former Afghan refugees. Discrimination may further exacerbate other ongoing post-resettlement stressors, while posing even more harm to traumatized Afghans. Because factors related to integration, group identification, and social support demonstrated little to no positive effect on buffering the discrimination-distress pathway, Edge and Newbold [43] suggest deeper insights into such moderation effects could be drawn through longitudinal analyses. Future research is warranted to uncover how Afghans cope with this type of stress, and to identify resiliency assets within communities that could inform interventions with this population.

## Supporting information

S1 File Survey Dari translation. (DOC)

S2 File Survey English version. (DOC)
