# Supplementary material for: Discrimination and distress among Afghan refugees in northern California: The moderating role of pre- and post-migration factors
Source: PLoS One. 2018 May 21;13(5):e0196822. doi: 10.1371/journal.pone.0196822 (PMC5962064; doi:10.1371/journal.pone.0196822)
Supplement: S1 File — (DOC) [file pone.0196822.s001.doc]

______

**سروی تشخیص احتیاجات اجتماع افغانها در المیدا کونتی- ایالت کل یفورنیا**

السلام علیکم. نام من ___________________________________________ است.

من عضو یک گروه تحقیق در یونیورسیتی ایالتی کلیفورنیا –ایست بی می باشم که در مورد تشخیص احتیاجات افغانها در المیدا کونتی مطالعه می کنیم. نتایج این مطالعات برای انکشاف و توسعه برنامه های که بتواند افغانها را بهتر کمک کند مفید خواهد بود. سهمگیری شما درین زمینه نهایت مهم است زیرا نمایندگی از زندگی افغانها در المیدا کونتی را می نماید.

سهمگیری شما درین مطالعات شامل جواب دادن سوالات در مورد زندگی شما در افغانستان و امریکا ، مسایل خانوادگی ، اشتغالات فرهنگی ، مکتب اطفال ، احتیاجات صحی و نظریات شما در باره برنامه های که بتواند افغانهای را که زندگی خود را درین کشور عیار میسازند و سر و سامان میدهند، کمک کند. این سروی در حدود شصت تا نود دقیقه را در بر میگیرد.

جوابات شما مطلقأ محرمانه خواهد بود. این بدین معنی است که نام شما با سوالات که جواب میدهید وابسته نخواهد بود. محقیقین و مصاحبه کنندگان به هیچ صورت جوابات شما را به هیچ کس افشا نخواهد کرد که این شامل موسسات دولتی مانند خدمات اجتماعی یا آی-ان-اس (INS) میباشد. جوابات شما در کنار جوابات دیگر افغانها فقط برای یک مطالعه علمی است که معلومات بهتر جمع آوری شود تا افغانها بطور عموم خوبتر درین دیار کمک شوند و احتیاجات شان مرفوع گردد.

همه ما درین تحقیق از وقت شما و کمک شما تشکر و قدر دانی می کنیم که در سروی بسیار مهم احتیاجات افغانها سهم می گیرید.

1. تاریخ امروز_________________
2. محل مصاحبه: 1. در خانه 2. در مرکز سروی 3. از طریق انترنت 4. جای یا طریق دیگر _______
3. شماره کود مصاحبه کننده: _________
4. جنس مصاحبه کننده: 1. مرد 2. زن
5. سن مصاحبه کننده: __________________
6. قومیت مصاحبه شونده: الف. هزاره ب. پشتون ج. تاجیک د. ترکمن ه. ازبک

ز. کدام قومیت دیگر_______________

1. زبان که مصاحبه به پیش برده شد ( دور یکی را خط بکشید)

الف. انگلیسی ب. دری ج. پشتو ه. کدام زبان دیگر ____________

1. چند نفر دیگر در وقت مصاحبه همرای مصاحبه شونده بودند )اگر کسی بوده باشد(؟___________

اگر نبوده باشد، از سوال بعدی صرف نظر گردد.

1. ارتباط این اشخاص با مصاحبه شونده چه می باشد؟
2. شخص اول همرای مصاحبه شونده:____________________________
3. شخص دوم همرای مصاحبه شونده: ____________________________
4. شخص سوم همرای مصاحبه شونده: ____________________________

1. چگونه مصاحبه شونده برای تحقیق نمونه گرفته شد. اول. مرکز تحقیق دوم. مسجد

سوم. انترنت چهارم. طریق دیگر____________

سوابق و مشخصات مصاحبه شونده:

1. جنس مصاحبه شونده: 1. مرد 2. زن 3. جنس دیگری
2. در کدام سال تولد شده اید؟ ____________

[اگر مصاحبه شونده سن خود را نمیداند و یا آرزو ندارد سن خود را افشا کند ، بطور تخمینی سوال کنید که چند ساله باشد]

1. در کدام شهر اقامت می کنید؟ _______________
2. در کدام کشور تولد شده اید؟
   1. افغانستان [دو سوال بعدی را بگذرید]
   2. امریکا
   3. پاکستان [سوال بعدی را بگذرید]
   4. یا کشور دیگر_____________
3. آیا در بی ایریا تولد شده اید؟
   1. بلی
   2. نخیر
4. آیا هرگز در افغانستان زندگی کرده اید؟ ا. بلی. 2. نخیر
5. کی در خانواده شما (برای شما) تصمیم گرفت که افغانستان را ترک کنید؟ خود شما یا همسر تان ، والدین شما ، فرزندان تان و یا کی؟

[بپرسید "برای شما" اگر مصاحبه شونده افغانستان را ترک کرده باشد. اگر مصاحبه شونده در افغانستان زندگی نکرده باشد "برای شما" را کنار بگذارید].

- 1. خودم
  2. همسرم
  3. خودم و همسرم
  4. والدین
  5. پدر کلان و مادر کلان
  6. فرزندان
  7. کسی دیگر ________________

1. در کدام سال شما / خانواده شما افغنستان را ترک کردید؟ بعضی مردم افغانستان را چند بار ترک کردند به خاطریکه دوباره برگشت کردند. اگر شما / فامیل شما اضافه تر از یک بار افغانستان را ترک کردید لطفأ جواب دهید که آخرین بار افغانستان را در کدام سال ترک کردید. [بپرسید "فامیل شما" اگر مصاحبه شونده هرگز در افغانستان زندگی نکرده باشد] ______________________________
2. دلیل عمده چه بود که شما / خانواده شما تصمیم گرفتید تا افغانستان را ترک گوئید؟ [بپرسید "فامیل شما" اگر مصاحبه شونده هرگز در افغانستان زندگی نکرده باشد] ___________________________

__________________________________________________________________________________________________________________________________

1. الف. در کدام منطقه افغانستان شما / خانواده شما زندگی میکردید قبل ازینکه کشور را ترک گوئید؟ [اگر مصاحبه شونده در جواب خود اضافه تر ازیک منطقه را ذکر کند بپرسید:

ب. در پینج سال اخیر پیش از بر آمدن از افغانستان در کجا زندگی میکردید؟ دور جواب سوال را خط بکشید] ____________________________________________________________

__________________________________________________________________________________________________________________________________

1. آیا شما/ خانواده شما در جای دیگر زندگی کردید بعد ازینکه از افغانستان خارج شدید و قبل ازینکه به بی اریا برسید؟
   1. نخیر ( سوال بعدی را بگزرید)
   2. بلی
2. لطفأ فهرست شهر ها و کشور های را که بعد از برآمدن از افغانستان زندگی کرده اید و پیش ازینکه به بی اریا برسید به تر تیب از اول تا آخر نام ببرید. ]پرسان کنید که چقدر مدت در هر شهر زندگی کرده اند طور مثال "نیویارک - 3 ماه"] _________________________________________ __________________________________________________________________________________________________________________________________
3. در کدام سال شما/ خانواده تان به امریکا رسیدید؟ ___________
4. [اگر در بی اریا تولد شده باشد سوال بعدی را بگذرید] در کدام سال شما به بی اریا رسیدید؟ ________
5. به کدام شهر بی اریا وقتیکه بار اول رسیدید زندگی داشتید و یا تولد شده اید؟ [شهر ها را به ترتیب از اولین شهر که زندگی کرده تا آخرین فهرست دهید. اگر کود ندارد نام آنرا ذکر کنید.]
   1. المیدا
   2. برکلی
   3. کسترو ولی
   4. کانکورد
   5. دبلین
   6. فریمانت
   7. هیوارد
   8. لیورمور
   9. میلپیتس
   10. نوآرک
   11. اوکلند
   12. پتسبرگ
   13. پلزنت هل
   14. پلزنتن
   15. سن هوزه
   16. سان فرانسیسکو
   17. سن لیندرو
   18. سن لورنزو
   19. سن رمون
   20. تیریسی
   21. یونین سیتی
   22. والنت کریک
   23. شهر دیگر________
6. به افغانستان برای کار و یا دیدار از سال 2001 به اینسو برگشت کرده اید؟
   1. بلی
   2. نخیر
7. آرزو دارید که یک روز برگشت کنید و در افغانستان زندگی کنید؟ ["زندگی کردن" به این معنی که بصورت دایمی جا گزین شوید].
   1. نخیر[از سوال بعدی صرف نظر گردد]
   2. بلی
8. آیا میخواهید سال آینده به افغانستان برگشت کنید و در آنجا زندگی کنید؟
   1. نخیر
   2. بلی
9. به غیر از امریکا به کدام کشورها اعضأی فامیل دارید که با آنها در تماس هستید؟
10. آسترالیا
11. افغانستان
12. آلمان
13. انگلستان
14. ایران
15. پاکستان
16. تاجکستان
17. ترکیه
18. دنمارک
19. روسیه
20. سویدن
21. عربستان
22. فرانسه
23. فنلیند
24. مصر
25. ناروی
26. هالند
27. کانادا
28. یونان
29. کشور دیگر1________________
30. کشور دیگر2________________
31. کشور دیگر3________________
32. بدون از بی اریا ، به کدام شهر ها و ایالات امریکا اعضای خانوداده دارید که شما به آنها در تماس هستید؟ [لطفأ فهرست شهرها"شهر های که بسیار شناسا نیستند" با نام ایالت تذکر دهید]. _________

__________________________________________________________________________________________________________________________________

1. در پنج سال اخیر چند بار دوستان و اقارب را که در کشورهای خارج امریکا زندگی میکنند ملاقات کرده اید ؟
   1. در پنج سال اخیر هیچ نی
   2. یک بار در پنج سال اخیر
   3. دو تا سه بار
   4. چهار تا پنج بار.
   5. شش تا ده بار
   6. زیاد تر از ده بار
2. قومیت خود را چگونه مشخص میکنید؟ در این لست است یا ملیت دیگری است میتوانید زیاد تر از یکی را نام ببرید.
   1. هزاره
   2. پشتون
3. تاجیک
4. ازبک
5. ترکمن
6. دیگر _____________________

] از هر ملیت که نام میبرد سوال بعدی را بپرسید]

به چه اندازه قوم (نام قوم) برایت مهم است ؟ مهم نیست،تا اندازهء مهم است،مهم است ویا بسیار مهم است برای شما؟ [نمبر جواب دهنده در مورد اهمیت ملیت اش در خالیگاه بعد از شناخت ملیت بنوسید. مثلآ، هزاره اهمیت _______________ ]

1. مهم نیست
2. تا اندازه ئی مهم است
3. مهم است
4. بسیار مهم است
5. هویت امریکائی شما به چه اندازه برای شما اهمیت دارد؟
6. مهم نیست
7. تا اندازه ئی مهم است
8. مهم است
9. بسیار مهم است
10. هویت افغانی شما به چه اندازه برای شما مهم است؟
11. مهم نیست
12. تا اندازه ئی مهم است
13. مهم است
14. بسیار مهم است

درین قسمت من در مورد مهارت شما در زبان های دری ، پشتو و انگلیسی پرسان می کنم.

| زبان | 1. سخن گفتن | 1. خواندن | 1. نوشتن |
| --- | --- | --- | --- |

| 1. دری/فارسی | 1. بسیار فصیح 2. متوسط 3. خوب 4. کم 5. قطعأ یاد ندارم | 1. بسیار فصیح 2. متوسط 3. خوب 4. کم 5. قطعأ یاد ندارم | 1. بسیار فصیح 2. متوسط 3. خوب 4. کم 5. قطعأ یاد ندارم |
| --- | --- | --- | --- |
| 1. پشتو | 1. بسیار فصیح 2. متوسط 3. خوب 4. کم 5. قطعأ یاد ندارم | 1. بسیار فصیح 2. متوسط 3. خوب 4. کم 5. قطعأ یاد ندارم | 1. بسیار فصیح 2. متوسط 3. خوب 4. کم 5. قطعأ یاد ندارم |
| 1. انگلیسی | 1. بسیار فصیح 2. متوسط 3. خوب 4. کم 5. قطعأ یاد ندارم | 1. بسیار فصیح 2. متوسط 3. خوب 4. کم 5. قطعأ یاد ندارم | 1. بسیار فصیح 2. متوسط 3. خوب 4. کم 5. قطعأ یاد ندارم |

1. آیا شما فعلا تابعیت امریکا را دارید، مقیم دایمی می باشید ، ویزای کار را دارید ، ویزای تحصیلی دارید یا کدام نوع ویزای دیگر دارید و اقامت شما شکل دیگر دارد؟
   - 1. تابعیت امریکا
     2. مقیم دایمی
     3. ویزه کار
     4. ویزای تحصیل
     5. ویزای دیگر___________________________
     6. نوع دیگر_____________________________

ازدواج و روابط زناشوهری

1. حالت معدنی فعلی شما چیست؟
2. متاهل
3. بیوه
4. طلاق شده (سوال بعدی صرف نظر گردد)
5. جدا شده به خاطریکه شما و همسر تان جور نمی آیید
6. جدا شده به خاطرشما یا همسرتان مجبور است در یک شهریا کشور دیگر زندگی کند
7. هیچ ازدواج نکرده ام ( شش سوال بعدی را تیر شوید)
8. حالت دیگر____________________________________
9. هیچوقت طلاق شده اید؟
10. بلی
11. نخیر ( از دو سوال بعدی تیر شوید)
12. چند بار طلاق شده اید؟___________
13. اگر طلاق شده اید ، کی تصمیم گرفت که به ازدواج اخیر شما خاتمه دهد؟
14. همسر سابقه ام
15. خودم
16. ما هر دو تصمیم گرفتیم که به ازدواج خود خاتمه دهیم
17. چند ساله بودید که بار اول ازدواج کردید؟__________
18. چند بار شما ازدواج کرده اید؟________
19. [پرسان کنید اگر جواب دهنده زن شوهر دار است] داشتن چند خانم در افغانستان یک موضوع قبول شده است. آیا شوهر شما زیاد تر از یک زن دارد؟ به یاد داشته باشید که جواب شما مطلقأ محرمانه پاسداری خواهد شد.
20. نخیر
21. بلی، او دو زن دارد.
22. بلی، او سه زن دارد.
23. بلی او چهار زن دارد.
24. بلی او پینج زن یا زیادتر دارد.
25. [پرسان کنید در صورتیکه مصاحبه شونده ازدواج کرده باشد] آیا ازدواج شما را والدین شما یا کسی دیگر از اعضای خانواده سر براه کرده است یا شما خود تان همسرتان را انتخاب کرده اید؟
26. پدر و مادرم / خانواده ازدواج مرا سر براه کردند.
27. پدر و مادرم / خانواده ام ازدواج مرا سرشته کردند و اما من خودم در مورد تصمیم گرفتم .
28. من همسرم را خودم انتخاب کرده ام اما بعدأ پدر و ماردم آنرا سر براه کردند.
29. من خودم همسرم را انتخاب کردم.
30. افغانهای که در امریکا زندگی میکنند به طرز ازدواج درین کشور روبرو هستند که بعضی از والدین در انتخاب همسر فرزندان شان نقش کمتر را بازی میکنند نسبت به افغانستان. آیا در خانواده شما معامله با دو طرز ازدواج یعنی یکی که والدین تصمیم میگیرند و دیگر که خود جوانان تصمیم میگیرند کدام اختلاف را به بار نیآورده است؟ هیچ اختلاف نشده، یک کمی اختلاف را باعث شده ، اختلاف بشکل معتدل آن بوده و یا بسیار شدید اختلاف وجدایی را باعث شده است؟
31. هیچ نی
32. یک کمی اختلاف شده
33. به شکل معتدل اختلاف را به بار آورده
34. به شکل وسیع اختلاف رخ داده است
35. به نظر شما، داشتن یک ازدواج سالم برای دانستن یک دیگر خوبتر است که طرفین احساس و پروبلم های خود را مطرح کنند؟. آیا گفته میتوانید که بسیار مهم است، تا اندازه مهم است و یا مهم نیست؟
36. بسیار مهم است
37. تا اندازه مهم است.
38. مهم نیست.
39. به چه اندازه مهم است که یک زوج توقعات یک خانواده دامنه ئی ( درین نوع خانواده مادر شوهر ، برادر شوهر ، پدر شوهر ، خواهر شوهر ، خاله ها عمه ها کاکا/ماما ها و برادر زن یک نقش دارد) را برای اینکه شوهر خوب، زن یا مادر خوب و پدر خوب باشد برآورده سازد ؟
40. بسیار مهم است
41. تا اندازه ئی مهم است
42. مهم نیست
43. [ پرسان کنید اگر در حال حاضر متاهل است ] بطور عموم میتوانید بگوید که ازدواج شما بسیار رضایتبخش است ، تا اندازه ئی رضایتبخش است و یا رضایتبخش نیست؟
44. بسیار رضایتبخش است
45. تا اندازه ئی رضایتبخش است
46. بسیار رضایتبخش نیست
47. فکر میکنید که یک عده زیاد افغانها در امریکا درین وقت ها طلاق میگیرند؟
    1. نخیر(دو سوال بعدی تیر شوید)
    2. بلی
48. به نظر شما ، یک دلیل عمده برای طلاق زیاد اینست که نظم خانواده ئی دامنه ئی در بین افغانها موقیعیت آن در امریکا ضعیف شده است؟

بلی

نخیر

نمی دانم

1. شما فکر می کنید که یک دلیل مهم برای طلاق زیاد در بین افغانها این است که زنان حقوق بیشتر در امریکا دارند؟
2. بلی
3. نخیر
4. نمی دانم
5. شما فکر میکنید که فراهم ساختن زمینه برای مشورت های خانوادگی یا تعلیم و تربیه امور ازدواج قبل از ازدواج بتواند جوانان را که نو ازدواج میکنند یک ازدواج سالم نصیب کند. اگر جواب بلی باشد ، بسیار کمک خواهد کرد و یا فقط تا اندازه ئی؟
6. بلی ، بسیار کمک خواهد کرد
7. تا اندازه ئی کمک خواهد کرد
8. نمی تواند کمک کند
9. متیقین نیستم
10. فکر می کنید که فراهم ساختن زمینه برای مشوره ازدواج که به اساسات فرهنگ افغانستان باشد بتواند یک ازدواج سالم را پاسداری کند؟ اگر جواب بلی باشد ، بسیار کمک خواهد کرد و یا فقط تا اندازه ئی؟
11. بلی ، بسیار کمک خواهد کرد
12. تا اندازه ئی کمک خواهد کرد
13. نمی تواند کمک کند
14. متیقین نیستم
15. الف. اگر یک زوج مشکلات شدید داشته باشند ، کی بهترین انتخاب خواهد بود که از او کمک گرفته شود؟

_______________________________________________________________

[اگر ضرورت می افتد ، طور مثال بهتر خواهد بود که از والدین کمک گرفته شود خواهر و برادر و یا دیگر اعضای خانواده ، دوستان ، سرشناسان کمونیتی ، مشاورین مسلکی ازدواج و یا امامان دینی. ب.____ این جا ص صیحیح کنید اگر یکی را انتخاب می کنید]

1. چه نوع روش ازدواج را شما ترجیح میدهید. روش که در جمله اول شرح شده و یا جمله دوم؟
2. یک ازدواج که مرد نفقه آورنده باشد و مسئولیت مالی خانواده را به عهده گیرد و زن مسئولیت تنها نگهداری خانه و خانواده باشد.
3. یک ازدواج که زن و مرد مساویانه برای تهیه و نگهداری خانه و خانواده احساس مسئولیت کنند و یکجائی همه امور را به پیش ببرند.

دو سوال بعدی برای آنانی است که ازدواج کرده اند.

1. ما علاقه داریم بدانیم به چه اندزه امور منزل توسط اشخاص مختلف انجام می پذیرد. کی زیاد تر در امور و کار های خانه کمک میکند مانند آشپزی ، سودا خریدن، حفظ و مراقبت خانه و خانواده
2. خودم
3. همسر
4. مادر
5. پدر
6. خشو
7. خسر
8. پدر کلان
9. نواسه
10. کسی دیگر نفر اول _______________
11. کسی دیگر نفر دوم _______________
12. رد شد جواب نداد
13. جواب ندارد
14. آیا شما فکر میکنید که سهم مناسب در امور منزل داشته باشید؟ یا فکر میکید که شما باید کمتر یا زیاد تر در کارهای خانه حصه بگیرید؟
15. من بطور عادلانه حصه میگیرم
16. باید زیاد تر کمک کنم
17. باید کمتر حصه بگیرم
18. نمی دانم
19. این سوال رد شد
20. جواب ندارد
21. بعد ازین، در مورد اعضای خانواده پرسان می کنیم که کدام یک در خانه همرای شما زندگی میکنند(1= بلی؛ 0= نخیر) (پیشنهاد: پرسان کنید "چند نفر حیات هستند" برای همه مواد تحت الذکر بعدأ بپرسید "کدام یک از اعضای خانواده در حال حلضر با شما زندگی دارد"؟

| چند نفر با شما زندگی میکند | کی را با خود دارید | چند نفر زندگی میکند _____ |
| --- | --- | --- |
| ص. | الف. | جد |
| ض. | ب. | پدر کلان و مادرکلان |
| ط. | پ. | آیا پدرتان زنده است؟ |
| ظ. | ت. | آیا مادر تان زند ه است؟ |
| ع. | ث. | مادر اندر |
| غ. | ج. | کاکا/ماما ها |
| ف. | چ. | عمه ها/خاله ها |
| ق. | ح. | برادران |
| ک. | خ. | چند تن برادر اندر هستند؟ |
| گ. | د. | خواهران |
| ل. | ذ. | چند تن خواهر اندر هستند؟ |
| م. | ر | پسران |
| ن. | ز. | دختران |
| و. | ژ. | نواسه های پسر |
| ه. | س. | نواسه های دختر |
| ی. | ش. | عموزاده ها |

1. به غیر از اعضای خانواده که شما گفتید، دیگر چه کسان در خانه شما زندگی میکند؟ [اعضای اضافی خانوار را به ارتباط رابطه شان به مصاحبه شونده ( مانند دوست ، خشو) فهرست کنید]

__________________________________________________________________________________________________________________________________

1. [ اگر پدر همرا ی مصاحبه شونده زندگی نمیکند] به کدام شهر پدر شما زندگی دارد؟ _________________________________________________________________
2. [اگر مادر و مادر اندر همرای مصاحبه شونده زنددگی نمی کنند] پس به کدام شهر مادر یا مادر اندر شما زندگی دارند؟ _________________________________________________________________
3. چند نفر در خانواده شما زندگی میکنند، اما زیاد تر از یک ماه در خانه نمیباشند به خاطر کار کردن در جاهای دیگر، دیدار اقارب، هجرت و غیره ؟ ___________________
4. بدون کسانی که در خانه شما زندگی میکنند ، چند نفر از اقارب شما در بی ایریا زندگی دارند؟ ( اگر نمی دانید ، تخمینی بگوئید) ___________________
5. بدون کسانیکه در خانه زندگی دارند، چند نفر از اقارب شما درهمسایگی شما زندگی دارند؟ ________ [اگر نمی دانید ، تخمینی بگوئید]

1. بدون کسانیکه در خانه شما زندگی دارند ، چند نفر از اقارب را شما ماهانه می بینید یا زیاد تر از یک بار در ماه؟ ________________________________
2. شما ترجیح میدهید که در یک خانواده ئی دامنه ئی زندگی داشته باشید با سه نسل تحت یک سقف که به دیگر اعضای خانواده نزدیک باشند و یا ترجیح میدهید در یک خانواده ذره وی (آن خانواده ایست که تنها زن و شوهر با اطفال شان زندگی میکنند) و به دیگر اعضای خانواده نزددیک نباشند.
3. خانواده دامنه ئی
4. خانواده ئی ذره وی
5. هردو
6. هیچکدام

لطفأ سوالات بعدی را در رابطه به اولاد تان و نواسه های تان که در امریکا زندگی دارند و هنوز عروسی نکرده اند جواب گوئید. اگر اولاد ندارید ، لطفأ چنین جواب گوید که اگر اولاد میداشتید چگونه جواب میگفتید

1. در رابطه با کی شما این سوالات را جواب می گوئید؟
2. نواسه های من
3. اولاد های من
4. اولاد آینده من
5. به چه اندازه برای شما اهمیت دارد (داشته است و یا خواهد داشت) که (اولاد شما یا هر کسی دیگر )با یک افغان ازدواج کند؟
6. مهم نیست
7. تا اندازه ئی مهم است
8. بسیار اهمیت دارد
9. فکر میکنید که (اولاد شما یا هر کسی دیگر) از خانواده اجازه داده شود که همسر خود را انتخاب کند وقتیکه به سن ازدواج برسند؟
   1. نخیر ، برای والدین بهتر است که تصمیم خود را بگیرند
   2. تا اندازه ئی. اولاد ها و والدین باید یکجائی بالای این موضوع کار کنند تا یک همسر خوب پیدا کنند
   3. بلی ، اولاد ها باید بالای شان اعتماد شود که خود شان درین مورد تصمیم گیرند.
10. فکر میکنید که (اولاد شما یا هر کسی دیگر) بیرون رود جنس مخالف خود را آزادانه دیدار کند بعد ازینکه مکتب را تمام کردند؟
11. بلی.
12. نخیر
13. متیقین نیستم
14. به چه اندازه به شما اهمیت دارد که (اولاد شما یا کسان دیگر) با کسی ازدواج کند که هم دین شما با شد؟
15. مهم نیست
16. تا اندازه دی مهم است
17. بسیار مهم است

دین

1. به کدام دین هستید؟
2. اسلام
3. عیسویت
4. هندو
5. یهود
6. سیک
7. بودائی
8. دیگر_________
9. هیچ یک

(اگر مسلمان نیستید از سوال بعدی تیر شوید)

1. اگر مسلکان هستید ، میتوانید زیاتر مشخص بسازید ؟
2. سنی حنفی
3. جعفری دوازده امامی
4. جعفری اسماعلیه
5. کدام مذهب دیگر _________________________
6. شما به مسجد به خاطر عبادت میروید یا به خاطربدست آوردن کمک اجتماعی میروید؟
7. بلی
8. نخیر
9. تا چه اندازه در مسجد برای خدمت مذهبی و یا به نمازخواندن میروید؟
10. زیاد تر از یک بار در هفته
11. هفته یکبار
12. از یک تا سه بار در ماه
13. کمتر از یکبار در ماه
14. هیچ نمی روم
15. به کدام اندازه دین اسلام در زندگی رزومره شما اهمیت دارد؟
16. مهم نیست
17. تا اندازه ئی مهم است
18. بسیار مهم است
19. شما فکر میکنید مهم است که تمام مردم به دین های غیر از دین خود شان احترام داشته باشند و هم جور بیایند همرای آن مردم که از دیگر دین ها هستند؟
20. بلی ، شدید موافق هستم
21. بلی، تا اندازه ئی موافق هستم
22. نخیر، تا اندازه ئی موافق نیستم
23. نخیر ، مطلقأ موافق نیستم
24. در رمضان به چه اندازه روزه میگیرید؟
25. شدیدأ، من بصورت جدی مقررات روزه را تعقیب میکنم
26. تا اندازه که میتوانم، مقررات روزه را تعقیب میکنم
27. اجتماعی، در اجتماع عامه مقررات روزه را مراعات میکنم نه در امور شخصی
28. بسیار کم یا هیچ نه

**صدمه و فشار روانی در اسر وقوع جنگ و مهاجرت جبری . {این قسمت برای افغان های مهاجر است. اگر مصحابه شونده ( در سن 9 سالگی یا پایینتر از آن) افغانستان را ترک کرده باشد، از آن بپرسید "به خاطر دارید خطرها و خوشونت ها را در افغانستان؟" اگر مصحابه شونده چیزی را به خاطر ندارد از این قسمت تیر شوید}.**

سوال های بعدی در مورد تجارب شما با جنگهای شدید در افغانستان است. اگر یکی از این سوال ها شما را ناراحت میسازد لطفأ برایم بگوید تا به سوال های دیگر رجوع نمایم.

1. پیش ازینکه افغانستان را ترک گفتید ، آیا شما بطور کافی معاش داشتید تا احتیاجات شما را رفع کند؟
2. معاش به قدر کافی نبود که احتیاجات ما مرفوع شود
3. معاش ما برای تکافوی احتیاجات ما سر و نوک میشد
4. معاش کافی بود اما برای پس انداز چیزی نمی ماند
5. معاش کافی و کمی زیاد تر از چیزی که توقع داشتم
6. زیاد تر از احتیاجات خود معاش داشتم.
7. آیا امنیت شما و یا زندگی تان در جریان کودتا و جنگ های داخلی در افغانستان به مخاطره افتیده بود و یا وقت که از افغانستان فرار کردید؟
8. بلی
9. نخیر
10. آیا شما هرگز توسط قوای عسکری ، پولیس ، تنظیم های مجاهدین ، طالبان و یا دیگر گروه های ذیدخیل در جنگ حبس بندی، گرفتار و یا گروگان شده اید؟
11. بلی
12. نخیر
13. اگر جواب شما بلی است ، پس آیا شما شکنجه و صدمه دیده اید وقتیکه در حبس بودید.
14. بلی
15. نخیر
16. [اگر جواب دریکی از سه سوال قبلی بلی بوده باشد . اگر به هر سه جواب نی بوده باشد از چهار سوال بعدی تیر شوید] آیا شما در نتیجه جنگ ها و دهشت افگنی/ترورزم در افغانستان زخمی و یا معیوب شده اید؟
17. بلی
18. نخیر
19. اگر جواب بلی است پس چه نوع شما زخمی شده اید؟ ________________________________ _________________________________________________________________
20. آیا هیچ یک از اعضای نزدیک خانواده شما و یا دوستان نزدیک تان در یکی از کودتا ها و جنگ ها در افغانستان حیات شان در خطر بود؟
    - - 1. بلی
        2. نخیر
21. آیا هیچ یک از اعضای نزدیک خانواده شما و یا دوستان نزدیک تان در یکی از کودتا ها و جنگ ها در افغانستان زخمی شده و یا صدمه دیده اند ؟
22. بلی
23. نخیر
24. آیا هیچ یک از اعضای نزدیک خانواده شما و یا دوستان نزدیک شما در یکی از کودتا ها و جنگ ها کشته شده و یا مفقود شده است؟
25. بلی
26. نخیر
27. اگر جواب بلی است ، چند نفر از اعضای خانواده شما و یا دوستان نزدیک شما کشته و یا مفقود شده است؟ __________________________________________________________________
28. آیا شما شاهد کشته شدن و زخمی شدن اعضای نزدیک خانواده و یا دوستان نزدیک تان بوده اید؟
29. بلی
30. نخیر
31. شما تشریح کردید( خلاصه وقایع دلخراش را که مصاحبه شونده تجربه کرده بگوئید) آیا این همه وقایع باعث به وجود آوردن تکالیف روحی ، ترس و واهمه شده است؟
32. بلی
33. نخیر
34. اگر جواب بلی است لطفأ رنج های روحی و روانی تانرا تشریح کنید. چگونه این همه وقایع دلخراش باعث تشویش روحی و روانی شما شده است؟ ____________________________________________________________________________________________________________________________________________________________________________________________________________________________________________________________________
35. اگر جواب بلی است، چگونه شما این معضله را مقابله کردید. چه کردید که از ناآرامی های روحی نجات پیدا کنید؟ ____________________________________________________________________________________________________________________________________________________________________________________________________________________________________________________________________
36. در آن وقت آیا شما کمک شدید تا ازین تکالیف روحی و روانی علاج شوید؟
37. بلی
38. نخیر
39. کی این کمک ها را برای شما فراهم کرد؟ ( مشخص سازید اگر ممکن باشد) __________________________________________________________________________________________________________________________________
40. چگونه افغانستان را ترک گفتید و یا فرار کردید ( هر ترتیب که پائین وفق می کند انتخاب نمائید)
    1. موتر/لاری
    2. به پای
    3. ترین
    4. طیاره
    5. موتر سرویس
    6. طریق دیگر_________________________________________
41. بعد از ترک افغانستان ، آیا به کمپ های مهاجرین پناهنده شدید؟
    1. بلی
    2. نخیر

**هفت سوال بعدی برای کسانیست که در کمپ های مهاجرین اقامت گزیده اند**

1. کمپ های مهاجرین در کجا موقیعت داشتند؟ _____________________________________ __________________________________________________________________
2. برای چه مدت در کمپ های مهاجرین اقامت داشتید؟ (اگر در زیاد تر از یک کمپ بوده باشید مجموع کل اقامت تانرا بگوئید)
3. 1- 3 ماه
4. 4 - 6 ماه
5. 7- 9 ماه
6. 10- 12 ماه
7. 13- 18 ماه
8. 19-24 ماه
9. 25 ماه تا 3 سال
10. 4- 5 سال
11. 6- 7 سال
12. 8 - 9 سال
13. ده سال یا زیاد تر.
14. آیا به قدر کافی غذا و آب در کمپ های مهاجرین داشتید؟
15. بلی ، بسیاری اوقات
16. نخیر، بعضی اوقات غذا و آب کافی نبود
17. نخیر، اکثرأ غذا و آب کافی نبود
18. آیا شما سهولت های کافی صحی در کمپ های مهاجرین داشتید؟
19. بلی ، بسیار اوقات و یا همه وقت
20. نخیر، بعضی اوقات به مجروحین و مریضان رسیدگی در ست نمی شد.
21. نخیر ، اکثرأ به مریضان و مجروحین رسیدگی صورت نمی گرفت.
22. آیا زندگی شما به مخاطره افتیده است و یا شدیدأ صدمه دیده اید وقتیکه در کمپ های مهاجرین بودید؟
23. بلی
24. نخیر.
25. آیا شما به تکلیف روحی و روانی دچار شده بودید وقتیکه در کمپ مهاجرین بودید؟
26. بلی
27. نخیر
28. آیا یکی از اعضای خانواده شما و یا دوستان نزدیک شما شدیدأ صدمه دیده است و یا مریض شده اند وقتیکه در کمپ های مهاجرین بودید؟
29. بلی
30. نخیر

خاتمه گذشتن از سوالات کمپ های مهاجرین – همه سوال های بعدی را جواب می گوید.

1. آیا شما رنج می برید که اعضای خانواده شما با شما نیستند و یا نتوانستند کشور را ترک گویند؟
2. بلی، بسیار زیاد
3. بلی، تا اندازه ئی
4. نخیر
5. آیا آرزو دارید که خانواده تان را یکجا کنید؟
6. بلی
7. نخیر
8. اگر جواب بلی است ، به چه اندازه برای شما مهم است که با خانواده تان یکجا شوید؟
9. بسیار مهم است
10. مهم است
11. بسیار مهم نیست

حالا ما از شما در مورد اقامت گزینی در امریکا و بی ایریا می پرسیم.

1. (اگر در امریکا تولد شده باشد ازاین سوال صرف نظر کنید) شما گفتید که شما/فامیل شما به امریکا رسیدید (اشاره به سوال 23) . همرای کی شما آمدید وقت که باراول به امریکا آمدید؟
   1. پدر کلان و مادر کلان
   2. مادر
   3. پدر
   4. همسر
   5. خواهر و برادر
   6. اولاد
   7. نواسه ها
   8. دیگران____________________________
   9. صرف خودتان
2. (اگر در امریکا تولد شده باشد ازاین سوال صرف نظر کنید) چند نفر از اعضای خانواده شما در امریکا زندگی میکردند وقتیکه شما/ خانواده شما به امریکا رسیدید؟_________ (اگر متیقن نیستید ، تخمینی بگوئید)
3. (اگر در امریکا تولد شده باشد ازاین سوال صرف نظر کنید) چند نفر دوست نزدیک شما/ یا خانواده شما در امریکا داشتید وقتیکه ا بار اول رسیدید؟___________ (اگر متیقن نیستید ، تخمینی بگوئید).
4. ( اگر در بی اریا تولد شده باشد از این سوال صرف نظر گردد) شما گفتید که شما/فامیل شما در بی ایریا رسیدید (اشاره به سوال 23) کی شما را به بی ایریا کوچ داد؟
   1. پدر کلان و مادر کلان
   2. مادر
   3. پدر
   4. همسر
   5. خواهر و برادر
   6. اولاد
   7. نواسه ها
   8. دیگران____________________________
   9. صرف خودتان

(9 سوال بعدی را بگذارید اگر مصاحبه شونده در سن 10 سالگی به بی اریا رسیده باشد "12-24")

1. بطور عموم به چه اندازه مشکل بود که شما و خانواده تان در بی ایریا اقامت کنید و زندگی را عیار سازید. می گویئ که :
   - - 1. بسیار مشکل نیود
       2. کمی مشکل بود
       3. بسیار مشکل بود
2. (اگر کمی مشکل و یا بسیار مشکل بوده باشد) بزرگترین مشکلات شما /فامیل شما در وقت اقامت گزین شدن در بی ایریا چه بود؟

__________________________________________________________________________________________________________________________________

1. به چه اندازه شما/خانواده شما از دوستان و خانواده های که قبلا اینجا زندگی میکردند کمک گرفتید وقتیکه بار اول به بی ایریارسیدید؟
2. هیچ
3. کمی
4. بطور متوسط
5. بسیار زیاد
6. چقدر کمک شما/ خانواده تان از سازمان های افغانی و دیگر موسسات غیر انتفاعی دریافت کردید وقتیکه بار اول به بی ایریا رسیدید؟
7. هیچ
8. یک کمی
9. بطور متوسط
10. بسیار زیاد
11. آیا شما و یا خانواده شما از کدام سازمان افغانی و یا دیگر موسسات غیر حکومتی از وقتیکه به امریکا رسیده اید کمک دریافت کرده اید؟
12. بلی
13. نخیر
14. اگر جواب بلی باشد، از کدام سازمان افغانی کمک گرفته اید و چگونه شما را کمک کرده اند (مثلا خانه پری اوراق مهاجرت ، خانه پالی ، کار یابی ، و امور صحی)؟

____________________________________________________________________________________________________________________________________

__________________________________________________________________

1. بطور عموم ، به چه اندازه ، شما فکر میکنید که سازمان های افغانی برای افغانهای که در بی ایریا زندگی میکند موثر بوده است؟
2. موثر نیست
3. تا اندازه ئی موثر است
4. بسیار موثر
5. آیا شما و یا اعضای خانواده شما که در خانه همرایتان زندگی میکند از دولت فدرال، ایالتی و یا کونتی کمک دریافت کرده اید وقتیکه به بی ایریا رسیدید؟
6. بلی
7. نخیر
8. اگر جواب بلی باشد ، چه خدمات را شما حصول کرده اید؟ (دور همه آنرا که گرفته اید خط بکشید)
9. بیمه اجتماعی یا امنیت اجتماعی(سوشل سیکوریتی)
10. کمک های صحی مانند مدیکل ، میدیکید و یا میدیکیر
11. کمک های معیوبی
12. کوپان غذائی
13. کمک های عمومی یا نقدی
14. کمک ها برای خانه و یا برای خانه های تعاونی.
15. کمک های اطفال و یا کمک های تعاونی اطفال.
16. بیمه بیکاری
17. کمک های تعلیمی
18. مشوره های روحی و روانی
19. دیگر کمک ها____________________________________________

خاتمه تیرشدن سوالات برای سنین پائین تر از ده که به بی اریا رسیده باشد.

1. آیا کسی در خانواده شما از دولت فدرال ، ایالتی و یا کونتی فعلا کمک میگیرد؟
2. بلی
3. نخیر
4. کدام خدمات اجتماعی را فعلا اعضای خانواده شما در یافت میکنند ( دور آنرا خط بکشید)
5. بیمه اجتماعی یا امنیت اجتماعی(سوشل سیکوریتی)
6. کمک های صحی مانند مدیکل ، میدیکید و یا میدیکیر
7. کمک های معیوبی
8. کوپان غذائی
9. کمک های عمومی یا نقدی
10. کمک ها برای خانه و یا برای خانه های تعاونی.
11. کمک های اطفال و یا کمک های تعاونی اطفال.
12. بیمه بیکاری
13. کمک های تعلیمی
14. مشوره های روحی و روانی
15. دیگر کمک ها__________________________________________________
16. کدام یک از خدمات اجتماعی و حمایوی را شما و یا خانواده شما اشد ضرورت دارد که فعلا شما دریافت نمی کنید؟ _________________________________________________________

________________________________________________________________

1. در سال گذشته شما و یا خانواده شما به اقارب و دوستان که در افغانستان ، پاکستان و ایران زندگی میکند پول فرستاده اید؟
2. بلی
3. نخیر
4. اگر جواب بلی است بطور تخمینی چند دالر فرستاده اید؟__________________

[اگر ضرورت باشد همرایشان در ارقام تخمینی کمک کنید که چند مرتبه و چه اندازه هر مرتبه فرستاده اند]

1. مقصد شما از کدام یک اسعار است؟ دالر امریکائی. روپیه پاکستانی، ریال ایرانی، یورو و یا چه نوع پول؟
2. دالر امریکائی
3. روپیه پاکستانی
4. ریال ایرانی
5. یورو
6. نوع دیگر اسعار_________________
7. در سال گذشته ، آیا شما به زنان بیوه و اطفال ویا اعمار مجدد افغانستان کمک خیریه کرده اید ؟
8. بلی
9. نخیر
10. آیا کمک خیریه که کردید برای شما/ خانواده تان کدام مشکل اقتصادی را به بار نیاآورده درسال گذشته؟
    1. بلی
    2. نخیر
11. چقدر شما و یا اعضای خانواده شما با اقارب و دوستان در داخل افغانستان به تماس هستید؟
12. روزانه
13. هفته وار
14. ماهانه
15. سالانه
16. کمتر از یک بار در سال
17. هیچ وقت
18. از کدام طریق شما و یا اعضای خانواده شما با اقارب و دوستان در داخل افغانستان تماس حاصل می کنید؟
19. تیلفون
20. ایمل
21. فکس
22. نامه
23. طریق دیگر________________________
24. بدون از اعضای خانواده خود شما چند نفر افغان در همسایگی شما زندگی میکند؟
25. کدام افغان دیگر در همسایگی ما نیست
26. چند نفر محدود در همسایگی ما هستند و اما زیاد نی
27. یک تعداد زیاد افغانها در همسایگی ما زندگی میکنند
28. به کدام اندازه با دیگر افغانها رادر همسایگی تان در تماس هستید و یا ملاقات می کنید؟
29. هیچ وقت
30. وقتأ فوقتأ
31. بعضی ائقات
32. اکثرأ
33. چند نفر دوست نزدیک در بی ایریا دارید که میتوانید در موقع ضرورت سر آنها حساب کنید؟___
34. چند نفر از دوستان نزدیک شما در بی ایریا افغان نیستند؟______________
35. آیا شما و یا اعضای خانواده شما علاقمند هستید که در آسایشگاه کلان سالان و یا محیط زسیت کلان سالان که برای یک زندگی افغانی طرح شده باشد و خدمت کند ، زندگی کنید؟
36. بلی
37. ممکن است
38. نخیر

تعلیم و تربیه:

1. (کسانی را پرسان کنید که در افغانستان بعد از سن پنج زندگی کرده اند) آیا در افغانستان مکتب رفتید؟
   1. نخیر (از سوال بعدی تیر شوید )
   2. بلی
2. به کدام سطح در افغانستان درس خواندید؟
3. کمی ابتدائیه
4. ابتدائیه را تمام کردم
5. کمی متوسطه
6. متوسطه را تمام کردم
7. کمی لیسه
8. لیسه را تمام کردم
9. مکتب تجارتی
10. کمی فاکولته
11. فاکولته را تمام کردم
12. داکتر طب
13. کدام دیپلوم پیشرته مسلکی دیگر
14. دکتورا
15. دیگر مدرک تحصیلی__________________
16. آیا در امریکا مکتب رفتید؟
17. بلی
18. نخیر
19. به کدام سطح در امریکا درس خواندید و یا تمام کردید؟
20. ابتدائیه
21. متوسطه و یا صنف دوازده
22. فارغ التحصیل صنف دوازده
23. جی ای دی
24. درجه از یک مکتب تجارتی ___________________
25. درجه تخنیکی از یک کمونیتی کالج
26. کالج دوساله
27. کالج چهار ساله
28. ماستری
29. درجه های مسلکی مانند دکتورای طب یا دکتورای حقوق
30. دکتورا
31. تصدیقنامه زبان انگلیسی
32. مدرک دیگر___________________________
33. (اگر مصاحبه شونده از مکتب تجارتی کمونیتی کالج و یا یونیورسیتی در امریکا فارغ شده باشد) از کدام مکتب بلند ترین درجه تحصیلی را دریافت کرده اید؟
34. شبو کالج
35. اولونی کالج
36. یونیورسیتی ایالتی –ایست بی
37. سن هوزی ایستیت
38. سا فرانسیسکو ایستیت
39. یونیورسیتی کلیفورنیا – سان فرانسیسکو
40. یونیورسیتی کلیفورنیا – برکلی
41. یونیورسیتی کلیفورنیا – دیوس
42. دیگر مکاتب_______________________________
43. (اگر مصاحبه شونده درجه پیشرفته دارد مانند ماستری و دکتورا) رشته تخصصی شما چه است؟

________________________________________________________________

1. (اگر جواب نی است ، ازین سوال تیر شوید) تحصیلات عالی را در یک کشور دیگر به غیر از افغانستان یا امریکا به پایان رساندید؟
2. بلی
3. نخیر
4. اگر جواب بلی است ، در کدام کشور تحصیلات خویش را به پایان رسانده اید و به کدام درجه؟ _______________________________________________________________
5. بلند ترین درجه تحصیل مادر تان در کدام سطح میباشد؟ ____________________________
6. بلند ترین درجه تحصیل پدر تان در کدام سطح میباشد؟ ______________________________
7. قبلأ شما گفتید که ________ بچه و _________ دختر دارید. آیا اولاد شما در امریکا تحصیل کرده است.
8. بلی
9. نخیر (سوال بعدی را تیر شوید)
10. سن ، جنس و بلند ترین سطح تحصیل اولاد شما که در امریکا درس خوانده باشند تا حال چه است؟

(1= زن، 2= مرد)

0. هیچ

سن جنس تحصیل 1. کمی مکتب ابتدائیه

طفل #1 الف___ ذ___ ع___ 2. مکتب ابتدائیه را تکمیل کردم

طفل #2 ب____ ر___ غ___ 3. کمی مکتب متوسطه

طفل# 3 پ____ ز___ ف___ 4. مکتب متوسطه تکمیل کردم

طفل #4 ت____ ژ___ ق___ 5. کمی لیسه

طفل#5 ث____ س___ ک___ 6. بکلوریا

طفل#6 ج____ ش___ گ___ 7. جی ای دی

طفل#7 چ____ ص___ ل____ 8. مکتب تجارت را تمام کردم

طفل#8 ح____ ض___ م____ 9. دوسال درجه تخنیکی

طفل#9 خ____ ط____ ن____ 10. کمی کالج بدون دیپلوم

طفل#10 د____ ظ____ و____ 11. کالج دو ساله تمام کردم

12. لیسانس

13. ماستری

14. درجه تخصصی

15.دکتورا

شغل و کار:

1. کدام یک عبارت ذیل بهتر موقیعت کار شما را تشریح میکند؟
2. کار دارم، 45 ساعت یا زیاد تر در هفته ( از سوال بعدی تیر شوید)
3. کار دارم، 35-44 ساعت در هفته (از سوال بعدی تیر شوید)
4. کار دارم، 25-34 ساعت در هفته ( از سوال بعدی تیر شوید)
5. کار دارم، کمتر از 25 ساعت در هفته(از سوال بعدی تیر شوید)
6. بیکار، کار می پالم
7. بیکار ، پشت کار نمی گردم
8. خانه نگهداری
9. متقاعد
10. معیوب
11. حالت دیگر مشخص کنید__________________________
12. چند سال است که کار میکنید؟ (پنج سوال بعدی را تیر شوید بعد از جواب این سوال)
13. کمتر از یک سال
14. بین یک تا سه سال پیش
15. بین سه تا پینج سال پیش
16. بین پینج تا ده سال پیش
17. زیاد تر از ده سال
18. هیچ وقت کار نگرفتم
19. آیا شما از ساعات که کار دارید راضی هستید ، یا ترجیح میدهید که زیاد تر کار کنید یا کمتر کار کنید؟
20. از ساعات کار خود راضی هستم
21. خوش دارم که زیاد تر کارکنم
22. خوش دارم که کمتر کار کنم
23. وظیفه اصلی شما چه هست، چه کار میکنید ( وظایف عمده) و در چه نوع جای شما کار می کنید؟

الف. شغل و وظایف اصلی _______________________________________________

_________________________________________________________________

ب. نوع کار(شرکت چه کار و تولید می کند؟ یا در کدام نمایندگی حکومتی یا سازمان شما کار میکنید؟)

_________________________________________________________________

1. کدام یک ازین جملات بهتر وظیفه اصلی شما راتشریح میکند؟
2. این کار به من موقع را نمی دهد که از تحصیل ، تجربه و قابلیت خود استفاده کنم. من می توانم که کار های پیچیده را انجام دهم و مسئولیت زیاد تر بر عهده گیرم.
3. این کار موقع را برای من می دهد که از تحصیل، تجربه و قابلیت های خود استفاده کنم . کار من جنجالی است و باید چیلنج (دست و پینجه به آن نرم کرد).
4. نظر به کار اصلی شما، کدام یک بهتر همکاران شما را تشریح میکند؟
5. زیاد تر افغان هستند
6. زیاد تر غیر افغان هستند ، متولد خارجی میباشند
7. زیاد تر غیر افغان و امریکائی هستند.
8. (تنها از کسانی پرسان کنید که سن شان از هژده به بالا بوده که افغانستان را ترک کرده اند) آیا شما یک کار با درآمد در افغانستان داشتید پیش ازینکه به امریکا مهاجرشدید؟
9. بلی
10. نخیر(دوسوال بعدی را تیر شوید)
11. وظیفه اصلی شما قبل ازینکه به امریکا مهاجرشوید چه بود (لطفأ دقیق تشریح کنید) و در کجا کار میکردید؟

الف. شغل و وظایف اصلی______________________________________________

________________________________________________________________

ب. کار فرما (employer). ( شرکت که کار میکردید چه میساخت؟ یا کدام شعبه حکومتی یا موسسه کار میکردید؟) _______________________________________________________

_________________________________________________________________

1. وقتیکه شما در امریکا کار گرفتید ، به چه اندازه کارفرما نو تجربه کار شما را قدردادند؟
2. الف. بسیار کم
3. یک اندازه کم
4. بسیار زیاد
5. (در صورتیکه متاهل باشد بپرسید) کدام یک از این ها بهترحالات کار همسر شما را فعلا توضیح/بیان میکند؟
6. مشتغل، 45 ساعت یا زیاد تر در هفته ( از سوال بعدی تیر شوید)
7. مشتغل، 35-44 ساعت در هفته (از سوال بعدی تیر شوید)
8. مشتغل، 25-34 ساعت در هفته ( از سوال بعدی تیر شوید)
9. مشتغل، کمتر از 25 ساعت در هفته(از سوال بعدی تیر شوید)
10. بیکار، کار می پالم
11. بیکار ، پشت کار نمی گردم
12. خانه نگهداری
13. متقاعد
14. معیوب
15. حالت دیگر مشخص کنید________________________
16. چند سال است که همسر شما کار میکند؟
17. کمتر از یک سال
18. بین یک تا سه سال پیش
19. بین سه تا پینج سال پیش
20. بین پینج تا ده سال پیش
21. زیاد تر از ده سال
22. هیچ وقت کار نکرده

1. کار اصلی همسر شما چه هست، چه کار میکند ( وظایف عمده) و در چه قسم محل کار می کنید؟
2. کار وظایف اصلی ________________________________________________
3. نوع/قسم کار(شرکت چه کار و تولید می کند؟ یا کدام نمایندگی حکومتی یا سازمان شما مشغول هستید؟) اگر همسر کار شخصی در خانه میکند ، تذکر دهید.
4. آیا همسر شما یک کار با درآمد در افغانستان داشت پیش ازینکه به امریکا مهاجرشود؟
5. بلی
6. نخیر
7. شغل اصلی همسر شما قبل ازینکه به امریکا مهاجرشود چه بود (لطفأ دقیق تشریح کنید) و در کجا کار میکرد؟
8. الف. کار و وظایف عمده _____________________________________________

______________________________________________________________

1. کار فرما (employer). ( شرکت که کار میکردید چه میساخت؟ یا کدام شعبه حکومتی یا موسسه کار میکردید؟) ______________________________________________________________

______________________________________________________________

1. آیا شما یا یک عضو نزدیک خانواده شما خوش دارد تا به یک تماس زنجیری ذات البینی کار یابی افغانی باشد تا بدان وسیله شود که شما بتوانید کار های کارگری روزرانه را بین یک تا سه روز در هفته بکنید مانند کار در حویلی ها کار تعمیرات و یا کار های که روی سرک و زمین میباشد؟
2. بلی
3. نخیر
4. فکر نمی کنم
5. نمی دانم
6. سوال جواب داده نشد
7. آیا شما و یا یک عضو خانواده شما خوش دارد تا در کورس های آموزشی دو سه روزه به دری و انگلیسی شرکت کنید تا به شما بیاموزاند تا چگونه کار پیدا کنید، چطور اوراق/فورمه کار یابی را خانه پری کنید و چگونه خود را در جریان مصاحبه کار خود را معرفی کنید؟
8. بلی
9. نخیر
10. فکر نمی کنم
11. نمی دانم
12. سوال جواب داده نشد
13. عاید/کمایی کلی خانوار شما در دوازده ماه اخیر چند بود؟

1. 0-4.999 دالر 6. 30.000 -39.999 دالر 11. 80.000-89.999 دالر

2. 5000-9.999 دالر 7. 40.000-49.999 دالر 12. 90.000-99.999 دالر

3. 10.000-14.999 8. 50.000-59.999 دالر 13. 100.000-249.999 دالر

4. 15.000-19.999 9. 60.000-69.999 دالر 14. 250.000 یا زیاد تر

5. 20.000-29.999 10. 70.000-79.999 دالر

تبعیض و قضاوت های قبلی اجتماعی:

1. از وقتیکه در بی ایریا زندگی میکنید آیا مواجه به توهین زبانی ، طعنه نژادی ، لهجه و ریشخند قرار گرفته اید به خاطریکه شما افغان هستید و یا به خاطریکه طرف مقابل شما عوضی گرفته باشد و فکر کرده که عرب هستید؟
2. بلی
3. نخیر (سوال بعدی تیر شوید)
4. در حدود چند بار شما توهین شده اید؟
5. یک بار
6. دو بار
7. سه یا چهار بار
8. پینج یا شش بار
9. هفت تا نه بار
10. ده بار یا زیاد تر
11. از وقتیکه در بی ایریا زندگی میکنید آیا به شما حمله جسمی/فزیکی صورت گرفته به خاطریکه شما یک افغان هستید و یا حمله کنند شما را عوضی گرفته باشد و فکر کرده باشد که شما عرب هستید؟
12. بلی.
13. نخیر ( از سوال بعدی تیر شوید)
14. در حدود چند بار به شما حمله جسمی/فزیکی صورت گرفته؟
15. یک بار
16. دو بار
17. سه یا چهار بار
18. پینج یا شش بار
19. هفت تا نه بار
20. ده بار یا زیاد تر
21. بعد از واقعه تاسف انگیز یازدهم سپتامبر ، آیا شما زیاد تر پریشان بودید که مردم به شما و یا خانواده تان صدمه بزند؟
22. بلی، بسیار پریشان بودم
23. بلی، تا اندازه ئی پریشان بودم
24. نخیر، پریشان نبودم
25. بعد از حادثه یازدهم سپتامبر وقتیکه شما به میدان های هوائی می رفتید ، آیا شما را از ردیف مردم جدا می کردند و زیاد تر پرس و پال میکردند و بکس سفری تانرا دقیقتر تلاشی و بررسی میکردند؟
26. نخیر، من بعد از یازده سپتامبر قطعأ سفر نکرده ام
27. نخیر، من پرواز کرده ام اما تلاشی نشده ام.
28. بلی، یک بار
29. بلی، دو بار
30. بلی، سه بار
31. بلی، چها ر بار
32. بلی ، هفت بار یا زیاد تر
33. تبعیض وقتی است که یک شخص شخصی دیگر را به خاطر که از گروه آن شخص نیست ، صدمه میزند. طور مثال یک کار فرما یک افغان و یا مسلمان را استخدام نمی کند به خاطر اینکه درخواست کننده مسلمان و یا افغان است. این تبعیض است. از وقتیکه در بی ایریا زندگی میکنید آیا در مقابل شما در وقت درخواست کار یا ترفیع در کار ، درخواست برای خانه ، دخول در مکتب یا از طریق دیگر تبعیض صورت گرفته به خاطریکه شما یک افغان هستید ؟
34. بلی، یقینأ
35. بلی، من یقین دارم
36. نخیر ، من فکر نمی کنم (سوال بعدی را تیر شوید)
37. نخیر ، هرگز نی(سوال بعدی را تیر شوید)
38. اگر جواب بلی باشد چگونه و چطور در مقابل شما تبعیض صورت گرفت (هر سوال که به شما صدق میکند، خط کنید)
39. وقتیکه درخواست کار کرده ام
40. تقاضای ترفیع کرده ام یا به طور غیر عادلانه کار من ارزیابی شده است
41. وقتیکه یک خانه و یا آپارتمان را به کرایه می گرفتم
42. وقتیکه یک آپارتمان و یا خانه را خریداری میکردم
43. وقتیکه برای شامل شدن در یکی از یونیورسیتی ها و یا برنامه های تعلیمی درخواست کرده ام
44. وقتیکه در مکتب بودم توسط معلم و یا مشاور تبعیض صورت گرفته
45. توسط پولیس
46. در محکمه
47. در یک مغازه و یا رستوران
48. طریق دیگر شماره اول . مشخص کنید______________________________
49. طریق دیگر شماره دوم. مشخص کنید______________________________
50. شما فکر میکنید که افغان های که مساویانه با امریکائیان لیاقت کار را دارند ، فرصت مساوی در کار گرفتن دارند؟
51. بلی
52. نخیر
53. فکر نمی کنم
54. شما فکر میکنید که اکثریت امریکائی ها به افغانها اعتماد ندارند؟
55. بلی
56. نخیر.
57. فکر نمی کنم
58. مسایل فرهنگی:
59. (پرسان کنید در صورتیکه والدین زنده هستند)
60. به کدام زبان با والدین خود صحبت می کنید؟
61. تنها دری یا پشتو
62. تنها انگلیسی
63. زیاد تر دری یا پشتو اما کمی انگلیسی
64. زیاد تر انگلیسی اما کمی دری یا پشتو
65. دری و پشتو و انگیسی به طور مساوی
66. کدام زبان دیگر مشخص کنید_______________
67. من پدر و مادر ندارم
68. (پرسان کنید اگر اولاد دارند) به کدام زبان با اولاد تان صحبت می کنید؟
69. تنها دری یا پشتو
70. تنها انگلیسی
71. زیاد تر دری یا پشتو اما کمی انگلیسی
72. زیاد تر انگلیسی اما کمی دری یا پشتو
73. دری و پشتو و انگیسی به طور مساوی
74. کدام زبان دیگر مشخص کنید_______________
75. من اولاد ندارم.
76. وقتیکه تلویزیون می بینید ، به چه اندازه برنامه های افغانان را تماشا می کنید؟
    1. هیچ وقت
    2. بعضی اوقات ، کمتر از نصف وقت
    3. زیاد تر از نصف وقت اما همه وقت نی
    4. همه وقت تماشا می کنم
    5. من تلویزیون تماشا نمی کنم
77. کدام یک از هنر پیشه گان ، سرایندگان موسیقدانان نویسندگان و شاعران را با نظر داشت ماهیت قومی او زیاد تر قدردانی می کنید؟
78. همه افغان هستند
79. زیاد تر افغان هستند
80. در حدود نصف افغان هستند و نصف غیر افغان
81. زیاد تر غیر افغان/خارجی هستند
82. همه غیر افغان/خارجی هستند
83. چه قسم اخبار و مجله را مطالعه می کنید؟
84. تنها به زبان دری و پشتو
85. زیاد تر به زبان دری و پشتو
86. هردو به زبان دری و پشتو و به زبان انگلیسی مساویانه
87. زیاد تر به زبان انگلیسی
88. تنها به زبان انگلیسی
89. زبان دیگر. مشخص کنید________________
90. چه قسم غذا عموما در خانه میخورید؟
91. تنها غذای افغانی
92. زیاد تر غذای افغانی
93. نصف افغانی و نصف غیر افغانی/جارجی
94. زیاد تر غذای غیر افغانی/خارجی
95. همه غیر افغانی/خارجی
96. آیا شما عمومأ در جشن سال نو (نوروز) در پلیزنتن سهم می گیرید؟
97. بلی
98. نخیر
99. شما به یک کتابخانه که بطور قابل ملاحظه کتاب ها و اخبار و مجلات به زبان های دری و پشتو داشته باشد دسترسی دارید؟
100. بلی
101. نخیر
102. فکر نمی کنم
103. نمی دانم
104. سوال جواب داده نشد
105. اگر همچو کتابخانه که کتب ، مجلات، اخبار و دیگر مواد به زبان دری و پشتو داشته باشد ، شما استفاده می کنید؟
106. نخیر
107. بلی ، به ندرت
108. بلی بعضی اوقات
109. بلی ، اکثر اوقات
110. بلی ، اکثرأ
111. در دوازده ماه گذشته آیا کدام کار افتخاری در سازمان های افغانی کرده اید؟
112. نخیر
113. بلی
114. در دوازده ماه گذشته آیا کدام کار افتخاری در موسسات مدنی ، سازمان های خیریه مانند روتری ، سازمان معلم و والدین و یا مرکز مذهبی یا سپورتی انجام داده اید؟
115. نخیر
116. بلی
117. آیا شما در محافل علمی و هنری فرهنگی افغانی مانند فلم ها، کنسرت ها، مشاعره و سخنرانی ها اشتراک کرده اید؟ اگر جواب بلی است ، چند بار؟
118. نخیر
119. بلی، یک بار
120. بلی دو بار
121. بلی سه بار
122. بلی، چهار بار یا زیاد تر
123. آیا شما آرزو دارید که محافل فرهنگی افغانی در بی ایریا زیاد تر شود؟
124. بلی
125. نخیر
126. آیا شما برای رأی دهی ثبت نام کرده اید؟
127. بلی
128. نخیر
129. آیا شما در رأی دهی سال 2006 انتخابات فدارل و ایالتی اشتراک کردید؟
130. بلی
131. نخیر

وظایف والدین /پدر و مادر بودن

(حالا ما چند سوال را در باره تربیه اولاد افغان که در بی ایریا کلان میشوند مطرح میکنیم)

(یک گروه سوالات را اول بکنید اگر مصاحبه شونده در حال حاضر طفل خود را بین سینین 5 تا 18 تربیه می کند)

1. آیا شما کدام زمانی معلمین طفل تان یا اولاد تان را به تماس شده اید که معلوم کنید که طفل تان در مکتب چطور پیش میرود؟
2. بلی، یکی دو بار
3. بلی، چندین بار
4. بلی، اکثرأ
5. نخیر
6. آیا شما و یا همسر تان حاضر شده اید که بطور افتخاری در مکتب طفل تان کمک کنید؟
7. بلی، اکثرأ
8. بلی، بعضی اوقات
9. بلی، یکی دو بار
10. نخیر
11. به چه اندازه شما و یا همسر تان در کار های خانگی اطفال تان کمک می کنید؟
12. هیچ وقت
13. بسیار کم
14. بعضی اوقات
15. مرتبأ
16. به کدام اندازه شما در مورد تاثیرات منفی شاگردان دیگر بالای اولاد تان در مکتب پریشان می باشید؟
17. قطعأ نی
18. کمی
19. تا اندازه ئی
20. بسیار زیاد
21. شما فکر میکنید که آیا خوب است که اولاد شما دوست غیر افغان داشته باشد؟
22. بلی
23. نخیر
24. یقین ندارم

حالا چند سوال د رمورد برنامه های اطفال مطرح می کنیم. این سوالات برای تمام مصاحبه شوندها است .

1. یک طفل را در سن سه ساله فکر کنید که هر دو پدر و مادر در جریان روز در کار هستند. کدام دو طریق ذیل نظم ونسق نگهداری طفل را شما فکر میکنید که بهتر باشد؟
2. یک کودکستان عمومی
3. یک کودکستان خصوصی/شخصی
4. یک کودکستان که در یک مسجد یا یک موسسه مذهبی موقیعت داشته باشد
5. پرستار داخل خانه
6. پرستار روزانه (بی بی ستر)
7. همسایه
8. یک عضو خانواده
9. دیگر وسیله____________________________________________
10. هیچکدام، یک طفل به سن سه باید توسط والدینش پرستاری شوند.
11. آیا شما در حال حاضر به یک کودکستان خوب و با کفایت ضرورت دارید اما توانمند نیستید که مصارف آنرا بدهید؟
12. بلی
13. نخیر
14. نمی دانم
15. به این سوال جواب داده نشد
16. جواب نبود
17. آرزو دارید که سیمینار ها و کورس های تربیوی در دسترس جوانان افغان باشد تا در مورد خطرات استفاده مواد مخدره و فروش آن آموزش بیبینند؟
18. نخیر، لازم نیست
19. یقین ندارم
20. بلی، این مطلب تا اندازه ئی مهم است
21. بلی، این مطلب بسیار کمک می کند
22. نمی دانم
23. به این سوال جولب داده نشد
24. اگر کورس های تربیوی و سیمینار ها در مورد خطرات استفاده و فروش مواد مخدره دائر گردد ، شما میخواهید که از نظر شریعت اسلامی درس داده شود؟
25. نخیر
26. یقین ندارم
27. بلی، این دیدگاه را باید شامل کنند
28. بلی، باید اساسأ از همین دیدگاه تدریس شود
29. نمی دانم
30. به این سوال جواب داده نشد
31. آرزو دارید که برنامه های در تلویزیون افغانها باشد که مرکز توجه را برای تعلیم و تربیه جوانان در امریکا تمرکز دهد؟
32. بلی، بسیار علاقمند هستم
33. بلی، تا اندازه ئی
34. نخیر.
35. (اگر مصاحبه شونده یک طفل دارد که از شانزده سال پائین است)

چه قدر برای شما مهم است و بوده میتواند که اولاد شما به زبان دری فصیح و روان بخواند و بنویسد؟

1. بسیار مهم نیست.
2. تا اندازه ئی مهم است
3. مهم است
4. بسیار مهم است
5. نمی دانم
6. به این سوال جواب داده نشد
7. جواب ندارد
8. (اگر مصاحبه شونده یک طفل دارد که از شانزده سال پائین است)

به چه اندازه برای شما مهم است و بوده میتواند که اولاد شما به زبان پشتو فصیح و روان بخواند و بنویسد؟

1. بسیار مهم نیست.
2. تا اندازه ئی مهم است
3. مهم است
4. بسیار مهم است
5. نمی دانم
6. به این سوال جواب داده نشد
7. جواب ندارد
8. شما آروز دارید که صنف های آموزش و پرورش زبان های دری و پشتو دائر گردد تا جوانان بتوانند خواندن ، نوشتن و مهارت زبانی خویش را تقویه کنند؟
9. بلی.
10. نخیر
11. یقین ندارم
12. نمی دانم
13. جواب داده نشد

(اگر مصاحبه شونده اولاد داشته باشد) در مورد اولاد شما نزدیک به سن 10 ، به چه اندازه او زبان های دری، پشتو و انگلیسی را سخن می گوید ، می نویسد ، و می خواند؟( اگر اولاد مصاحبه شونده همه بالغ هستند از جوان ترین ایشان پرسان شود)

| زبان | الف. سخن گفتن | ب. خواندن | ج. نوشتن |
| --- | --- | --- | --- |
| 1. دری/فارسی | 1. بسیار روان  2. متوسط  3. خوب است  4. یک کم  5. هیچ نی | . بسیار روان  2. متوسط  3. خوب است  4. یک کم  5. هیچ نی | 1. بسیار روان  2. متوسط  3. خوب است  4. یک کم  5. هیچ نی |
| 1. پشتو | 1. . بسیار روان  2. متوسط  3. خوب است  4. یک کم  5. هیچ نی | 1.. بسیار روان  2. متوسط  3. خوب است  4. یک کم  5. هیچ نی | 1 . بسیار روان  2. متوسط  3. خوب است  4. یک کم  5. هیچ نی |
| 1. انگلیسی | 1. . بسیار روان  2. متوسط  3. خوب است  4. یک کم  5. هیچ نی | 1. . بسیار روان  2. متوسط  3. خوب است  4. یک کم  5. هیچ نی | 1. . بسیار روان  2. متوسط  3. خوب است  4. یک کم  5. هیچ نی |

1. به چه قدر شما همرای اولاد تان در باره افغانستان صحبت می کنید؟
2. هیچ نی
3. بعضی اوقات
4. کمتر اوقات
5. بسیار اوقات

(سه سوال بعدی برای آنان است که در امریکا اولاد خود را تربیه کرده اند)

1. آیا شما پریشان هستید که اولاد شما ناکام ماند تا فرهنگ و هویت افغانی را حفظ کند؟
2. بلی، من در ین مورد بسیار پریشان هستم
3. بلی، من درین مورد تا اندازه ئی پریشان هستم
4. نخیر، این یک مشکل برای من نیست

(از مصاحبه شونده پرسیده شود که اگر اولادش از ده به بالا باشد و در مکاتب امریکائی شامل شده باشد)

1. آیا شما کدام زمانی همرای اولاد تان که درین کشور بزرگ شده این مشکل را داشته اید که بسیار امریکائی شده و یا فرهنگ افغانی خود را از دست داده است؟
2. بلی، اکثرأ
3. بلی، بعضی اوقات
4. بلی، بسیار کم
5. نخیر، هیچ مشکل نداشته ام
6. لاجواب اولاد ندارم
7. (پرسان کنید اگر مصاحبه شونده در امریکا پیش از سن 18 رسیده باشد) آیا شما همرای والدین خود مشکل دارید که فکر میکنند که شما بسیار امریکائی شده اید و یا فرهنگ افغانی را از دست داده اید؟
8. بلی، اکثرأ
9. بلی، بعضی اوقات
10. بلی، بسیار کم
11. نخیر، هیچ مشکل نداشته ام
12. اگر جواب بلی باشد، این مشکلات اکثرأ در باره چه است؟

_________________________________________________________________

(سوالات بعدی از همه افغانان بالغ پرسان شود)

1. والدین در مورد تربیه و دسپلین اولادنظریات مختلف دارند که چگونه اولاد شان اشخاص خوب تربیه شوند. آیا شما فکر میکنید که بسیاری از اولاد ها روش نیک را به جزا دادن از قبیل جیب خرچ شان را قطع کردن و منع کردن شان از بیرون رفتن و قید کردن بعضی چیزهای که دوست دارند، اشخاص خوب میشوند؟
2. بلی
3. نخیر
4. آیا شما فکر میکنید بهتر است که مطالب برای شان تشریح شود که چرا کاری را که میکنند غلط است و یا بهتر است که از ایشان اطاعت کنند بدون اینکه مطالب برای شان تشریح شود؟
5. تشریح شود که چرا کاری را که میکنند غلط است
6. فهمانده شوند که چطور اطاعت کنند بدون واضح ساختن موضوع
7. حالت دیگر_______________________________________
8. آیا شما فکر میکنید که بعضی اوقات والدین اطفال شانرا به نام های مختلف مانند احمق ، تنبل و نادان طعنه دهند که به راه راست روند و اشخاص خوب شوند؟
9. بلی، اکثرأ لازم است
10. بلی، بعضی اوقات لازم است
11. نخیر
12. آیا شما فکر می کنید که برای والدین ضرور است تا به پشت دست و پا اطفال شانرا بزنند تا اشخاص خوب شوند؟
13. بلی، اکثرأ لازم است
14. بلی، بعضی اوقات لازم است
15. نخیر
16. شما فکر میکنید که والدین ضرور است تا لت و کوب کنند تا اشخاص خوب شوند؟
17. . بلی، اکثرأ لازم است
18. بلی، بعضی اوقات لازم است
19. نخیر
20. آیا وجهه های در اجتماع امریکائی است که شما فکر میکنید که اولاد افغان باید آنرا جذب کنند و یا از آن استفاده برند؟
21. بلی.
22. نخیر
23. اگر جواب بلی باشد کدام وجهه اجتماع امریکائی است که شما فکر میکنید که اولاد شما آنرا به خود جذب کند و از آن استفاده ببرد؟ ( مصاحبه شونده ممکن اضافه تر از دو وجهه را ذکر نماید در آن صورت دو مهم ترین آن را انتخاب کنید)

_________________________________________________________________

_________________________________________________________________

1. آیا به نظر شما بعضی چیز های مخرب و بیراه کننده در اجتماع امریکائی است که اولاد شما از آن حذر کند؟
2. بلی
3. نخیر
4. اگر جواب بلی است ، لطفأ بگوید کدام وجهه فرهنگ امریکا مهمتر است که اطفال افغان از آن اجتناب کنند؟ ( مصاحبه شونده ممکن اضافه تر از دو وجهه را ذکر نماید در آن صورت دو مهم ترین آن را انتخاب کنید) _________________________________________________________

__________________________________________________________________________________________________________________________________

1. به نظر شما آیا والدین افغان اجازه دهند تا جوانان شان با هم سن وسالان شان با دختر و بچه یکجا برای تفریح و ساعت تیری در سینما و دیگر محافل بیرون بروند؟
2. بلی
3. نخیر

امور صحی و سلامتی

1. آیا یک جای مشخص است که شما عمومأ برای علاج و یا مشوره طبی در مورد صحت تان می روید؟
2. بلی
3. نخیر
4. زیاد تر از یک جا
5. جواب داده نشد
6. نمی دانم
7. به کدام محل زیاد تر شما اکثرأ می روید ؟ کلینیک ، دفتر داکتر ، سرویس عاجل و یا جای دیگر؟
8. معاینه خانه داکتر
9. مرکز تداوی قراردادی
10. سرویس عاجل یک شفاخانه
11. شعبه معاینه خانه یک شفاخانه
12. کلینیک صحی
13. علاج و تداوی های یونانی. چه نوع___________________________
14. جای دیگر:__________________________________________
15. در یک جای اکثرأ نمی روم
16. نمی دانم
17. جواب داده نشد
18. آیا شما کدام نوع بیمه صحی دارید مانند پلان های گروپی ، حکومتی، مدیکل،مدیکیر؟
19. بلی
20. نخیر(از سوال بعدی بگذرید)
21. نمیدانم (از سوال بعدی بگذرید)
22. جواب رد (از سوال بعدی بگذرید)
23. چه نوع بیمه صحی شما دارید؟ هر کدام را در فهرست می توانید خط بکشید
24. پلان گروهی از طرف کار
25. بیمه صحی خصوصی
26. مدیکیر
27. مدیکل
28. پلان های کمکی ( برای نسخه ، درازا مدت و غیره)
29. دیگر نوع________________________
30. نمی دانم
31. آیا شما بیمه صحی دارید که مصارف دندان های شما را احتوا کند؟
    1. بلی
    2. نخیر
32. در دوازده ماه گذشته ، کدام وقتی بود که شما بیمه صحی نداشته بوده باشید؟
33. بلی
34. نخیر
35. نمی دانم
36. در جریان دوازده ماه گذشته ، چنین وقت را سر خورده اید که شما احتیاج داشته بوده باشید که یک داکتر را بیبینید اما نتوانستید به خاطر مصارف پولی آن؟
37. بلی
38. نخیر
39. نمی دانم
40. چقدر وقت میشود از وقتیکه شما یک داکتر را برای معاینات صحی عمومی دیده باشید؟
41. در ظرف یکسال گذشته ( کمتر از دوازده ماه پیش)
42. در ظرف دو سال گذشته ( یکسال اما کمتر از دوسال پیش)
43. در ظرف پینج سال گذشته( دو سال اما کمتر از پینج سال پیش)
44. پینج یا زیاد تر از سال پیش
45. چقدر وقت میشود که شما یک داکتر دندان را دیده اید؟
46. .در ظرف یکسال گذشته ( کمتر از دوازده ماه پیش)
47. در ظرف دو سال گذشته ( یکسال اما کمتر از دوسال پیش)
48. در ظرف پینج سال گذشته( دو سال اما کمتر از پینج سال پیش)
49. پینج یا زیاد تر از سال پیش
50. چطور وضع صحی تانرا ارزیابی میکنید؟
51. فوق العاده
52. بسیار خوب
53. خوب
54. جور است
55. خراب
56. نمی دانم
57. آیا یک متخصص روانی یا داکتر معالج شما را به امراض ذیل تشخیص کرده است؟ (تمام امراض تشخیص شده را انتخاب کنید)

الف. تکلیف قلبی د. حمله مغزی

ب. شکر ذ. توبرکلوز

ب. مرگی ر. تایراید

پ. هیپیتاتس ز. بای پولر

ت. اس تی دی ژ. افسردگی/ غمباده

ث. اچ ای وی س. ناآرامی های فکری

ج. هایپرتنشن (تشنج) ش. افسردگی های بعد از واهمه

چ. مرض گرده ص. زایفرینه

ح. تکلیف جگر ض. سرطان

خ. تکلیف شش

1. اگرمرض سرطان تشخیص شده باشد ، چه نوع سرطان است؟ _______________________

آیا کدام داکتر و یا شفاخانه هرگز شما را به یک مرض مدهش طبی و یا روانی تشخیص کرده است؟

1. تشخیص شماره اول________________________________________
2. تشخیص شماره دوم________________________________________
3. تشخیص شماره سوم_______________________________________
4. آیا شما کدام درد مضمن دارید مثل سر دردی ، درد در قفس سینه که داکتران تا حال نتوانستند که تشخیص کنند؟
5. بلی
6. نخیر

ورزش و تمرین:

چه نوع ورزش را شما در ماه که گذشت کرده اید؟ لطفأ نام بگیرید

1. الف. هر نوع سپورت و ورزش که شما در جریان ماه گذشته شرکت کرده اید
2. تقریبا چند روز هفته را شما تمرین کرده اید؟
3. تقریبا وسط چند دقیقه را در هر تمرین سپری کرده اید؟

(کود پائین را استفاده کنید. اگر کود داده نشده ، نام سپورت و یا فعالیت بدنی را بگیرید)

| نوع فعالیت بدنی یا ورزش | چند روز در هفته | چند دقیقه در هر بار |
| --- | --- | --- |
| 1. الف. | ب. | ج. |
| 1. الف. | ب. | ج. |
| 1. الف. | ب. | ج. |
| 1. الف. | ب. | ج. |
| 1. الف. | ب. | ج. |

1. ایروبیک 11.جاگنگ 21. آب بازی
2. باسکتبال 12.قایق رانی 22.تای چی
3. بایسکل 13.کاراته و ژودو 23. تینس
4. بولنگ 14. راکتبال 24. سر تسمه دویدن
5. باکسنگ 15. رولربلیدینگ 25. والیبال
6. فوتبال امریکائی 16.چمچه زنی قایقی 26. قدم زدن
7. باغبانی 17. دویدن 27. وزن برداری
8. کوه گردی 18.سکیتنگ 28. کشتی
9. گالف 19. فوتبال 29. یوگا
10. هاکی 20. زینه بالاشدن 30. دیگر 1___________

31. دیگر 2___________

1. آیا شما در برنامه های ورزشی اشتراک میکنید و یا آیا شما عضو کدام کلب ورزشی و یا صحی هستید؟
2. بلی(دو سوال بعدی را بگذارید)
3. نخیر
4. اگر جواب نی است آیا برنامه های ورزشی در کمونیتی شما وجود دارد که بتوانید مصارف آنرا پوره کنید و احتیاجات شما برآورده شود؟
5. بلی
6. نخیر
7. یقین ندارم
8. آیا شما علاقمند هستید که در برنامه های ورزشی حصه بگیرید اگر یکی در دسترس و نزدیکی شما باشد که احتیاج شما را رفع سازد؟
9. بلی
10. نخیر
11. آیا شما از خوردن غذا های شحمی و چربی دار جلوگیری می کنید و یا کمتر میخورید؟
12. بلی
13. نخیر
14. آیا شما از خوردن غذا های نمکی خود داری می کنید و یا کمتر میخورید؟
15. بلی
16. نخیر
17. آیا شما از خوردن غذا های شیرین و شکری و شربت های شیرین جلوگیری می کنید یا کمتر میخورید؟
18. بلی
19. نخیر
20. آیا شما عمومأ حبوبات، سیریال ، نان خشک و برنج می خورید؟
21. بلی
22. نخیر
23. به چه اندازه شما از غذا های مکدانلد ، برگرکنگ و پیتزه و غیره استفاده می کنید؟
24. هیچ وقت یا به ندرت
25. بعضی اوقات ، کمتر از یک بار در هفته
26. یک یا دو بار در هفته
27. سه یا چهار بار در هفته
28. پینج تا شش یار در هفته
29. زیاد تر از هفت بار در هفته
30. آیا شما علاقه دارید که در مورد تغذیه غذائی و رژیم غذائی صحی معلومات زیاد تر حاصل کنید؟
31. بلی، بطور یقین
32. بلی، تا اندازه ئی
33. نخیر

سوالات بعدی در مورد نفوذ طرز مکالمه و صحبت با داکتر است که تاثیر بارز دارد. سوال های اولی در مورد دیدار با داکتر شما در دو سال اخیر می باشد.

1. به کدام اندازه داکتر های را که برای تداوی میروید زبان را صحبت می کنند که شما به خوبی فهمیده نمی توانید؟ (کارت را نشان دهید____)
2. هرگز
3. بعضی اوقات ( کمتر از یک بار در چهار دیدار)
4. بعضی اوقات ( ¼ تقریبأ تا ½ دیدار که با داکتر می کنم)
5. اکثرأ ( ½ تا تقریبأ ¾ همه دیدار های که با داکتر می کنم)
6. اکثر اوقات ( ¾ تا تقریبأ همه دیدار های که با داکتر می کنم)
7. همیشه
8. جواب ندارد
9. نمی دانم
10. این سوال برای جواب رد شد
11. در جریان دیدار های شما با داکتر در دو سال اخیر آیا یک ترجمان حاضر بود که برای شما گفتارداکتر را ترجمه کند و شما بفهمید که چه می گوید یا شما را کمک کرده باشد و به داکتر مشکل شما را تشریح کند؟
12. بلی
13. نخیر( سه سوال بعدی را بگذارید)
14. اگر جواب بلی است ، کی برای شما ترجمه کرده است؟
15. یکی از اولاد هایم
16. همسرم
17. یک خویشاوندم
18. دوست
19. ترجمان مسلکی
20. کسی دیگر: مشخص کنید______________________
21. جواب ندارد
22. نمی دانم
23. این سوال برای جواب رد شد
24. (اگر جواب دهنده زیاد تر از یکی را جواب گفته است) کدام یکی را که شما انتخاب کردید شما را زیاد تر کمک کرده است؟
25. یکی از اولاد هایم (سوال بعدی را بگذرید)
26. همسرم (سوال بعدی را بگذرید)
27. دیگر اقارب (سوال بعدی را بگذرید)
28. دوست (سوال بعدی را بگذرید)
29. یک ترجمان مسلکی
30. کس دیگر (سوال بعدی را بگذرید)
31. جواب ندارد
32. نمی دانم
33. این سوال برای جواب رد شد
34. (اگر جواب دهنده به غیر از ترجمان مسلکی جواب داده) آیا شما فکر میکنید که یک ترجمان مسلکی شما را خوبتر کمک می کند که داکتر تانرا بفهمید به جای افراد که در سوال قبلی تذکر رفت؟
35. بلی
36. نخیر
37. یکی است
38. جواب ندارد
39. نمی دانم
40. این سوال برای جواب رد شد
41. اینطور یک وقتی بوده که در دو سال اخیر که شما به یک ترجمان برای داکتر ضرورت داشته باشید اما هیچکس قابل دسترس نبوده باشد؟
42. بلی
43. نخیر
44. نمی دانم
45. این سوال برای جواب رد شد
46. اگر جواب بلی است ، چند بار همچو یک چیز اتفاق افتاده که شما به ترجمان احتیاج داشته باشید و کسی به دسترس نبوده؟
47. به ندرت ( یکی دو بار)
48. گاهگاهی ( کمتر از یک بار در چهار ویزیت)
49. بعضی وقت( ¼ تا تقریبأ ½ ویزیت داکترم صورت گرفته)
50. اکثرأ (½ تا تقریبأ ¾ ویزیت داکترم صورت گرفته)
51. اکثر اوقات ( ¾ تمام ویزیت های داکترم صورت گرفته)
52. جواب ندارد
53. نمی دانم
54. این سوال برای جواب رد شد
55. آیا شما کدام وقت تقاضای یک ترجمان را وقتیکه پیش داکتر رفته اید از داکتر و یا موسسه صحی مربوط کرده اید؟
56. بلی (سوال بعدی را بگذرید)
57. نخیر
58. جواب ندارد
59. نمی دانم
60. این سوال برای جواب رد شد
61. چند بار یک ترجمان برای شما فراهم گردید وقتیکه شما درخواست کردید؟
62. هیچ وقت (سوال بعدی را بگذرید)
63. به ندرت
64. بعضی وقت
65. عمومأ
66. همیشه
67. جواب ندارد
68. نمی دانم
69. این سوال برای جواب رد شد
70. اگر شما و یا یکی از اعضای خانواده شما برای معاینات صحی به یک ترجمان ضرورت داشته باشید و کسی از اعضای خانواده و یا دوستان نتوانند شما را کمک کنند ، ایا شما فکر میکنید که این حق قانونی شماست که باید کمک شوید؟
71. بلی
72. نخیر
73. یقین ندارم
74. جواب ندارد
75. نمی دانم
76. این سوال برای جواب رد شد.
77. چه قدر شما مشکل دارید که بفهمید که داکتر تان در باره صحت شما و یا علاج آن چه می گوید؟
78. هرگز
79. به ندرت
80. بعضی وقت
81. عمومأ
82. آیا کدام وقت شما احساس کردید که داکتر به شما بی احترام بوده باشد؟
83. بلی
84. نخیر (سوال بعدی را بگذرید)
85. جواب ندارد
86. نمی دانم
87. این سوال برای جواب رد شد
88. اکثرأ این بی احترامی اتفاق می افتد؟
89. به ندرت
90. بعضی وقت
91. عمومأ
92. جواب ندارد
93. نمی دانم
94. این سوال برای جواب رد شد
95. شما احساس می کنید که داکتر به سخنان شما به دقت گوش نمی دهد یا بی تفاوت است که شما چه می گوید وقتیکه مشکل صحی خود را برایش تشریح میکنید؟
96. بلی
97. نخیر (از سوال بعدی بگذرید)
98. چه قدر این بی تفاوتی داکترکه به شما گوش نمی دهد، اتفاق می افتد؟
99. به ندرت
100. بعضی اوقات
101. عمومأ
102. جواب ندارد
103. نمی دانم
104. این سوال برای جواب رد شد
105. آیا گاهی داکتر به شما گفته است که شما دیگر علاج شده اید و اما شما هنوز هم مریض هستید؟
106. بلی
107. نخیر
108. جدال و منازعات خانوادگی بالای صحت شخص تاثیر بارز دارد. اگر یک زن و یا یک مرد به شکل دوام دار از طرف همسر خود لت و کوب شود ، به کی باید رجوع کند؟
109. اعضای خانواده
110. بزرگان کمونیتی
111. دوستان اعتمادی
112. امام یا رئیس مسجد
113. مشاور فرهنگی
114. مشاور مذهبی در فامیل
115. پولیس
116. یک مرجع پناهندگان منازعات خانوادگی و یا تیلفون همیشگی آنها
117. دیگر کس و یا مرجع______________________________
118. با در نظر داشت خانواده های افغان که شما می شناسید، چند نفر آنها در دو سال گذشته مشکلات خانوادگی داشته اند که به لت و کوب و دهشت انجامیده است؟
119. هیچ یک
120. بسیار کم
121. بعضی(یک نفر در چهار خانواده)
122. بسیار ( زیاد تر از یک نفر در چهار خانواده)
123. بسیار زیاد( در حدود یک نفر در دو خانواده)
124. حد اکثر( در حدود سه نفر در چهار خانواده)

صحت و سلامی مردان:- سه سوال بعدی صرف برای مردها است

آیا معاینات ذیل را هرگز کرده اید؟

|  | بلی، در سال گذشته | بلی، در زیاد تر از دو سال گذشته | نخیر، هیچوقت | نمی دانم |
| --- | --- | --- | --- | --- |
| 1. تنهامردان سینین 18 تا 35: تیستکولر | 1 | 2 | 3 | 99 |
| 1. مردان که از سن پینجاه به با لا باشند. معاینه پراستیت | 1 | 2 | 3 | 99 |
| 1. مردان که از سن پینجاه به با لا باشند. معاینه سرطان روده | 1 | 2 | 3 | 99 |

صحت و سلامتی زنان (سوالات بعدی تنها برای زنان است)

1. برعلاوه دیدن داکتر همیشگی تان آیا شما یک داکتر نسائی را می بینید یا نه ؟
2. بلی
3. نخیر (سوال بعدی را بگذارید)
4. آیا داکتر نسائی شما زن است یا مرد؟
5. مرد
6. زن
7. چند بار شما حامله شده اید؟ لطفأ همه حالات حاملگی را به شمول حاملگی فعلی اگر حامله هستید ، وضع حمل کرده باشید و یا سقط کرده باشید و یا طفل تان ضایع شده باشد تذکر دهید___________

(اگر هیچ وقت حامله نشده باشد چهار سوال بعدی را تیر شوید)

1. درکدام مقده/وقت خاص حاملگی شما نتیجه مثبت داده یعنی طفل سالم تولد شده است؟_________
2. در کدام مقطع حاملگی تان شما یک غم خورشی قبل از زایمان داشته اید؟
3. در شروع حاملگی
4. چند ماه محدود به حاملگی
5. نزدیک به وضع حمل
6. من خواستار غم خورشی در حاملگی نشده ام
7. آیا شما فعلا و یا در گذشته به طفل تان از پستان شیر داده اید؟
8. بلی، همه اولا هایم را (سوال بعدی را بگذارید)
9. بلی، بعضی از اولاد ها را
10. نخیر
11. اگر شما شیر خود را نداده اید دلایل تان درین مورد چه بوده است؟
12. مشکلات کار و تقسیم اوقات روزانه
13. مشکلات فزیکی و صحی مادر
14. مشکلات فزیکی و صحی طفل
15. طفل به شیر بوتل علاقه نشان میداد
16. من نمی دانستم که چطور از شیر خود طفل را تغذیه کنم
17. دلیل دیگر________________________________

آیا کدام وقت معاینات ذیل را گرفته اید؟

|  | بلی، در یک سال گذشته | بلی، ما در سال گذشته نی | نخیر، هیچ وقت | من نمی دانم |
| --- | --- | --- | --- | --- |
| 1. معاینات فنی سینه در کلینیک | 1 | 2 | 3 | 99 |
| 1. پپ سمیر | 1 | 2 | 3 | 99 |
| 1. زنان بالاتر از سن چهل: مموگرام | 1 | 2 | 3 | 99 |
| 1. زنان بالاتر از سن پینجاه: معاینه سرطان روده | 1 | 2 | 3 | 99 |

1. آیا شما خود تان سینه تانرا معاینه می کنید؟
2. بلی، ماه یک بار
3. بلی، در هر 2-3 ماه
4. بلی، در هر 4-6 ماه
5. بلی در هر 7 الی دوازده ماه
6. بلی، کمتر از یکبار در سال
7. نخیر
8. به کدام اندازه شما در مورد امراض استخوانی و پودگی استخوان ( آستیو پروسیس) معلومات دارید؟
9. خوب آشنائی دارم
10. کمی معلومات دارم
11. آشنائی زیاد ندارم
12. هیچ معلومات ندارم
13. آیا شما از دوا های ضد حاملگی در سال گذشته استفاده کرده اید؟
14. بلی
15. نخیر (سوال بعدی را بگذارید)
16. جواب این سوال رد شد
17. چه نوع طریقه جلوگیری از حاملگی را شما استفاده میکنید؟
18. پیچکاری
19. نارپلنت (هورمونی)
20. تابلیت
21. پچ
22. پوش (کاندوم)
23. آله انتراتورین
24. دایه فرام
25. عزل
26. سپرمیسید
27. دیگر طریق_______________
28. جواب این سوال رد شد
29. به چه اندازه شما در جلوگیری از حاملگی تان متیقین می باشید؟
30. همیشه ( صد در صد)
31. زیاد تر وقت ( نود در صد)
32. اکثرأ ( 75 در صد)
33. بعضی اوقات ( 50 در صد)
34. گاهگاهی ( 25 در صد)
35. جواب این سوال رد شد.
36. آیا شوهر شما در جلوگیری از حاملگی با شما موافق است؟
37. بلی
38. نخیر
39. شوهرم خبر ندارد که من وسیله ضد حاملگی را استفاده می کنم
40. جواب این سوال رد شد
41. آیا شما آروز دارید که در مورد طرق ضد حاملگی از یک شخص مسلکی بیاموزید؟
42. بلی
43. نخیر

جدال و منازعات خانوادگی بالای صحت زنان تاثیر بارز دارد. آیا شما یکی از موارد ذیل را در زندگی تان تجربه کرده اید؟

1. اهانت زبانی ( مانند طعنه دادن ، سوگند خوردن به یک کار در مقابل شما و کم زدن)
2. بلی
3. نخیر
4. اگر جواب بلی باشد ، کی این کا ر در مقابل شما کرده است؟
5. همسر
6. والدین
7. والدین همسر
8. برادر و یا خواهر
9. دختر و پسر
10. دیگر اقارب______________________
11. اهانت جسمی ( مانند تیله کردن، زدن ، خفه کردن و یا زدن همرای یک شی جارحه)
12. بلی
13. نخیر
14. اگر جواب بلی باشد کی این کار را در مقابل شما کرده است؟
15. همسر
16. والدین
17. والدین همسر
18. برادر و یا خواهر
19. دختر و پسر
20. دیگر اقارب______________________
21. چند بار این کار اتفاق افتاده
22. چندین بار در هفته
23. چند بار در ماه
24. چند بار در سال
25. بک یا دو بار در سال
26. یک بار اتفاق افتاده
27. ایا این موضوع را با یک داکتر و یا شخص فنی طبی صحبت کرده اید؟
28. بلی
29. نخیر.

حالا من قصه دو نفررا دارم و که چگونه زندگی شان به پیش میرود. این دو قصه در باره اشخاص حقیقی نیستند اما مردمی است که مثل همین قصه را دارند. بعد از توضیح هر کدام من نظر شما را در باره موقف شان پرسان می کنم.

الف. ولی یک مرد افغان است که در فریمانت زندگی میکند. در دو هفته آخر ولی بسیارجگر خون است. از خواب که میخیزد ولی خود را دل تنگ احساس میکند و تمام روز هم دل تنگ است. مثل همیش از هیچ کار لذت نمی برد. اصلاً، هیچ چیز برایش خوشی نمیدهد. حتی وقت که یک چیز خوب میشود، ان وقت هم ولی خوشحال نمی شود. روزها را به سختی تیر میکند. خوردترین وظیفه ها را به مشکل انجام میدهد. تمرکز کردن برای اش بسیار مشکل شده. خود را بی انرجی و بی اندازه خسته احساس می کند. با این که خود را خسته احساس میکند، شب که می شود، ولی را خواب نمی برد. ولی خود را بی ارزش و دل سرد احساس می کند. خانواده ای ولی متوجه شدن که ولی هر وقت در این یک ماه نیست و خودش را از آن ها دور گرفته. خلسش که ولی هیچ نمیخواهد که گپ بزند.

1. به نظر شما ، مشکل ولی چه نامیده میشود؟ نامش چیست؟_____________________________
2. به نظر شما ، چه فکر میکنید که چه باعث مشکل ولی شده باشد؟ ( مصاحبه شونده شاید بگوید که نمی داند و متیقن نیست پس پرسان کنید که به تصور او نظرش چه است؟

_________________________________________________________________

1. به نظر شما به چه اندازه مشکل ولی جدی است؟
2. بسیار جدی
3. تا اندازه ئی جدی
4. بسیار جدی نیست
5. هیچ جدی نیست
6. نمی دانم
7. این سوال رد شد
8. به نظر شما ولی باید به خاطر این حالت خواستار کمک شود؟
9. بلی
10. نخیر
11. نمی دانم
12. این سوال رد شد
13. اگر جواب بلی است ، ولی از کی کمک بگیرند؟_____________________________
14. چگونه میتواند که کمک شوند______________________________________________
15. آیا کسی دیگر است که ولی از ایشان کمک بگیرد؟_____________________________
16. چگونه میتواند که کمک شوند______________________________________________
17. به نظر شما چه نتیجه خواهد تا وقتیکه ولی کمک شود؟__________________________
18. به نظر شما ، چقدر امکان دارد که ولی نشیب و فراز زندگی را تجربه می کنند؟
19. بسیار امکان دارد
20. تا اندازه ئی امکان دارد
21. بسیار امکان کم دارد
22. قطعأ امکان ندارد
23. نمی دانم
24. سوال رد شد
25. به نظر شما، به چه اندازه امکان دارد که ولی یک مشکل روانی داشته باشند؟
26. بسیار امکان دارد
27. تا اندازه ئی امکان دارد
28. بسیار امکان کم دارد
29. قطعأ امکان ندارد
30. نمی دانم
31. سوال رد شد
32. به نظر شما ، به کدام اندازه ممکن است که ولی یک مریضی جسمی را تجربه کنند؟
33. بسیار امکان دارد
34. تا اندازه ئی امکان دارد
35. بسیار امکان کم دارد
36. قطعأ امکان ندارد
37. نمی دانم
38. سوال رد شد
39. اینجا یک فهرست مطالب را داریم که ولی و سحر اجرا کنند که اگر شود تا کمک شوند. کدام یک ازین فهرست شما فکر میکنید که ولی و سحر باید اجرا کنند؟
40. در مورد همرای خانواده سخن گویند؟
41. از یک امام و یا شخصیت دینی کمک گیرند؟
42. کوشش کنند تا پینج وقت نماز و حتی نفل روزانه ادا کنند؟
43. از یک داکتر طبی کمک گیرند؟
44. پیش یک مشاور روانی و یا اشخاص مسلکی دیگر بروند؟
45. پیش یک شخصیت دینی برای دم و دعا بروند مانند سید ؟
46. پیش یک کسی بروند که کمک کند و جادو را رد کند؟
47. شامل یک گروه شود که آنها عین مشکل را دارند تا همدیگر را کمک کنند؟
48. بعضی دوا ها را از بازار خریداری کند که نسخه ضرور نیست مانند دوای خواب؟
49. از نزد داکتر دوا بگیرد؟
50. دوای یونانی بگیرد؟
51. خود را آرام کند و به یک رخصتی برود؟
52. آیا ولی ازمشکل خود به رفقای خود بگوید؟
53. حتمأ
54. اغلبأ
55. فکر نمی کنم
56. قطعأ نی
57. نمی دانم
58. به این سوال جواب رد داده شد
59. به شناسائی که از افغانان دارید ، وقتیکه از وضعیت ولی خبر شوند ، چه فکر میکنند؟

_________________________________________________________________

_________________________________________________________________

1. با شناسائی که از افغانان دارید، به چه اندازه میخواهند که همرای ولی دوست شوند؟
2. یقینأ میخواهند
3. اغلبأ میخواهند
4. فکر نمی کنم بخواهند
5. قطعأ نمی خواهند
6. نمی دانم
7. به این سوال جواب رد داده شد

قصه دوم

ب. سحریک دختر 14 ساله افغان است که در فریمانت زندگی میکنند. درچند ماه گذشته سحر خیلی بد خلق بوده. بعد از مکتب در اطاقش میمانه و دیگر به دوست هایش و سرگرمی هایش علاقه نشان نمیدهد. سحر میگه که خیلی خسته است، با این که بیشتر از همیش میخوابه و غذا ا هم نمی خورد. سحر به سختی روی کارهای خانه و مکتب تمرکز میکند و به پدر و مادرش گفته "کاش که به دنیا نمیامدم." یکی از دوست هایش شنیده که سحر میخواهد خودکشی کند.

1. به نظر شما ، مشکل سحر چه نامیده میشود؟ نامش چیست؟_____________________________

_________________________________________________________________

1. به نظر شما ، چه فکر میکنید که چه باعث مشکل سحر شده باشد؟ ( مصاحبه شونده شاید بگوید که نمی داند و متیقن نیست پس پرسان کنید که به تصور او نظرش چه است؟

_________________________________________________________________

_________________________________________________________________

1. به نظر شما به چه اندازه مشکل سحر جدی است؟
2. بسیار جدی
3. تا اندازه ئی جدی
4. بسیار جدی نیست
5. هیچ جدی نیست
6. نمی دانم
7. این سوال رد شد
8. به نظر شما سحر باید به خاطر این حالت خواستار کمک شوند؟
9. بلی
10. نخیر
11. نمی دانم
12. این سوال رد شد
13. اگر جواب بلی است ، سحر از کی کمک بگیرند؟_____________________________
14. چگونه میتواند که کمک شوند______________________________________________

_________________________________________________________________

1. آیا کسی دیگر است که سحر از ایشان کمک بگیرد؟_________________________________

_________________________________________________________________

1. چگونه میتواند که کمک شوند______________________________________________

_________________________________________________________________

1. به نظر شما چه نتیجه خواهد داد وقتیکه سحر کمک شوند؟ ____________________________

_________________________________________________________________

1. به نظر شما ، چقدر امکان دارد که سحر نشیب و فراز/ پستی و بلندی زندگی را تجربه می کنند؟
2. بسیار امکان دارد
3. تا اندازه ئی امکان دارد
4. بسیار امکان کم دارد
5. قطعأ امکان ندارد
6. نمی دانم
7. سوال رد شد
8. به نظر شما، به چه اندازه امکان دارد که سحر یک مشکل روانی داشته باشند؟
9. بسیار امکان دارد
10. تا اندازه ئی امکان دارد
11. بسیار امکان کم دارد
12. قطعأ امکان ندارد
13. نمی دانم
14. سوال رد شد
15. به نظر شما ، به کدام اندازه ممکن است که سحر/ولی یک مریضی جسمی را تجربه کنند؟
16. بسیار امکان دارد
17. تا اندازه ئی امکان دارد
18. بسیار امکان کم دارد
19. قطعأ امکان ندارد
20. نمی دانم
21. سوال رد شد

فهرست مشکلات و شکایت های افغانان را وقتیکه تحت فشار می باشند درین جا تقدیم می کنیم. لطفأ بگوید که به کدام اندازه این مشکلات در جریان ماه اخیر به شمول امروز باعث ناراحتی شما شده است؟

صفر= قطعأ 1= یک کمی 2. متوسط 3. یک کمی زیاد 4. بسیار زیاد

1. ____ مشکل به یاد آوردن مطالب و چیز های روزمره
2. ____ به آسانی متاثر شدن و دلگیر شدن
3. ____ درد داشتن در قلب و سینه
4. ____ چنین احساس داشتن که اکثر مردم نمی تواند اعتماد شود
5. ____ عصبی شدن که شما نمی توانید آنرا اداره کنید
6. ____ احساس تنهائی کردن وقتیکه حتی شما در بین مردم باشید
7. ____ احساس این را داشتن که شما به زودی و آسانی آزرده میشوید
8. ____ احساس این را داشتن که مردم احساس دوستی و رفاقت ندارند و شما را خوش ندارند.
9. ____ مشکل تصمیم گرفتن
10. ____ اکثرأ داخل جنگ و دعوا شدن
11. ____ دیگران کار های شما را به درستی قدر نمی کنند و موفقیت های شما را نمی شناسند
12. ____ احساس بی ارزش و بیهودگی کردن
13. ____ احساس گناه کردن

حالا فهرست بعضی مشکلات دیگر را که مردم تحت فشار هستند تقدیم می کنیم. لطفأ تذکر دهید که به چه اندازه در ماه گذشته این حالات را تجربه کرده اید؟

صفر= هرگز 1= تقریبأ هیچ تجربه نکردم 2. بعضی وقت 3. اکثرأ 4. به کثرت

1. ____ در جریان ماه گذشته، به چه اندازه شما احساس ترس کردید و یا احساس وارخطائی به شما رخ داده؟
2. ____ در جریان ماه گذشته، به چه اندازه شما حس کردید که جگر خون هستید؟
3. ____ در جریان ماه گذشته، به چه اندازه شما حس کردید که تاثرات و غمبادگی شما را تکلیف میدهد؟
4. ____ در جریان ماه گذشته، به چه اندازه شما حس کردید که عصبی هستید و یا تشنج دارید؟
5. ____ در جریان ماه گذشته، به چه اندازه شما حس کردید که سر دردی دارید؟
6. ____ در جریان ماه گذشته، به چه اندازه شما وقت تانرا به چرت و فکر تیر کردید؟
7. _____در جریان ماه گذشته، به چه اندازه شما حس کردید که دلتنگ هستید؟
8. _____ در جریان ماه گذشته، به چه اندازه شما حس کردید که زندگی شما بیهوده است؟
9. ____ در جریان ماه گذشته، به چه اندازه شما حس کردید که دلهره دارید؟
10. ____ در جریان ماه گذشته، به چه اندازه شما عصبی گردیده اید؟
11. ____ در جریان ماه گذشته، به چه اندازه شما حس کردید که هیچ چیز به دل شما نمی شود قسمیکه شما آرزو دارید؟
12. ____ در جریان ماه گذشته، به چه اندازه شما حس کردید که در مورد همه چیز ناامید هستید؟
13. ____ در جریان ماه گذشته، به چه اندازه شما حس کردید که بیچاره هستید؟

(به مصاحبه شونده یاد آوری کنید که این مشکلات را مردم دارند وقتیکه زیر فشار شدید قرار دارند)

1. ____ در دو هفته گذشته چند بار حس کردید که فشار شما بالا و یا پایان است؟
2. ____ در جریان ماه گذشته، به چه اندازه شما اوقاتی بوده که وقتیکه شما نه به خود و نه به دیگران کمک کرده توانستید ، و سرگیچ بودید که همه چیز دیگر بی ارزش است و در زندگی معنی ندارد.
3. ____ در جریان ماه گذشته، به چه اندازه شما مشکل داشتید که بالای یک موضوع توجه نماید و یا حواس تانرا جمع کنید؟
4. ____ در جریان ماه گذشته، به چه اندازه شما خود را به دست خود زده اید و یا زخمی کردید؟
5. ____ در جریان ماه گذشته، به چه اندازه شما شما کسی دیگر را در خانواده اهانت کرده اید و یاد زده اید؟
6. ____به طور عموم، به کدام اندازه شما در سال که گذشت با خود خوش بودید ؟
7. (صفر) بسیار خوش بودم
8. تا اندازه ئی خوش بودم
9. تا اندازه ئی نا راضی بودم
10. بسیار ناخوش بودم

لطفأ عکس العمل های ذیل را که بعضی اوقات حتی بعد از یک دوره پریشانی و وسواس و یا زندگی تهدید آمیز اتفاق افتاده مد نظر بگیرید. این سوالات در مورد عکس العمل های شخصی در یک زندگی پر آشوب و تهدید آمیز است که شما تجربه کرده اید. لطفأ از تجربه خود اقلأ دو بار در دو هفته اخیر بلی و یا نخیر جواب بدهید ولو که تجربه نکرده باشد. ( بلی=1 ، نخیر=2)

1. ___افکار و خاطرات ناراحت کننده در باره یک حادثه و برعلیه خواهش شما در مغز شما خطور کرده باشد.
2. ____ خواب های ناراحت کننده در باره یک حادثه.
3. ____ احساس اینکه چنین فکر کنید که یک حادثه تکرار اتفاق افتد.
4. ____ احساس جگرخونی با یاد آوری یک حادثه پر آشوب.
5. ____ عکس العمل های جسمی (مانند پرش قلب ، درد معده ، عرق ریزی ، سرچرخی،) وقتیکه یک حادثه را به یاد می آورید.
6. ____ مشکل داشتن به خواب رفتن و ناآرامی در خواب.
7. ____ داشتن همچو احساس قوی ارتکاب یک عمل مخرب به خود تان و دیگران.
8. ____ در حالت بسیار متشنج/وارخطایی در یک موضوع غیر مترقبه.

بالاخره چند سوال در باره "کابل کوچک" داریم. نظر شما را میخواهیم

1. نام "کابل کوچک" را شنیده اید؟
2. بلی
3. نخیر
4. "کابل کوچک" چه است؟

______________________________________________________________________

______________________________________________________________________

1. آیا شما فکر میکنید که بدون از افغانها طرفدار و یا مخالف "کابل کوچک" هستند؟
2. طرفدار
3. بی تفاوت هستند
4. مخالف هستند
5. آیا شما انکشاف یک ساحه سیاحتی در شهر فریمانت را به نام "کابل کوچک" که نمایانگر فرهنگ افغانستان مانند هنر ، موسیقی ، تاریخ ، پخت و پز و مهمانوازی باشد حمایه و پشتیبانی میکنید؟
6. بلی
7. متیقن نیستم
8. نخیر

بدین وسیله از وقت گرانبهای شما که درین تحقیق سهم گرفتید یک جهان سپاسگزاریم.
