# Supplementary material for: Discrimination and distress among Afghan refugees in northern California: The moderating role of pre- and post-migration factors
Source: PLoS One. 2018 May 21;13(5):e0196822. doi: 10.1371/journal.pone.0196822 (PMC5962064; doi:10.1371/journal.pone.0196822)
Supplement: S2 File — (DOC) [file pone.0196822.s002.doc]

**PRETEST: DO NOT CIRCULATE!!!!!!**

__________

AFGHAN COMMUNITY NEEDS ASSESSMENT SURVEY:

A Social Psychological Survey

Hello, my name is ___________________________

I am part of a team of researchers affiliated with California State University, East Bay conducting a “needs assessment study” of Afghans in Alameda County. The results of the study will benefit the Afghan community by supporting the funding and development of programs to assist Afghans of all ages. Your participation is important for the study to accurately represent the life experiences of Afghans’ living in Alameda County.

Your participation in the study will consist of answering questions about your life in Afghanistan and the U.S., your family, cultural practices, children’s schooling, health needs, and your views on programs that might assist Afghans who are adjusting to life in the Bay Area and the United States. The survey will take about 90 minutes to complete.

Your answers will be *strictly confidential*. This means that your name will not be associated with your answers, and the interviewer and the researchers will not, under any circumstances, disclose your answers to anyone. This includes government agencies like social services or INS. Your answers will *only* be combined with those of other Afghans to find out about the community as a whole.

All of us on the research team thank you for helping us determine the most important needs of the Afghan community.

1. Date [ e.g 8/13]: ____________
2. Location: 1. Interviewee home 2. Survey center 3. Internet 4. Other______________
3. Interviewer code number:_____
4. Interviewer gender: 1. Male 2. Female
5. Interviewer age:_____
6. Interviewer ethnicity: a. Hazara b. Pushtun c. Tajik d. Turkman e. Uzbek
   other: __________
7. Language(s) in which interview was conducted (circle letter of all languages used):
   a. English b. Dari c. Pashto d. Other ________________________
8. Number of others accompanying interviewee during the interview (if any)? ______
   **If no one is accompanying the interviewee, skip next question.**
9. What is the relationship of these others to the interviewee?
    Relationship
   a. Person #1 accompanying interviewee: ____________________________
   b. Person #2 accompanying interviewee: ____________________________
   c. Person #3 accompanying interviewee: ____________________________
10. How interviewee was sampled: 1. AHPP 2. Mosque 3. Internet 4. Other: Pretest

**Interviewee Background:**

1. Gender of the Interviewee: 1. Male 2. Female
2. How old are you? _______
   **[If the interviewee does not know his or her age or is reluctant to disclose, ask for an approximation.]**
3. In which city do you reside? _______________________________________
4. What country were you born in?
   1. Afghanistan **[Skip next two questions]**
   2. U.S.
   3. Pakistan [Skip next question] 4**.** Other __________________________
5. Were you born in the Bay Area?
   1. Yes
   2. No
6. Did you ever live in Afghanistan? 1. Yes 2. No
7. Who in your immediate family made the decision (for you) to leave Afghanistan? Was it you or your spouse, your parents, one of your children, or who? **[Ask “for you” if interviewee left Afghanistan. If the interviewee did not live in Afghanistan leave off “for you.”]**
   1. Self
   2. Spouse
   3. Self and spouse
   4. Parents
   5. Grandparents
   6. Children
   7. Other:______________________________________________________
8. What year did you/your family leave Afghanistan? *Some people left Afghanistan more than once because they returned. If you/your family left more than once, please answer the year that you/your family last left Afghanistan.* **[ ask “your family” if interviewee never lived in Afghanistan]** _________
9. a. Where in Afghanistan did you/your family live before leaving? [**If interviewee answers more than one place, ask:
   b. “Where did you live the most during the five years prior to leaving Afghanistan?” and circle the answer to that question.]**
   _________________________________________________________________________
   __________________________________________________________________________________________________________________________________________________
10. Did you/your family live anywhere else after leaving Afghanistan and before arriving in the Bay Area?
    1. No [**Skip next question]**
    2. Yes
11. Please list the cities and countries you lived in order of the first one after leaving Afghanistan to the last one before arriving in the Bay Area. **[Ask how long they stayed in each city and write length of stay next to each city. e.g “New York – 3 months”]________________________________________________**_________________ ___________________________________________________________________________________________________________________________________________________________________________________________________________________________
    _________________________________________________________________________
12. What year did you/your family arrive in the United States? ___________
13. What year did you/your family arrive in the Bay Area? ____________
14. In which city or town did you live when you first arrived/were born in the Bay Area? **[List them below them in order of the first city lived in to the most recent one. If there is no code, write out the name. ]**

1. Alameda
2. Berkeley
3. Castro Valley
4. Concord
5. Dublin
6. Fremont
7. Hayward
8. Livermore
9. Milpitas
10. Newark
11. Oakland
12. Pittsburgh
13. Pleasant Hill
14. Pleasanton
15. San Jose

16. San Francisco
17. San Leandro
18. San Lorenzo
19. San Ramon
20. Tracy
21. Union City
22.Walnut Creek

23. Other:______________________________________________________

SOCIAL CONNECTIONS

1. Have you returned to Afghanistan to visit or work since 2001?
   1. Yes
   2. No
2. Do you hope to one day return *to live* in Afghanistan? [“Live” means set up a permanent residence.]
   1. No **[Skip next question]**
   2. Yes
3. In which other countries other than the U.S. do you have family members that you stay in contact with? [**Select all that apply]**

aa. Afghanistan

a. Australia

b.Canada
c. Pakistan

d. Denmark

e. Egypt

f. Finland

f. France

g. Germany
h. Netherlands

i. Iran

j. Russia

k. Sweden

l. Tajikistan

m. United Kingdom

n. Other1 (please specify):________________
o. Other2 (please specify):________________

p. Other3 (please specify):________________

1. How often have you visited Afghan family and/or Afghan friends who live in countries other than United States *in the past five years*?
   1. Not in the past five years
   2. Once in the past five years
   3. 2 to 3 times
   4. 4 to 5 times
   5. 6 to 10 times
   6. More than 10 times
2. How would you identify your ethnicity? Is it on this list or is it another ethnicity? You may choose more than one.
   a. Hazara g. ______
   b. Pushtun h. ______
   c. Tajik i. ______
   d. Turkman j. ______
   e. Uzbek k.______
   f. Other:_______________________________ l._______
   g. None
   [**For each ethnic identity named or listed above ask the following question:]**
   How important is your [ fill-in ethnicity ] identity to you? Is it not important, somewhat important, important, or very important to you? **[Write the number corresponding to the importance of identity in the space next to the ethnic identity. e.g. Hazara importance goes in g. _____ ]**
   1. Not important 2. Somewhat important 3. Important 4. Very important
3. How important is your “American” identity to you?
   1. Not important 2. Somewhat important 3. Important 4. Very important
4. How important is your “Afghan” identity to you?
   1. Not important 2. Somewhat important 3. Important 4. Very important

Next, I will ask you about your language skills in Dari, Pashto, and English.

How well do you speak Dari? Are you very fluent, intermediate, fair, a little, or not at all? How well do you read Dari? How well do you write Dari?

How well do you speak Pashto? How well do you read Pashto? How well do you write Pashto?

How well do you speak English? How well do you read English? How well do you write English?

| Language | a. Speaking | b. Reading | c. Writing |
| --- | --- | --- | --- |
| 1. Dari/Farsi | 1. Very Fluent  2. Intermediate  3. Fair  4. A little  5. Not at all | 1. Very Fluent  2. Intermediate  3. Fair  4. A little  5. Not at all | 1. Very Fluent  2. Intermediate  3. Fair  4. A little  5. Not at all |
| 1. Pashto | 1. Very Fluent  2. Intermediate  3. Fair  4. A little  5. Not at all | 1. Very Fluent  2. Intermediate  3. Fair  4. A little  5. Not at all | 1. Very Fluent  2. Intermediate  3. Fair  4. A little  5. Not at all |
| 1. English | 1. Very Fluent  2. Intermediate  3. Fair  4. A little  5. Not at all | 1. Very Fluent  2. Intermediate  3. Fair  4. A little  5. Not at all | 1. Very Fluent  2. Intermediate  3. Fair  4. A little  5. Not at all |

1. Are you currently a citizen of the U.S., a permanent resident (green card), holding a work visa, an education visa, some other visa, or something else?
   1. Citizen
   2. Permanent resident
   3. Work visa
   4. Student visa
   5. Other visa (specify: __________________________________)
   6. Other:________________________________________________

**Marriage and Marriage Relationships**

1. What is your present marital status?
   1. Married
   2. Widowed
   4. Divorced [**Skip next question]**
   5. Separated, because you and your spouse are not getting along
   6. Separated, because your spouse must live in another city or country
   7. Never been married [**Skip next 6 questions]**
   8. Other:_____________________________________
2. Have you ever divorced?
   1. Yes
   2. No [**Skip to next two questions]**
3. How many times have you divorced? ______
4. If you have divorced, who decided to end your (most recent) marriage?
   1. My former spouse did
   2. I did
   3. We both decided to end it at the same time
5. How old were you when you first married?____
6. How many times have you married?_____
7. [**Ask only if interviewee is or has been married]** Did your parents or other family members arrange your marriage(s) or did you choose your spouse(s)?
   1. My parents/family arranged my marriage
   2. My parents/family arranged my marriage, but I chose whether to go through with it.
   3. I chose my spouse, but my parents then arranged it
   4. I chose my spouse
8. Afghans living in the U.S. culture are faced with a marriage system where some parents have a much smaller role in choosing spouses than in Afghanistan. In your family, has dealing with two different marriage systems caused no conflict, a little conflict, moderate conflict, or a great deal of conflict?
   1. None
   2. A little conflict
   3. Moderate conflict
   4. A great deal of conflict
9. **[Ask if presently married]** Overall, would you say that your marriage is very happy, pretty happy or not too happy?
   1. Very happy
   2. Pretty happy
   3. Not too happy
10. Do you think too many Afghans in the U.S. are divorcing these days? If yes, is this problem somewhat serious or very serious?
    1. No **[Skip next two questions**]
    2. Yes, somewhat serious
    3. Yes, very serious
11. Do you think that an important cause of greater divorce for Afghans is that women have more rights in the U.S.?
    1. Yes
    2. No
    3. Not sure
12. Do you think that more opportunities for marriage counseling that fits Afghan culture would help Afghans maintain more stable marriages? If yes, would it help a great deal or just somewhat?
    1. Yes, it would help a great deal.
    2. Yes, it would help somewhat
    3. No
    4. Not sure
13. a. If a married couple is having serious conflicts, who would be the best person or organization to get help from? _________________________________________________________________
14. Which type of marriage would you prefer, the marriage described in statement one or statement two?
    1. A marriage where the man has the main responsibility for providing the household income and the woman has the main responsibility for taking care of the home and family.
    2. A marriage where the man and woman equally share responsibility for providing the household income and taking care of the home and family.
15. **[ Ask if presently married and living together] Comparing yourself to your spouse, do you think that you do your fair share of work around the house? Or do you think that you should be doing more of the housework or less of it?
    1. I do my fair share
    2. I should be doing more
    3. I should be doing less
    97. Don't know
    98. Refused
    99. NA**

**HOUSEHOLD CENSUS**

1. Next, we will ask you about your living family members and which family members are residing in your household. **[1 = Yes; 0 = No] [Recommendation: Ask “how many living” for all items first, then ask, “Which family members are presently living with you in your household?”**

| **How many living _______** | **do you have?** | **How many live with you?** |
| --- | --- | --- |
| grandparents | a. | m. |
| Is your father living? | b. | n. |
| Is your mother living? | c. | o. |
| stepmothers | d. | p. |
| uncles | e. | q. |
| aunts | f. | r. |
| brothers | g. | s. |
| sisters | h. | t. |
| sons | i. | u. |
| daughters | j. | v. |
| grandsons | k. | w. |
| granddaughters | l. | x. |

1. Other than the family members you have mentioned, who else lives in your household? **[list additional household members by their relations to interviewee (e.g. friend, mother-in-law)]___________________________________________________________**
   __________________________________________________________________________________________________________________________________________________
2. Apart from those who live in your home, how many of your relatives live in the Bay Area? ______ **[If not sure, please estimate]**
3. Apart from those who live in you home, how many relatives do you see once a month or more often than once a month?____

Please answer the following four questions about your children and/or grandchildren living in the U.S., *whichever* *has not married yet*. If you do not have children, please answer the question in terms of if you had children, what you would want for them.

1. Who are you answering these questions about?
   1. My grandchildren
   2. My children
   3. My future children
2. How important to you is it (was it/will it be) for your [ fill-in answer] to marry another Afghan?
   1. Not important
   2. Somewhat important
   3. Very important
3. Do you think that your [fill-in answer ] should be allowed to choose their own spouses when they are old enough to marry?
   1. No. It is best for parents to make those decisions
   2. Somewhat. Children and parents should work together to find a good spouse
   3. Yes, children should be trusted to make their own choices
4. Do you think your [fill-in answer ] should be able to go out on dates with someone of the opposite sex once they have completed high school?
   1. Yes
   2. No
   3. Not sure
5. How important to you is it for [fill-in answer ] to marry someone of your same religion?
   1. Not important
   2. Somewhat important
   3. Very important

**RELIGION**

1. What religion, if any, are you?
   1. Islam
   2. Christianity
   3. Hinduism
   4. Judaism
   5. Sikhism
   6. Buddhism
   7. Other:_______________
   8. None
   (**If not Muslim, skip next question)**
2. If you are a Muslim, can you be more specific?
   1. Hanafi Sunni
   2. Jafari (Twelver) Shia
   3. Ismali (Sevener) Shia
   4. Other: _____________________________________________
3. Are you presently a member of a mosque?
   1. Yes
   2. No
4. How frequently do you attend religious services or prayers (Mosque)?
   1. More than once a week
   2. Once a week
   3. 1-3 times per month
   4. Less than once a month
   5. Never
5. How important is religion (Islam) in your daily life?
   1. Not important
   2. Somewhat important
   3. Very important
6. Do you think it is important for all peoples to respect and get along with those whose religion differs from their own?
   1. Yes, strongly agree
   2. Yes, somewhat agree
   3. No, somewhat disagree
   4. No, strongly disagree
7. How much do you practice Ramadan?
   1. Rigorously, I strictly follow the rule for fasting
   2. Somewhat, I follow the rules as much as I can
   3. Socially, follow the rules in public, but not in private
   4. Very little or not at all
   5. I am not fasting because of age or health problems

**Exposure to War and Forced Migration-Related Traumas and Stresses [Much of this section is for respondents who migrated from Afghanistan. If the interviewee was young (9 years or younger) when he or she left ask, “Do you remember about the violence and danger in Afghanistan?” If interviewee does not remember anything about it, then ask # 67, but Skip 68-72]**

The following questions are about your experiences with the violent wars in Afghanistan. If any of the questions are making you uncomfortable, please tell me and we will move on to different questions.

1. Before leaving Afghanistan did you and your family have enough income to meet your needs?
   1. Not enough income for family needs
   2. Barely enough income for family needs
   3. Adequate income but no extras
   4. Adequate income and some extras
   5. More than adequate income
2. Was your safety or your life ever threatened during one of the coups or wars in Afghanistan or when you escaped from Afghanistan?
   1. Yes
   2. No
3. Were you ever imprisoned, captured, or held hostage by the military, police, mujahadeen, Taliban, or other members of warring factions?
   1. Yes
   2. No
4. If yes, were you tortured or brutalized while imprisoned?
   1. Yes
   2. No
5. **[If yes to any of the three previous questions. If no to all three, skip the next four questions]** Did you sustain serious physical injuries or impairments as a result of the wars and violence in Afghanistan?
   1. Yes
   2. No
6. If yes, what physical injuries did you sustain?____________________________________
   __________________________________________________________________________________________________________________________________________________
7. Did any of your close family members or close friends have their lives threatened during one of the coups or wars in Afghanistan?
   1. Yes
   2. No
8. Were any of your close family members or close friends severely injured or harmed during one of the coups, wars or battles in Afghanistan?
   1. Yes
   2. No
9. Were any of your close family members or close friends killed or missing during one of the coups, wars or battles in Afghanistan?
   1. Yes
   2. No
10. If yes, how many close family members or close friends were killed or missing?________
11. Did you witness any family members or close friends being killed or seriously injured? **[Skip if interviewee did not live in Afghanistan]**
    1. Yes
    2. No
12. You have described [summarize the traumatic events the interviewee has experienced]. Did these events cause you serious emotional distress, fears or grief?
    1. Yes
    2. No
13. If yes, please describe your emotional and mental suffering at the time? (How did the traumatic events affect you emotionally and mentally at the time**?)_[Probe on intensity and frequency of suffering]________________________________**__________________
    ____________________________________________________________________________________________________________________________________________________________________________________________________________________________________________________________________________________________________
14. At the time, did the mental and emotional suffering make it difficult to function in your daily life?
    1. Yes, it great impaired my functioning
    2. Yes, it somewhat impaired my functioning
    3. No
15. If yes, how did you cope with the [summarize emotional distress/suffering]? What did you do to recover from the distress you experienced?__________________________________
    ____________________________________________________________________________________________________________________________________________________________________________________________________________________________________________________________________________________________________
16. At the time did you receive assistance to help you recover from [name of emotional distress/suffering]?
    1. Yes
    2. No
17. Who provided this assistance? **[Be specific, if possible]**______________________________
    __________________________________________________________________________________________________________________________________________________
18. Do you still suffer today as a result of the events you experience in Afghanistan?
    1. Yes, a great deal
    2. Yes, somewhat
    3. No
19. How did you leave or escape from Afghanistan? **[select all that apply]**
    a. Car/Truck
    b. On foot
    c. Donkeys and horses
    d. Airplane
    e. Bus
    e. Other (specify:_________________________________________________________)
20. During any of the wars or coups in Afghanistan were you “internally displaced,” where you had to leave your home and move to another part of Afghanistan to secure your safety?
    1. Yes
    2. No **[Skip next question]**
21. For how long were you internally displaced?

1. 1-3 months
2. 4-6 months
3. 7-9 months
4. 10-12 months
5. 13-18 months
6. 19-24 months
7. 25 months to 3 years
8. 4-5 years
9. 6-7 years
10. 8-9 years
11. 10 or more years

1. When you left Afghanistan were you displaced in a country surrounding Afghanistan?
   1. Yes
   2. No **[Skip next question]**
2. For how long were you displaced in a country surrounding Afghanistan?

1. 1-3 months
2. 4-6 months
3. 7-9 months
4. 10-12 months
5. 13-18 months
6. 19-24 months
7. 25 months to 3 years
8. 4-5 years
9. 6-7 years
10. 8-9 years
11. 10 or more years

1. After leaving Afghanistan, did you stay in a refugee camp?
   1. Yes
   2. No **[Skip this skip next question]**
2. For how long did you stay in the refugee camp(s)? **[If more than one, total time in all camps]**

1. 1-3 months
2. 4-6 months
3. 7-9 months
4. 10-12 months
5. 13-18 months
6. 19-24 months
7. 25 months to 3 years
8. 4-5 years
9. 6-7 years
10. 8-9 years
11. 10 or more years

**ASK 91-95 ONLY** **IF INTERVIEWEE ANSWERED YES TO INTERNAL DISPLACEMENT OR SURROUNDING DISPLACEMENT OR REFUGEE CAMP**

1. Did you have adequate food and water while displaced or in the refugee camp?
   1. Yes, most or all of the time
   2. No, sometimes there was not enough food or water
   3. No, very often there was not enough food or water
2. Did you have adequate medical care while displaced or in the refugee camp?
   1. Yes, most or all of the time
   2. No, sometimes illnesses and injuries received poor treatment
   3. No, very often illnesses and injuries received poor treatment
3. Was your life ever threatened or were you seriously harmed while displaced or in the refugee camp?
   1. Yes
   2. No
4. Did you suffer severe emotional or mental distress while displaced or in a refugee camp?
   1. Yes
   2. No
5. Were any of your family members or close friends seriously harmed or did any have a serious illness, while displaced or in the refugee camp?
   1. Yes
   2. No

**End of skipping refugee camp questions - everyone answers the following**

1. Do you suffer now because other family members are not with you, or could not leave the country?
   1. Yes, very much
   2. Yes, somewhat
   2. No
2. Would you like to reunite your family? If yes, is this somewhat or very important to you?
   1. Yes, very important
   2. Yes, somewhat important
   3. No

**Now we are going to ask you about resettling in the United States and the Bay Area**

1. How many close family members or close friends were already living in the U.S. when you/your family arrived? ______ **[If not sure, please estimate]**
2. You said you/your family arrived in the Bay Area in [pipe in q23]. Which family members arrived in the Bay Area at this time?
   a. Self a. Grandparents
   b. Mother
   c. Father
   d. Spouse
   e. Siblings
   f. Children
   g. Grandchildren
   h. Other:________________________________________________________________
3. Overall, how difficult has it been *for you/* *your* *family* to resettle and get adjusted in the Bay Area? Would you say it has been….
   1. Not very difficult
   2. Somewhat difficult
   3. Very difficult
4. **[If somewhat or very difficult]** What were/are the biggest difficulties you or your family has had while resettling in the Bay Area? ___________________________________________________________________________________________________________________________________________________________________________________________________________________________
5. How much assistance, if any, did you/your family receive when you first arrived in the Bay Area from family and friends who were already here?
   1. None
   2. A little
   3. Moderate
   4. A lot
6. How much assistance, if any, did *you/your family* receive from Afghan organizations or other non-governmental organizations when you first arrived in the Bay Area?
   1. None
   2. A little
   3. Moderate
   4. A lot
7. Have you or your family received assistance from any Afghan organizations or other non-governmental organizations at any time since you first arrived?
   1. Yes
   2. No
8. If yes, which Afghan organizations did you receive assistance from and what did they help you with (e.g. immigration papers, housing, work, health)?
   ________________________________________________________________________________________________________________________________________________________________________________________________________________________________________________________________________________________________
9. Overall, how helpful do you feel that local Afghan organizations have been for Afghans living in the Bay Area?
   1. Not helpful
   2. Somewhat helpful
   3. Very helpful
10. Did you or family members living in your household receive assistance from federal, state, or local governments when you arrived in the Bay Area?
    1. Yes
    2. No
11. If yes, what social services did you/your family receive? [Select all that apply)
    a. Social insurance or social security
    b. Medical Care (e.g. Medi-cal, Medicaid, Medicare)
    c. Disability benefits (e.g. SSI)
    d. Food stamps or food vouchers
    e. Welfare or general assistance or cash assistance (CAPI)
    f. Housing assistance or subsidized housing
    g. Childcare assistance or subsidized childcare
    h. Unemployment insurance
    i. Educational grants
    j. Psychological counseling
    k. Other (specify:_____________________________________________________)
12. Does anyone in your household *presently* receive assistance from federal, state, or local governments?
    1. Yes
    2. No
13. What social services do household members presently receive? (circle the letter of all that apply)
    a. Social insurance or social security
    b. Medical Care (e.g. Medi-cal, Medicaid, Medicare)
    c. Disability benefits (e.g. SSI)
    d. Food stamps or food vouchers
    e. Welfare or general assistance
    f. Housing assistance or subsidized housing
    g. Childcare assistance or subsidized childcare
    h. Unemployment insurance
    i. Educational grants
    j. Psychological counseling
    k. Other (specify:_____________________________________________________)
14. Which, if any, social services or supports do you or your family most need now, that you are not presently receiving? _________________________________________________
    _______________________________________________________________________________________________________________________________________________
15. In the past year, did you and family members in your household send remittances (money) to relatives or friends living abroad?
    1. Yes
    2. No
16. Have the remittances you send or other charitable donations made to needy Afghans caused any economic hardship for you or your family over the past year?
    1. Yes
    2. No
17. How often do you or household members communicate with relatives or friends inside of living abroad?
    1. Daily
    2. Weekly
    3. Monthly
    4. Yearly
    5. Less than once per year
    6. Not at all
18. Not counting those in your household, how many Afghans live in your neighborhood?
    1. No other Afghans live in our neighborhood
    2. A few Afghan live in our neighborhood, but not very many
    3. Quite a few Afghans live in our neighborhood
19. How often do you interact or visit other Afghans in your neighborhood?
    1. Not at all
    2. Occasionally
    3. Sometimes
    4. Quite often
20. How many close friends do you have in the Bay Area that you can rely on if you need help?_____
21. How many of these close friends in the Bay Area are *not* Afghans?_____
22. Would you or a family member be interested in living in senior housing or a senior community that is designed to serve Afghans?
    1. Yes
    2. Possibly
    3. No

**EDUCATION**

1. **[Ask only those who lived in Afghanistan after age 5]** Did you ever attend school in Afghanistan?
   1. No **[Skip the next question]**
   2. Yes
2. What standard or level did you reach in Afghanistan? 1. Some grade school
   2. Finished grade school
   3. Some junior high or middle school
   4. Finished middle school
   5. Some high school
   6. High school degree
   7. Trade School
   8. Some College
   9. College Degree
   10. M.D.
   11. Other Advanced professional degree
   12. Ph.D.
   13. Other:________________________
3. Have you ever attended school in United States?
   1. Yes
   2. No

1. Which level of education did you complete in the U.S.? **[Select all the apply]**
   a. Primary or grade school
   b. Middle or Junior High School
   c. High School Degree
   d. GED
   e. Degree from a trade school (specify:_________________________________________)
   f. Technical degree from a community college
   g. 2 year college degree (AA )
   h. Bachelors (BA or BS) (4 year college degree)
   i. Masters (MA or MS)
   j. Professional degree (eg. J.D. or M.D.)
   k. Ph.D.
   l. ESL Certificate
   m. Other:______________________________________________
2. **[If interviewee graduated from trade school, community college, or university in the U.S.]** At what school did you earn you *highest* degree?
   1. Chabot
   2. Ohlone
   3. CSU East Bay
   4. San Jose State
   5. San Francisco State
   6. UCSF
   7. UC Berkeley
   8. UC Davis
   9. Other: __________________________________________________________
3. [**Skip if it is clear that the answer is no]** Did you earn your highest degree or level of education in a country other than Afghanistan or the U.S.?
   1. Yes
   2. No
4. If yes, in what country was this and what standard or level did you reach in this country?
   ________________________________________________________________________
5. What is the highest level of education your mother reached?________________________
6. What is the highest level of education you father reached?___________________________
7. Earlier you said you have __ boys and __ girls. Have any of your children attended schools in the U.S.?
   1. Yes
   2. No **[Skip next question]**
8. What are your children’s ages and highest level of education attained*,* thus far?
    **Education Age**
   Oldest son a.______ e.______
   Youngest son b.______ f.______
   Oldest daugther c.______ h.______
   Youngest daughter d.______ i.______

**0. None**

**1. Some primary school**
**2. Completed primary school**
**3. Some Middle/Junior High**
**4. Finish Middle/Junior High**
**5. Some High School**
**6. High School Degree**
**7. GED**
**8. Finish trade school
9. 2-year technical degree
10. Some college (no degree)**
**11. 2 year college degree (AA )**
**12**. **Bachelors (BA or BS)**
**13. Masters (MA or MS)
13. Professional degree
14. Ph.D.**

**Employment/Occupations**

1. Which one of these best describes your present employment situation?
   1. Employed, working 45 or more hours per week [**Skip next question]**
   2. Employed, working 35-44 hours per week [**Skip next question]**
   3. Employed, working 25-34 hours per week [**Skip next question]**
   4. Employed, working less than 25 hours per week [**Skip next question]**
   5. Unemployed and looking for work
   6. Unemployed and not looking for work
   7. Keeping house (homework)
   8. Retired
   9. Disabled
   10. Other (Specify:____________________________________________________)
2. How many years has it been since you were last employed? **[Skip next five questions after answering this one**]
   1. Less than one year
   2. Between one and three years ago
   3. Between three and five years ago
   5. Between five and ten years ago
   6. More than ten years ago
   7. Never employed
3. What is your main occupation, what do you do (main duties), and what kind of place do you work for? (Please describe clearly)
   a. Occupation and main duties:____________________________________________
   _____________________________________________________________________
   b. Kind of employer (what the company does or makes? OR what government agency or other organization do you work for?)
   [*Examples*: used auto sales, computer networking hardware and software corporation, grocery store chain, California department of motor vehicles, public high school. *Note*: if you are self-employed, write “self-employed.”]
   __________________________________________________________________________________________________________________________________________________
4. Which of the two statements best describes your *main* occupation?
   1. It does not allow me to use my training, experience and abilities. I am capable of more challenging work and greater responsibility.
   2. It allows me to use my training, experience, and abilities. I find my work challenging.
5. Thinking of your main occupation, which best describes your co-workers?
   1. Most are Afghan
   2. Most are non-Afghan, foreign-born
   3. Most are non-Afghan, American-born
6. **[Ask only for those who were age 18 or older when they left Afghanistan]** Did you have a paying job in Afghanistan *before* migrating to the U.S.?
   1. Yes
   2. No **[Skip next two questions]**
7. What was your main occupation in Afghanistan *before* migrating to the U.S. (Please describe clearly) and what kind of place did you work?
   a. Occupation and main duties:____________________________________________
   _____________________________________________________________________
   b. Kind of employer (what the company did or made? OR what government agency or other organization did you work for?)____________________________________
   _____________________________________________________________________
8. [**Ask only if presently married]** Which one of these best describes your spouse’s present employment situation?
   1. Employed, working 45 or more hours per week [**Skip next question]**
   2. Employed, working 35-44 hours per week [**Skip next question]**
   3. Employed, working 25-34 hours per week [**Skip next question]**
   4. Employed, working less than 25 hours per week [**Skip next question]**
   5. Unemployed and looking for work
   6. Unemployed and not looking for work
   7. Retired
   8. Keeping house (homework)
   9. Disabled
   10. Other: Specify: ______________________________________________________
9. What is your spouse’s main occupation, what does she or he do (main duties), and what kind of place does she or he work for? (Please describe clearly)
   a. Occupation and main duties:____________________________________________
   _____________________________________________________________________
   b. Kind of employer (what the company does or makes? OR what government agency or other organization does she or he work for?)
   *Examples*: auto sales, computer networking hardware and software corporation, chain grocery store, California Department of Motor Vehicles, public high school *Note*: if you spouse is self-employed, write “self-employed”
   __________________________________________________________________________________________________________________________________________
10. Did your spouse have a paying job in Afghanistan *before* migrating to the U.S.?
    1. Yes
    2. No **[Skip to ___ ]**
11. What was your spouse’s main occupation in Afghanistan *before* migrating to the U.S. (Please describe clearly) and what kind of place did you work?
    a. Occupation and main duties:____________________________________________
    _____________________________________________________________________
    b. Kind of employer (what the company did or made? OR what government agency or other organization did you work for?)____________________________________
    _____________________________________________________________________
12. Would you (or a close family member) like to participate in a network of Afghan day laborers that would help you get one to three days jobs doing manual labor such as yard work, landscaping, and construction?
    1. Yes
    2. No
    3. Not sure
    97. Don’t know
    98. Refused
13. Would you (or a close family member) like to participate in a two to three week training program in Dari or English that would teach you how to search for jobs, how to fill out applications, and how to present yourself during job interviews?
    1. Yes
    2. No
    3. Not sure
    97. Don’t know
    98. Refused
14. What was your total household income from all sources for the last 12 months?

1. $0 – 4,999
2. $5,000 – 9,999
3. $10,000 – 14,999
4. $15,000 – 19,999
5. $20,000 – 29,999
6. $30,000 – 39,999
7. $40,000 – 49,999
8. $50,000 – 59,999
9. $60,000 – 69,999
10. $70,000 – 79,999
11. $80,000 – 89,999
12. $90,000 – 99,999
13. $100,000 – 249,999
14. $250,000 or more

**Prejudice and Discrimination**

1. After the tragic events of September 11, 2001, were you more concerned that non-Afghans might harm you or someone in your family?
   1. Yes, much more concerned
   2. Yes, somewhat more concerned
   3. No, not more concerned
2. After September 11, 2001, when going through security checks at an airport, were you ever pulled out of the line to ask you extra questions or check you and your luggage more thoroughly?
   1. No, I have *not* done any air travel since 9/11/2001
   2. No, I have flown, but have not been checked
   3. Yes, one time
   4. Yes, two times
   5. Yes, three times
   6. Yes, four times
   7. Yes, five or more times
3. Discrimination is when someone harms another person simply because of the group he or she is part of. For example, if an employer does not hire an Afghan or a Muslim because the applicant is Afghan or Muslim, this is discrimination. Since living in the Bay Area, were you ever discriminated against because you are Afghan, when applying for a job or a promotion in your present job, seeking housing, in school, or some other way?
   1. Yes, definitely
   2. Yes, I am pretty sure
   3. No, I don’t think so **[Skip next question]**
   4. No, definitely not **[Skip next question]**
4. If yes, how or where have you been discriminated against? (Please circle the letter of each area where you have faced discrimination.) **[Show card]**
   a. When applying for a job
   b. Seeking a job promotion or being unfairly evaluated on your job
   c. When renting a home/apartment
   d. When buying a home/condo
   e. Applying to a school or university or other educational program
   f. When attending school by a teacher or counselor
   g. By the police
   h. By the courts
   i. In a store, restaurant, or other public place
   j. Other way #1 (specify: _____________________________________________)
   k. Other way #2 (specify:_____________________________________________)
5. Do you think that equally qualified Afghans have equal chances of getting jobs compared to other Americans?
   1. Yes
   2. No
   3. Not sure

**Acculturation**

1. **[Ask if parents are living]** What language do you use when speaking with your *parents*?
   1. Only Dari or Pashto
   2. Only English
   4. More Dari or Pashto, but some English
   5. More English, but some Dari or Pashto
   6. Dari/Pashto and English equally
   7. Other language (specify): _____________________________________________
   8. I do not have any parents
2. [**Ask if has children]** What language do you use when speaking with your *children*?
   1. Only Dari or Pashto
   2. Only English
   4. More Dari or Pashto, but some English
   5. More English, but some Dari or Pashto
   6. Dari/Pashto and English equally
   7. Other language (specify): _____________________________________________
   8. I do not have any children
3. When watching television, how often do you watch Afghan programs?
   1. Not at all
   2. Occasionally Less than half of the time
   3. More than half, but not all of the time
   4. All of the time
   5. I don’t watch television
4. What is the ethnic background of the poets, writers, musicians, singers, and actors that you most admire?
   1. All are Afghan
   2. Most are Afghan
   3. About half are Afghan and half are non-Afghan
   4. Most are non-Afghan
   5. All are non-Afghan
5. Do you have access to a library that has a substantial number of books, newspapers, magazines, and other materials in Dari or Pashto?
   1. Yes
   2. No
   3. Not sure
   97. Don’t know
   98. Refused
6. If it is/was available do/would you use a library that has books, newspapers, magazines, and other materials in Dari or Pashto?
   1. No
   2. Yes, rarely
   3. Yes, occasionally
   4. Yes, pretty often
   5. Yes, very often
7. In the past 12 months have you done any volunteer work for an Afghan organization?
   1. No
   2. Yes
8. In the past 12 months have you done any volunteer work for any other civic or community organization such as Rotary, a parent-teacher association, a religious organization, or a sports organization?
   1. No
   2. Yes
9. Have you attended Afghan cultural events such as movies, concerts, poetry readings, or lectures in the past year? If yes, how many times?
   1. No
   2. Yes, one time
   3. Yes, two times
   4. Yes, three times
   5. Yes, four or more times
10. Would you like there to be more Afghan cultural events to attend in the East Bay? If yes, is this very important or somewhat important to you?
    1. Yes, very important
    2. Yes, somewhat important
    3. No
11. Did you vote in the 2006 state and federal elections?
    1. Yes
    2. No

**Parenting**

**[Now we will ask you some questions about raising Afghan children in the East Bay.]**

**[Ask this first set of questions if the interviewee is *presently* raising a child between age 5 and 18.]**

1. Have you ever contacted one of your child’s (children’s) teachers to discuss how your child is progressing in school?
   1. Yes, once or twice
   2. Yes, several times
   3. Yes, frequently
   2. No
2. How often do you or your spouse help your children with their homework?
   1. Not at all
   2. Rarely
   3. Occasionally
   4. Regularly
3. How worried are you about the negative influences on your child(ren) of other students in their schools?
   1. Not at all
   2. A little
   3. Somewhat
   4. Very
4. Do you think it is good for your children to have friends who are not Afghan?
   1. Yes
   2. No
   3. Not sure

Now we will ask some questions about programs for Afghan children.

**[These questions are for all interviewees.]**

1. Imagine a child under the age of 3 years old whose parents both have full-time jobs during the day. Which of the following child care arrangements do you think would be the *two* best arrangements?
   a. A public day care center
   b. A private day care center
   c. A day care center located at a Mosque or other religious institution
   d. An in-home day care provider
   e. A babysitter
   f. A neighbor
   g. A family member
   h. Other:___________________________________________________
   i. None, a child under age three should have a parent caring for him or her.
2. **[Ask is interviewee has child age 16 or under]** Do you presently need quality day care or after school care, but are unable to find such care that you can afford?
   1. Yes
   2. No
   97. Don’t know
   98. Refused
   99. NA

--------------------------------------

1. Would you like there to be workshops or seminars available for Afghan youth to learn about the dangers of using and selling drugs?
   1. No, this is unnecessary
   2. Not sure
   3. Yes, this is somewhat important
   4. Yes, this is very important
   97. Don’t know
   98. Refused
2. If there were workshops or seminars for Afghan youth on the dangers of using and selling drugs, would you like them to be taught from the perspective of Sharia law?
   1. No
   2. Not sure
   3. Yes, it should include this perspective
   4. Yes, it should be taught primarily from this perspective
   97. Don’t know
   98. Refused
3. Would you like there to be programs on Afghan television that focus on the challenges of raising children in the United States? If yes, are you very interested or somewhat interested in this?
   1. Yes, very interested
   2. Yes, somewhat
   3. No
4. Would you like there to be more classes and programs available for Afghan youth to improve their reading, writing, and verbal skills in the Dari and Pashto languages?
   1. Yes
   2. No
   3. Not sure
   97. Don’t know
   98. Refused

**[If the interviewee has children]** Speaking of your *child closet to age 10*, how well does he/she speak, read and write Dari, Pashto, and English? **[If the interviewees** **children are all adults, ask about their youngest child.]**

| Language | a. Speaking | b. Reading | c. Writing |
| --- | --- | --- | --- |
| 1. Dari/Farsi | 1. Very Fluent  2. Intermediate  3. Fair  4. A little  5. Not at all | 1. Very Fluent  2. Intermediate  3. Fair  4. A little  5. Not at all | 1. Very Fluent  2. Intermediate  3. Fair  4. A little  5. Not at all |
| 1. Pashto | 1. Very Fluent  2. Intermediate  3. Fair  4. A little  5. Not at all | 1. Very Fluent  2. Intermediate  3. Fair  4. A little  5. Not at all | 1. Very Fluent  2. Intermediate  3. Fair  4. A little  5. Not at all |
| 1. English | 1. Very Fluent  2. Intermediate  3. Fair  4. A little  5. Not at all | 1. Very Fluent  2. Intermediate  3. Fair  4. A little  5. Not at all | 1. Very Fluent  2. Intermediate  3. Fair  4. A little  5. Not at all |

1. How often do you talk to your children about Afghanistan?
   1. Never
   2. Occasionally
   3. Fairly often
   4. Very often

**[The next three questions are for any interviewees who have raised children in the U.S.**

1. Do you worry that your children will fail to maintain their Afghan culture and identity? If yes, do you worry somewhat or do you worry a lot about this?
   1. Yes, I worry about this a lot
   2. Yes, I worry about this somewhat
   3. No, this was/is not a problem
2. **[Ask if interviewee has any children age 10 or older that attend(ed) school in the U.S.]** Do you or did you ever have conflict with any of your children you are/were raising in the U.S. about becoming too Americanized or losing their Afghan culture?
   1. Yes, often
   2. Yes, sometimes
   3. Yes, rarely
   4. No, not at all
   5. N/A, no sons
3. **[Ask if interviewee arrived in the U.S. before the age of 18]** Do you or did you ever have conflict with your parents who felt you were becoming too Americanized or losing your Afghan culture?
   1. Yes, often
   2. Yes, sometimes
   3. Yes, rarely
   4. No, not at all
4. If yes, what were those conflicts most often about?________________________________
   _________________________________________________________________________
   _________________________________________________________________________

**[These following questions should be asked of all adult Afghans. ]**

1. Which aspects of American society, if any, do you think are most important for Afghan children to adopt or take advantage of? **[Interviewee may mention as many as two aspects, but if they mention more than two, ask them to choose the two most important]** _________________________________________________________________________
   __________________________________________________________________________________________________________________________________________________
2. Which aspects of American culture, if any, are **most important** for Afghan children to avoid? **[Interviewee may mention as many as two aspects, but if they mention more than two, ask them to choose the two most important]_________________________________**
   _________________________________________________________________________________________________________________________________________________________________________________________________________________________________
3. In your opinion, should Afghan parents allow their high school age children to go on group dates with members of both sexes to movies or cultural events?
   1. Yes
   2. No
   3. Not sure

**MEDICAL**

1. Where do you **usually** go when you are sick or need advice about your health? - a clinic, doctor's office, emergency room, or some other place? **[Select as many as two]**
   1. Doctor's office or
   2. HMO
   3. Health clinic
   4.Hospital emergency room
   5.Hospital outpatient department
   6.Traditional or alternative healer: a. What type of healer? _________________________ **7**. Some other place: ______________________________
   8**.** Doesn't go to one place most often
   98. Don’t Know
   99. Refused
2. Do you have any kind of health care coverage, including health insurance, group plans such as HMOs, or government plans such as Medicare or Medi-cal?
   1. Yes
   2. No **[Skip next two questions]**
3. What kind of health insurance or health care coverage do you have? **(Please circle all that apply)**
   a. Group health insurance plan from employer
   b. Private health insurance
   c. Medi-Care
   d. Medi-Cal
   e. Supplemental plans (prescription, long term, etc.)
   f. Other: __________________________________________________________________
   98. Don't know
4. Do you have insurance that covers dental care?
   1. Yes
   2. No
5. During the past 12 months, was there any time when you did not have any health insurance coverage?
   1. Yes
   2. No
   99. Don't know
6. Was there a time during the past 12 months when you needed to see a doctor, but could not because of the cost?
   1. Yes
   2. No
   99. Don't know
7. About how long has it been since you last visited a doctor for a routine checkup?
   1. Within the past year (*Anytime less than 12 months ago)*2. Within the past 2 years (*1 year but less than 2 years ago*)
   3. Within the past 5 years (*2 years but less than 5 years ago*)
   4. 5 or more years ago
8. About how long has it been since you last visited a dentist for a routine checkup?
   1. Within the past year (*Anytime less than 12 months ago)*2. Within the past 2 years (*1 year but less than 2 years ago*)
   3. Within the past 5 years (*2 years but less than 5 years ago*)
   4. 5 or more years ago
9. How would you rate your overall physical health?
   1. Excellent
   2. Very good
   3. Good
   4. Fair
   5. Poor (not good)
   99. Don't know

Has a doctor or a health care provider or mental health professional ever diagnosed you with any of the following conditions? **[ Select all that have been diagnosed]**

1. a. Heart Disease
   b. Diabetes
   c. Epilepsy
   d. Hepatitis
   e. STD
   f. HIV
   g. Hypertension/High blood pressure
   h. Kidney Disease
   i. Liver Disease
   j. Lung Disease
   k. Stroke
   l. Tuberculosis
   m. Thyroid Disease
   n. High Cholesterol
   o. Stomach or digestive problems like ulcers
   p.Bipolar
   q. Depression
   r. Anxiety disorder
   s. Post Traumatic Stress
   t. Schizophrenia
   u. Cancer
2. If cancer is selected, what type of cancer?________________________________________

Has a doctor or a health care provider or a mental health professional ever diagnosed you with any other serious medical or mental health conditions or diseases?

1. (Specify #1)_____________________________________________________________
2. (Specify #2)_____________________________________________________________
3. (Specify #3)_____________________________________________________________
4. Do you have chronic pains, like headaches and chest pain, that doctors have not been able to diagnose?
   1. Yes
   2. No
5. How many times in the past two weeks have you done a physical activity like walking, bicycling, or playing soccer that increases your breathing or heart rate? ____________
6. How many minutes do you usually do this activity? _________
7. Do you participate in any organized exercise programs or are you a member of a health club or a sports club?
   1. Yes **[Skip the next question]**
   2. No
8. Would you like to participate in an exercise program if one was available that fit your needs and that you can afford?
   1. Yes
   2. No
9. Do you avoid or limit eating foods like that are high in trans-fat and saturated fat?
   1. Yes, greatly limit
   2. Yes, somewhat limit
   3. No,
10. Do you avoid or limit eating foods high in salt?
    1. Yes, greatly limit
    2. Yes, somewhat limit
    3. No
11. Do you avoid or limit eating foods high in sugar and corn syrup?
    1. Yes, greatly limit
    2. Yes, somewhat limit
    3. No
12. Do you usually eat *whole grain* cereals, breads, and rice?
    1. Yes, almost always
    2. Yes, sometimes
    2. No
13. How often do you eat fast food like McDonald’s, Pizza Hut or Taco Bell?

1. Never of rarely
2. Occasionally: less than once a week
3. One or two times a week
4. Three or four times a week
5. Five to seven times a week
6. More than seven times a week

1. Would like the opportunity to learn more about nutrition and a healthy diet?
   1. Yes, definitely
   2. Yes, somewhat
   3. No
2. How much did you smoke cigarettes in the last four weeks?
   1. None
   2. Less than a pack each week
   3. 1-2 packs each week
   4. 3-5 packs each week
   5. About one pack each day
   6. More than one pack each day

The next questions are about how language differences influence how well you communicate with your doctor. **These first questions are about your doctor visits in the last two years.**

1. How often do the doctors you visit speak a language that you do not understand fluently? [**Show card ___** ]
   1. Never **[Skip questions on translation, to #219]**
   2. Occasionally (less than 1 in 4 doctor visits)
   3. Sometimes ( ¼ to almost ½ of my doctor visits)
   4. Often (½ to almost ¾ of my doctor visits)
   5. Very often (¾ to almost all of my doctor visits)
   6. Always
   98. DK
2. During your doctor visits *in the last two years*, have you ever had a translator present to help you understand what your doctor is saying or help you explain to the doctor what your problem is?
   1. Yes
   2. No [**Skip next three questions]**
3. If yes, who translates for you at your doctor visits **[Select all that apply]**?
   a. One of my children
   b. My spouse
   c. Other relative
   d. Friend
   e. A professional translator
   f. Other (specify) _______________________________________
4. **[If respondent answered more than one]** Which one that you selected helps you the most?
   1. One of my children [**Skip next question]**
   2. My spouse [**Skip next question]**
   3. Other relative [**Skip next question]**
   4. Friend [**Skip next question]**
   5. A professional translator
   6. Other (specify) _______________________________________ [**Skip next question]**
5. [**If respondent answered other than professional translator**] Do you think a professional translator would help you understand you doctor(s) better than [fill in answer to previous question]?
   1. Yes
   2. No
   3. The same
6. Have there been any times *in the last two years* when you needed a translator for a doctor visit, but one was not available?
   1. Yes
   2. No **[Skip next question]**
7. If yes, how often has this happened? (needed a translator, but one was not available)
   1. Rarely (once or twice)
   2. Occasionally (less than 1 in 4 doctor visits)
   3. Sometimes ( ¼ to almost ½ of my doctor visits)
   4. Often (½ to almost ¾ of my doctor visits)
   5. Very often (¾ to all of my doctor visits)
8. Have you ever requested a professional translator from your health provider or your doctor’s office when you are going in for a doctor visit?
   1. Yes
   2. No [**Skip next question]**
9. How often is a translator provided when you ask for one?
   1. Never **[Skip next question]**
   2. Rarely
   3. Sometimes
   4. Usually
   5. Always

**[End skip for translation questions]**

1. If you or a family member needed a translator for a medical examination, and no family member or friend was able to, do you believe you have the right to receive this assistance?
   1. Yes
   2. No
   3. Not sure
2. How often do you have trouble understanding what your doctor is saying about your health condition or how to treat it?
   1. Never **[Skip next question]**
   2. Rarely
   3. Sometimes
   4. Usually
3. Have you ever felt that your doctor was disrespectful to you in some way?
   1. Yes
   2. No **[Skip next question]**
4. How often does this happen? (doctor is disrespectful)
   1. Rarely
   2. Sometimes
   3. Usually
5. Do you ever feel that your doctor is not listening to you or does not care what you are saying when you are explaining your illness or problem?
   1. Yes
   2. No [**Skip to next question]**
6. How often does this happen? [doctor is doesn’t listen to you]
   1. Rarely
   2. Sometimes
   3. Usually
7. Has your doctor ever told you that you are cured of an illness, but you are still not cured yet?
   1. Yes
   2. No
8. Domestic violence affects many people’s health. If a man or woman is repeatedly being hit or beaten by his or her spouse, who should he or she turn to for help? **[Select all that apply]**
   a. Family members
   b. Trusted Community leaders
   c. Trusted friends
   d. An Imam or mosque leader
   e. A professional family counselor
   f. A religiously based family counselor
   g. Police
   h. A domestic violence hotline or shelter
   i. Other:_________________________________________________

**MEN’S HEALTH - The next three questions are only for men**

  Have you ever received any of the following exams?

|  | Yes, in the past 1 yr | Yes, but not in the past year | No, never | Don't know |
| --- | --- | --- | --- | --- |
| 1. Men aged 18- 35 only: Testicular exam | 1 | 2 | 3 | 99 |
| 1. Men aged 50+ only: Prostate exam | 1 | 2 | 3 | 99 |
| 1. Men aged 50+ only:   Colon cancer screening | 1 | 2 | 3 | 99 |

# Women's Health

**The next questions are only for women. If interviewee is a man, this section is over.**

1. In addition to your regular doctor, do you see an OB/GYN (Obstetrician or Gynecologist) for female-related health care, or not?
   1. Yes
   2. No [**Skip next question]**
2. Is your OB/GYN (Obstetrician or Gynecologist) male or female?
   1. Male
   2. Female
3. How many times have you been pregnant? Be sure to count all pregnancies including a current pregnancy, live births, miscarriages, stillbirths, tubal pregnancies, or abortions.______
   **[If never been pregnant skip next four questions]**
4. How many of your pregnancies resulted in live births?_______
5. At what point during your pregnancies, do you seek prenatal care?
   1. At the beginning of the pregnancy
   2. A few months into the pregnancy
   3. Near the end of the pregnancy
   4. I do not seek prenatal care
6. Did you or are you breastfeeding any of your children?
   1. Yes, all of my children **[Skip next question]**
   2. Yes, some of my children
   3. No
7. If you did not breastfeed all your children, what were the reasons for not breastfeeding? [**Select all that apply]**a. Job/scheduling difficulties
   b. Mother's physical/medical difficulties
   c. Child's physical/medical difficulties
   d. Child preferred bottle feeding
   e. Did not know how to breast-feed
   f. Other reasons _____________________________________________________

Have you ever received any of the following exams?

|  | Yes, in the past 1 yr | Yes, but not in past year | No, never | Don't know |
| --- | --- | --- | --- | --- |
| 1. Physical breast exam by health care professional | 1 | 2 | 3 | 99 |
| 1. Pap smear | 1 | 2 | 3 | 99 |
| 1. Women aged 40+ only: Mammogram | 1 | 2 | 3 | 99 |
| 1. Women aged 50+ only: Colon cancer screening | 1 | 2 | 3 | 99 |

1. Do you do regular breast self exams? If yes, how often?
   1. Yes, once a month
   2. Yes, once every 2-3 months
   3. Yes, once every 4-6 months
   4. Yes, once every 7-12 months
   5. Yes, less than once a year
   6. No
2. How familiar are you with a disease called osteoporosis or brittle bones?
   1. Very familiar
   2. Somewhat familiar
   3. Not very familiar
   4. Not Familiar at all
3. Have you used birth control to prevent pregnancy in the past year?
   1. Yes 2. No **[Skip the next four questions]**
4. What method or methods of birth control do you use? **[Select all that apply]**
   a. Injectables (like Depo-Provera shots)
   b.. Norplant (hormonal implant)
   c. Oral contraceptives (the pill)
   d. The patch
   e. Condoms
   f. Intrauterine device (IUD)
   g. Diaphram
   h. Withdrawal
   i. Spermicide
   j. Other [Explain]: _________________________________
   97. Refused
5. If a program was available, would you like to learn more about birth control from a medical professional?
   1. Yes
   2. No

Domestic violence affects many women's health. Have you experienced any of the following in your family in *the last* *two years*?

1. *Verbal abuse* (Calling you names, or swearing at you; always putting you down)
   1. Yes
   2. No
2. If yes, who has done this to you? **[select all that apply]**
   a. Your spouse
   b. A parent
   c. Once of your spouse’s parents
   d. A brother or sister
   e. A son or daughter
   f. Other relative_____________________________
3. *Physical abuse* (Such as, you were pushed or thrown to the ground, kicked, choked, bit, or hit with a fist or object.)
   1. Yes
   2. No
4. If yes, who has done this to you? **[select all that apply]**
   a. Your spouse
   b. A parent
   c. One of your spouse’s parents
   d. A brother or sister
   e. A son or daughter
   f. Other relative_____________________________
5. How often have these incidents occured?
   1. Several times/week
   2. Several times/month
   3. Several times/year
   4. Once or twice a year
   5. Happened one time only
6. Have you ever discussed these incidents with a doctor or a health care professional?
   1. Yes
   2. No

Now I have a story of a person and how his/her life is going. The story is not about a real person, but there are people like him/her. After each description I will ask you your opinion of their situation. **[If interviewee number is odd, read story #1; if even read #2 and substitute “Sahar” where it says “Wali” and switch the gender of pronouns, etc.]**

#1. Wali is an adult Afghan man living in Fremont. For the past two weeks Wali has been feeling really down. He wakes up in the morning feeling *del tang* and this feeling sticks with him all day long. He isn't enjoying things the way he normally would. In fact nothing gives him pleasure. Even when good things happen, they don't seem to make Wali happy. He pushes on through his days, but it is really hard. The smallest tasks are difficult to accomplish. He finds it hard to concentrate on anything. He feels out of energy and worn out. And even though Wali feels tired, when night comes he can't go to sleep. Wali feels pretty worthless, and very discouraged. Wali’s family has noticed that he hasn't been himself for about the last month and that he has pulled away from them. Wali just doesn't feel like talking.

#2. Sahar is an Afghan female who is 14 years old. In the last few months, Sahar has been increasingly moody, staying in her room after school, and seems to have lost interest in her favorite hobbies and in friends. Sahar says that she always feels very tired even though she is sleeping more than normal, and doesn't feel like eating. Sahar has been having trouble concentrating on what she is doing both in school and at home, and has told her parents that "I wish I hadn't been born." One of Sahar's friends has also heard (him/her) talk about committing suicide.

1. In your opinion, what is Wali’s problem called? What is its name? _____________
   _________________________________________________________________________
2. In your opinion, what do you think caused Wali’s problem? [**If interviewee says not sure or don’t know, ask “What do you think might have caused his/her problem]**_________________________________________________________________________________________________________________________________________________________________________________________________________________________________
3. In your opinion, how serious is Wali’s problem --very serious, somewhat serious, not very serious, or not at all serious?
   1. Very serious
   2. Somewhat serious
   3. Not very serious
   4. Not at all serious
   98. DK
   99. Refused
4. In your opinion, should Wali get help for this condition?
   1. Yes
   2. No
   98. DK
   99. Refused
5. with this is betterme, and there may be a couple that aren'____________________________________________________If yes, who would you recommend that Wali/Sahar get assistance from? Please list up to three in order of importance.
   1. ___________________________________________
   2. ___________________________________________
   3.____________________________________________
6. How would #1 help Wali/Sahar? ____________________________________________
   ______________________________________________________________________________________________________________________________________________________
   How would #2 help Wali/Sahar?_______________________________________________
   ______________________________________________________________________________________________________________________________________________________
7. How would #3 help Wali/Sahar?_______________________________________________
   ______________________________________________________________________________________________________________________________________________________
8. In your opinion, if Wali/Sahar receives the assistance you recommend, how likely is it that he/she will recover and have a normal life?
   1. Very unlikely
   2. Not very likely
   3. Somewhat likely
   4. Very likely
9. In your opinion, what are the main problems this will cause for him/her and his/her family?
   _________________________________________________________________________________________________________________________________________________________________________________________________________________________________
10. In your opinion, how likely is it that Wali is experiencing *part of the normal ups and downs of life* --very likely, somewhat likely, not very likely, or not at all likely?
    1. Very likely
    2. Somewhat likely
    3. Not very likely
    4. Not at all likely
    98. DK
    99. Refused
11. In your opinion, how likely is it that Wali is experiencing *a mental illness*--very likely, somewhat likely, not very likely, or not at all likely?
    1. Very likely
    2. Somewhat likely
    3. Not very likely
    4. Not at all likely
    98. DK
    99. Refused
12. Here is a list of things that Wali could do that might help him/her. Which of these do you think Wali should definitely do? **[Select all that apply]**
    a. Talk things over with family
    b. Get help from an Imam or other religious leader
    c. Try praying five or more times a day
    d. Get help from a medical doctor
    e. Go to a therapist, or counselor, like a psychologist, social worker, or other mental health professional
    f. Go to a spiritual healer or natural healer like a sayeed
    g. Go to someone to help remove the curse
    h. Join a support group where people with similar problems help each other
    i. Take non-prescription medicines (over the counter) like sleeping pills
    j. Take prescription medicines
    k. Take herbal medicines
    l. Try to relax or go on a vacation
13. Should Wali tell his/her friends about his condition?
    1. Definitely yes
    2. Probably yes
    3. Probably no
    4. Definitely no
    98. DK
    99. Refused
14. What would most Afghans you know think or say about him when they learn of his condition?________________________________________________________________ _________________________________________________________________________
    _________________________________________________________________________
15. How willing would most Afghans you know be to *make friends with* (Wali/Sahar)-- definitely willing, probably willing, probably unwilling, or definitely unwilling?
    1. Definitely willing
    2. Probably willing
    3. Probably unwilling
    4. Definitely unwilling
    98. DK
    99. Refused

Now I will list some problems and complaints that people sometimes have when they are under stress. Please tell HOW MUCH DISCOMFORT THAT PROBLEM HAS CAUSED YOU DURING THE PAST MONTH INCLUDING TODAY. The answers for each problem [show CARD__] are:
 0 = Not at all; 1 = A little bit; 2 = Moderately; 3 = Quite a bit; 4 = Extremely

1. ___ Trouble remembering things
2. ___Feeling easily annoyed or irritated
3. ___Pains in heart or chest
4. ___Feeling that most people cannot be trusted
5. ___ Temper outbursts that you could not control
6. ___ Feeling lonely even when you are with people
7. ___ Your feelings being easily hurt
8. ___ Feeling that people are unfriendly or dislike you
9. ___ Difficulty making decisions
10. ___ Getting into frequent arguments
11. ___ Others not giving you proper credit for your achievements
12. ___ Feelings of worthlessness
13. ___ Feelings of guilt

Now I will list some other problems that people sometimes have when they are under stress. Please tell me HOW OFTEN IN THE LAST MONTH YOU HAVE EXPERIENCED THESE. The answers for each problem [show CARD__ ] are:

0 = Never; 1 = Almost never; 2 = Sometimes; 3 = Fairly often; 4 = Very often;

1. ___ During the past month, how often have you had attacks of sudden fear or panic?
2. ___During the past month how often have you become jigar khun?
3. ___ During the past month, how often have you been bothered by feelings of sadness or depression -- feeling blue?
4. ___ During the past month, how often have you been bothered by nervousness, being fidgety or tense?
5. ___ During the past month, how often have you had headaches?

0 = Never; 1 = Almost never; 2 = Sometimes; 3 = Fairly often; 4 = Very often;

1. ___During the past month, how often have you spent your time in *churt?*
2. ___ During the past month, how often have you felt *del tang?*
3. ___ During the past month, how often have you felt useless?
4. ___ During the past month, how often have you felt anxious?
5. ___ During the past month, how often have you been *asabi*?
6. ___ During the past month, how often have you felt that nothing turns out for you the way you want it to?
7. ___ During the past month, how often have you felt completely hopeless about everything?
8. ___ During the past month, how often have you felt completely helpless?

**[Remind interviewee that these are problems people have when they are under a great deal of stress.]**

1. ___ During the last 2 weeks, how many times have you felt *fishar Bala* or *fishar payin?*
2. ___ During the past month, how often have you had times when you couldn't help wondering if anything was worthwhile any more?
3. ___ During the past month, how often have you had trouble concentrating or keeping your mind on what you were doing?
4. ___ During the past month, how often have you beaten or hurt yourself?
5. ___ During the past month, how often have you beaten someone in your family?
6. ___ In general, how satisfied have you been with yourself during the last year?
   0. Very satisfied
   1. Somewhat satisfied
   3. Somewhat dissatisfied
   4. Very dissatisfied

Please consider the following reactions which sometimes occur even long after a traumatic or life threatening event or events. These questions are about your personal reactions to *a life threatening event or events* that you have experienced? Please indicate (Yes/No) whether or not you have experienced any of the following *at least twice in the past week*. **[Yes = 1; No = 2]**

1. ___ Upsetting thoughts or memories about an event that have come into your mind against your will
2. ___ Upsetting dreams about an event
3. ___ Acting or feeling as though an event were happening again
4. ___ Feeling upset by reminders of a traumatic event
5. ___ Bodily reactions (such as fast heartbeat, stomach churning, sweatiness, dizziness) when reminded of an event
6. ___ Difficulty falling or staying asleep
7. ___ Heightened awareness of potential dangers to yourself and others
8. ___ Being jumpy or being startled at something unexpected

Finally we have a couple of questions about “Little Kabul” that we would like your opinion on.

1. Have you heard the name “Little Kabul?”
   1. Yes
   2. No
2. What is “Little Kabul”?______________________________________________________
   __________________________________________________________________________________________________________________________________________________
3. "Do you think non-Afghans are favorable or unfavorable towards the term "Little Kabul."
   1. Favorable
   2. Don’t care
   3. Unfavorable.
4. Do you support developing a tourist attraction in Fremont named "Little Kabul" that showcases Afghan cultures, art, music, history, food and hospitality?"
   1. Yes
   2. Not sure
   3. No

THANK YOU! All of us on the research team thank you for helping us document the most important needs of the Afghan community.
